# HPV8-induced STAT3 activation led keratinocyte stem cell expansion in human actinic keratoses

**DOI:** 10.1172/jci.insight.177898

**Published:** 2024-06-25

**Authors:** Huw J. Morgan, Carlotta Olivero, Boris Y. Shorning, Alex Gibbs, Alexandra L. Phillips, Lokapriya Ananthan, Annabelle Xiao Hui Lim, Licia Martuscelli, Cinzia Borgogna, Marco De Andrea, Martin Hufbauer, Richard Goodwin, Baki Akgül, Marisa Gariglio, Girish K. Patel

**Affiliations:** 1 European Cancer Stem Cell Research Institute, School of Biosciences, Cardiff University, Cardiff, United Kingdom.; 2 Department of Translational Medicine, University of Eastern Piedmont, Novara, Italy.; 3 Viral Pathogenesis Unit, Department of Public Health and Pediatric Sciences, University of Turin Medical School, Turin, Italy.; 4 Intrinsic Immunity Unit, Translational Research Centre for Autoimmune and Allergic Diseases, University of Eastern Piedmont, Novara, Italy.; 5 Institute of Virology, University of Cologne, Medical Faculty and University Hospital Cologne, Cologne, Germany.; 6 Department of Dermatology, Aneurin Bevan University Health Board, Royal Gwent Hospital, Newport, United Kingdom.

**Keywords:** Cell biology, Stem cells, Adult stem cells, Mouse models, Skin cancer

## Abstract

Despite epidermal turnover, the skin is host to a complex array of microbes, including viruses, such as HPV, which must infect and manipulate skin keratinocyte stem cells (KSCs) to survive. This crosstalk between the virome and KSC populations remains largely unknown. Here, we investigated the effect of HPV8 on KSCs using various mouse models. We observed that the HPV8 early region gene E6 specifically caused Lrig1^+^ hair follicle junctional zone KSC proliferation and expansion, which would facilitate viral transmission. Within Lrig1^+^ KSCs specifically, HPV8 E6 bound intracellular p300 to phosphorylate the STAT3 transcriptional regulatory node. This induced ΔNp63 expression, resulting in KSC expansion into the overlying epidermis. HPV8 was associated with 70% of human actinic keratoses. Together, these results define the “hit-and-run” mechanism for HPV8 in human actinic keratosis as an expansion of KSCs, which lack melanosome protection and are thus susceptible to sun light–induced malignant transformation.

## Abstract 2

Crosstalk between HPV8 and skin keratinocyte stem cells promotes host cell proliferation and expansion, which in turn facilitates further viral transmission in actinic keratosis.

## Graphical abstract

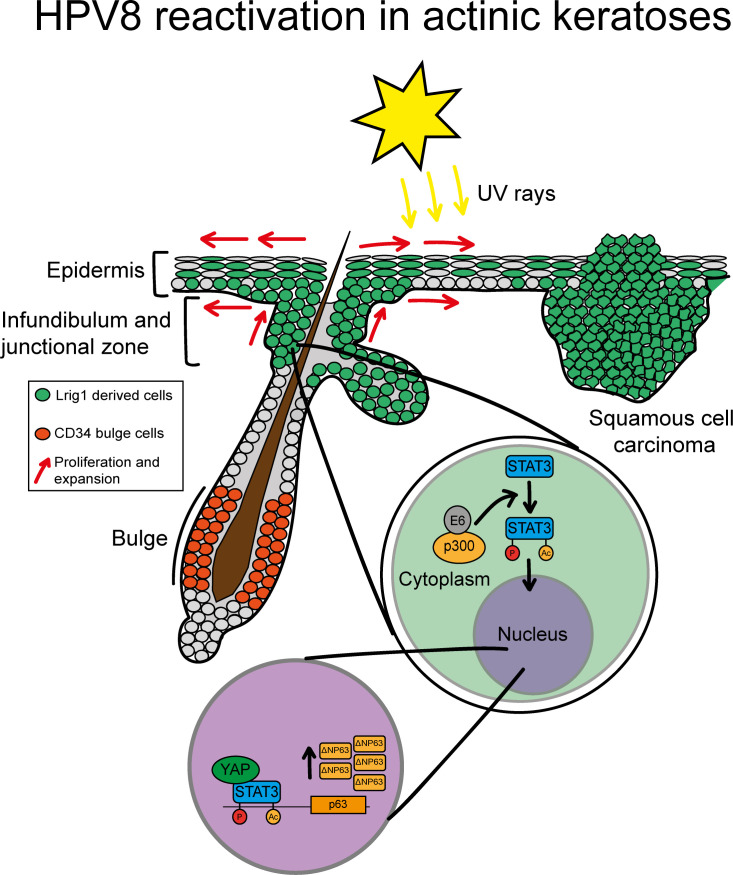

## Introduction

Human skin hosts a microbiota that has maintained symbiosis through evolution. Integral to this environmental interface is a large and diverse array of viruses, the virome, which is capable of manipulating host cellular processes to reside as a symbiont (1–3). As obligate intracellular parasites, notably *Papillomaviridae* have to infect long-lived cells, such as skin keratinocyte stem cells (KSCs), to withstand constant epidermal turnover (4). Among the various skin KSC populations, the hair follicle (HF) KSCs have been implicated as host cells for *Beta-papillomaviruses* (5–7). Recently, HF KSCs have been identified as important regulators in the crosstalk between the bacterial microbiome and the immune system (8–11). The virome is unaffected by antibiotics, yet similar to the bacterial microbiome, expansion and diversification are observed with impaired immunity. Relative to healthy individuals, subtle shifts in immune function such as in epidermodysplasia verruciformis (EV) or dedicator of cytokinesis 8 deficiency, directly alter the virome, leading to increased diversity and expanded representation of skin tropic β and γ HPV types, which in turn increase the risk of skin cancer (12–15). Similarly, alterations in virome have been observed among solid organ transplant recipients on immunosuppression, who exhibit a 60- to 250-fold increased risk of skin cancer (16–22). As such, there is a clinical imperative to elucidate the crosstalk between HPV and host KSCs.

The *Papillomaviridae*, members of the HPV family, are small (7–8 kb) nonenveloped DNA viruses that undergo episomal replication within differentiating keratinocytes. HPV contains three distinct coding regions: (a) an upstream regulatory region, (b) an early region with typically up to 6 open reading frames (E1, E2, E4, E5, E6 and E7), and (c) a late region encoding capsid proteins L1 and L2 (23, 24). Tissue tropism is determined by the L1 protein, with negatively charged L1 protein of β and γ HPV types selectively targeting human nonmucosal skin, while the positively charged L1 protein on α HPV types results in mucosal infection (24, 25). Microabrasion facilitates viral entry into basal keratinocytes, and therein KSCs, in order to establish long-term infection, wherein E1 and E2 proteins support replication of the viral genome at a low copy number. It is only when these infected basal cells differentiate, thus moving closer to the surface, that the viral load increases to support viral transmission (26).

In the majority, HPV infection is an asymptomatic infection that may result in transient warts or keratoses, countered by the immune response that blocks viral replication. However, persistent infection with high-risk HPV types, notably α HPV, is associated with cancer, accounting for an estimated 600,000 cases per annum (27). In α HPV, viral integration into the host DNA is postulated to deregulate expression of E6 and E7 proteins, leading to keratinocyte transformation; however, transformation can also occur without integration (28). In the complex structure of the skin with appendageal structures, high-risk β HPV (HPV5, -8, and -38) have been associated with premalignant skin changes, called actinic keratoses (AKs), which in association with UV light exposure, risk transformation to cutaneous squamous cell carcinoma (cSCC) (16–18, 29–36). However, β HPV DNA integration is not observed, and the viral load in ensuing cSCC is low, leading authors to postulate a “hit-and-run” mechanism for transformation (30, 37).

Consistent with the potential oncogenic role of high-risk β HPV, FVBN-transgenic mice expressing HPV8 early region genes under the control of the keratin 14 (Krt14) promoter (HPV8-CERtg) exhibit skin changes mirroring human AK and spontaneously develop cSCC, which occurs with greater frequency after UV light exposure (38, 39). HPV8-CERtg mice crossed with Rag2-deficient mice, to recreate the immunosuppressive tumor microenvironment similar to that observed in organ transplant recipients on immunosuppressants, demonstrated accentuated tumor growth (40). In addition, cSCC has also been observed in transgenic mice expressing individual HPV8 early region genes E2, E6, and E7 under the control of the Krt14 promoter (41–43). Recently, we found that the HF junctional zone KSC (herein denoted as JZSC) population, defined by the expression of leucine-rich repeats and immunoglobulin-like domains protein 1 (Lrig1) on the cell surface, was selectively expanded in HPV8-CERtg mice (38). In this article, we elucidate the crosstalk between HPV8 and KSCs, redefining the basis for AK, and thus identify HPV8 to be a major risk factor for human cSCC.

## Results

**Figure 1:**
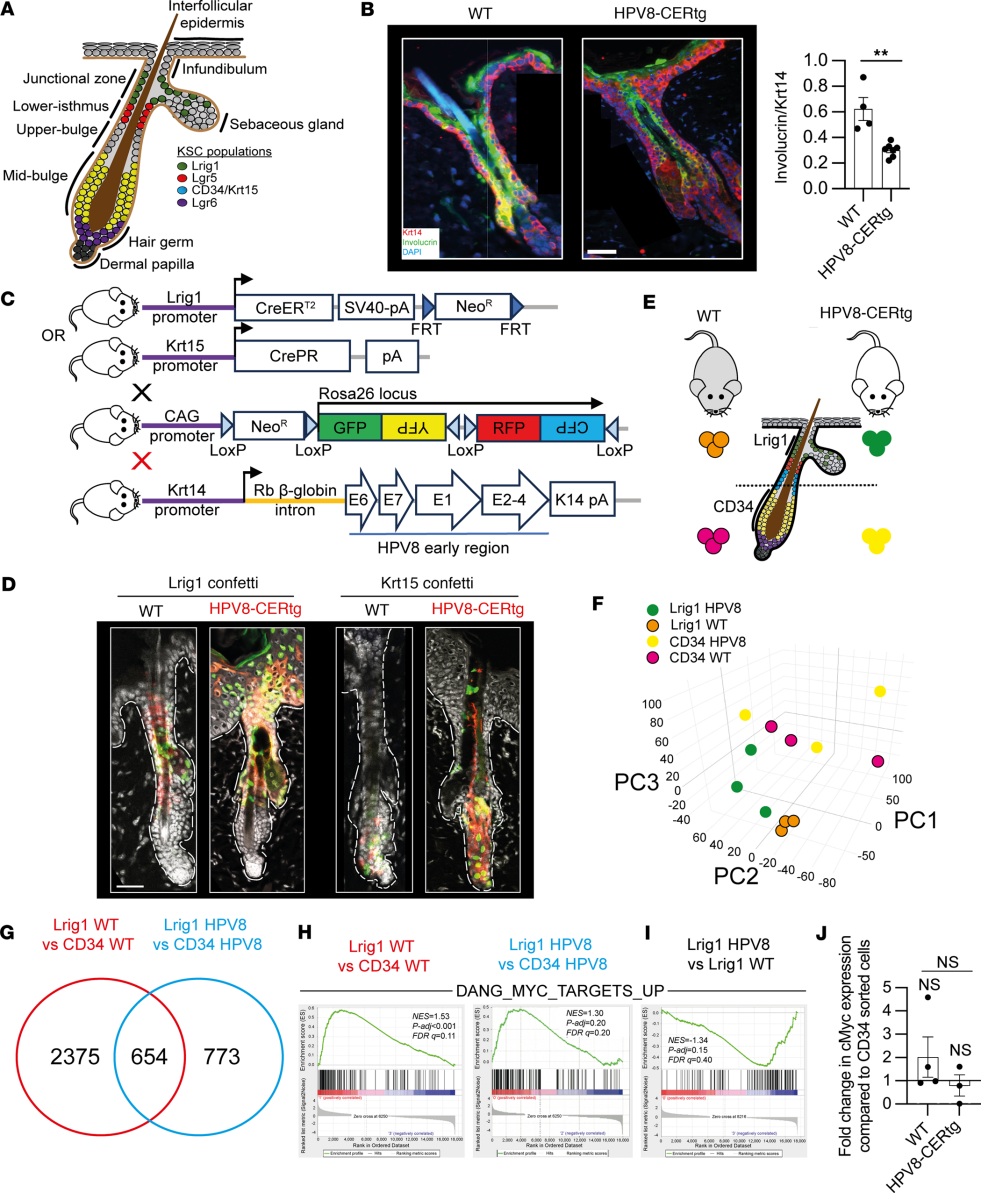
HPV8 induced Lrig1^+^ hair follicle junctional zone KSC proliferation and expansion. (**A**) Schematic of hair follicle KSC populations. (**B**) Immunofluorescence labeling of WT (left) and HPV8-CERtg (right) adult back skin for involucrin (green) and keratin 14 (red). *n* = 11 mice (average of 10 hair follicles/mouse). Scale bar: 40 μm. (**C**) Schematic summary of 4 mouse lines that were crossed for lineage tracing. (**D**) CLSM was used to visualize Lrig1 (left) and Keratin 15 (right) promoter-driven Confetti reporter expression in progeny of WT (left) and HPV8-CERtg (right) adult mice. Scale bar: 40 μm. (**E**) Experimental strategy for flow-sorting Lrig1^+^ and CD34^+^ populations for within-mouse comparisons. See also Supplemental Figure 1. (**F**) PCA of RNA-Seq transcriptome analysis of skin KSC populations. (**G**) Venn diagram showing shared DEGs from Lrig1 versus CD34 comparisons from WT and HPV8 mice (see Supplemental Table 1). (**H**) GSEA for c-Myc–regulated genes in DEGs from transcriptomic analysis. (**I**) GSEA for c-Myc–regulated genes was undertaken on DEGs from Lrig1 flow-sorted HPV8-CERtg versus WT transcriptomic analysis. (**J**) qPCR of RNA from flow-sorted cells isolated as in **E**. See also Supplemental Figure 1. Statistical tests included (**B**) 2-tailed Student’s *t* test and (**J**) 1-way ANOVA. **P < 0.01.

The HF contains multiple KSC populations that under homeostatic conditions maintain keratinocyte numbers during hair cycling but also retain the capacity to regenerate the whole HF (Figure 1A). To determine how HPV8 selectively drives the proliferation and expansion of the Lrig1^+^ JZSC population we utilized the HPV8-CERtg mouse model, wherein the Krt14 promoter regulates the expression of the HPV8 early region genes in basal cells of the entire epithelium (Supplemental Figure 1A; supplemental material available online with this article; https://doi.org/10.1172/jci.insight.177898DS1). Immunofluorescence labeling of skin with antibodies binding to the basal keratin Krt14 and the differentiation marker involucrin showed that the HPV8-CERtg compared with WT mice had an expansion of the undifferentiated keratinocytes within the infundibulum and overlying epidermis, with a reduction in the involucrin/Krt14 ratio (Figure 1B). To confirm that Lrig1^+^ JZSC proliferation led to expansion into the infundibulum and overlying epidermis, we crossed Lrig1CreER^T2^:R26RConfetti and Krt15CrePGR:R26RConfetti mice with HPV8-CERtg mice (Figure 1C). The Confetti mouse contains a 4-color cassette, which recombines within individual cells upon Cre activation to express 1 of 4 fluorescent proteins: green, red, yellow, or cyan (44). In WT Lrig1CreER^T2^:R26RConfetti and Krt15CrePGR:R26RConfetti mice, lineage-labeled progeny, as expected, remained compartmentalized to the infundibulum and sebaceous gland (Lrig1CreER^T2^:R26RConfetti mice) or to the lower HF and inner root sheath (Krt15CrePGR:R26RConfetti mice) (Figure 1D). Likewise Krt15CrePGR:R26RConfetti:HPV8-CERtg mice showed a similar distribution of labeled cells compared with their WT counterparts. However, Lrig1CreER^T2^:R26RConfetti:HPV8-CERtg mice showed fluorescent clones extending into the infundibulum and perifollicular epidermis (Figure 1D). Thus, HPV8-induced selective proliferation of the Lrig1^+^ JZSC population resulted in the expansion of this population into the infundibulum and perifollicular epidermis.

### c-MYC transcriptional regulation distinguishes JZSCs from HF bulge KSCs during homeostasis.

Lrig1^+^ JZSCs are transcriptionally distinct from the lower HF bulge KSC populations, which are typically characterized by CD34 cell surface expression (38, 45, 46). To determine why HPV8 induced selective proliferation of the Lrig1^+^ JZSCs, but not other KSCs, we undertook a transcriptomic analysis of these adjacent HF KSC populations defined by Lrig1 and CD34 expression in adult mice. Both Lrig1^+^ and CD34^+^ flow-sorted keratinocytes were isolated from dorsal back skin of individual mice, using established protocols, for pairwise comparison (Figure 1E). Illumina HiSeq4000 paired-end sequenced samples resulted in a total of 35,566,700 reads, of which 47% mapped to 54,658 murine genes (GRCm38). Principal-component analysis distinguished both genotype and KSC population transcriptomes (Figure 1F). Unsupervised hierarchical clustering of log_2_-transformed, median-centered, average linkage by Pearson’s correlation showed primary segregation of KSC populations with the influence of HPV8 as a secondary separation (Supplemental Figure 1B), suggesting that the regulatory signaling pathway networks between adjacent KSC populations are largely distinct and independent of the effects of HPV8 early region genes. Normalization of count data and DESeq2 pipeline analysis identified 3,029 differentially expressed genes (DEGs; *P* < 0.05) from Lrig1^+^ versus CD34^+^ flow-sorted keratinocytes in WT mice (Figure 1G and Supplemental Table 1). These DEGs were enriched for 330 genes that distinguish Lrig1^+^ from CD34^+^ KSC populations previously identified by microarray analysis (Supplemental Figure 1C) (46). Gene set enrichment analysis (GSEA) (47, 48) of the DEGs determined that the Lrig1^+^ JZSCs were involved in upper HF and sebaceous gland differentiation (positive enrichment) and were distinct from the CD34^+^ outer layers (negative enrichment) of the HF bulge keratinocytes (Supplemental Figure 1D). GSEA identified c-Myc as the central regulating node that distinguished these two KSC populations, in WT and HPV8-CERtg mice, with Lrig1^+^ JZSC flow-sorted keratinocytes showing a greater enrichment of the *c-Myc* signature when compared with the CD34 population, consistent with the role of the JZSCs in maintaining sebaceous gland sebocyte differentiation (Figure 1H). Thus, Lrig1 JZSCs under homeostatic conditions represent a distinct HF KSC population distinguished by activation of c-Myc.

Transcriptomic analysis of flow-sorted Lrig1^+^ and CD34^+^ KSC populations from adult HPV8-CERtg mice yielded 1,427 DEGs, of which 46% overlapped with DEGs from similar analysis in WT mice (654 genes), consistent with the primary segregation found in our unsupervised hierarchical clustering. Bioinformatic analysis of HPV8-CERtg flow-sorted cells demonstrated findings that were similar to WT mice, but there was no further enrichment of *c-Myc* to account for increased proliferation from GSEA (Figure 1I) or difference in transcript counts or *c-Myc* gene expression by quantitative real-time PCR (qPCR) (Figure 1J). Therefore, c-Myc activation differentiates Lrig1^+^ and CD34^+^ KSC populations in both WT and HPV8-CERtg HFs but was not responsible for the HPV8-induced proliferation of only the Lrig1^+^ JZSC population.

### STAT3 activation drives Lrig1 JZSC proliferation.

**Figure 2:**
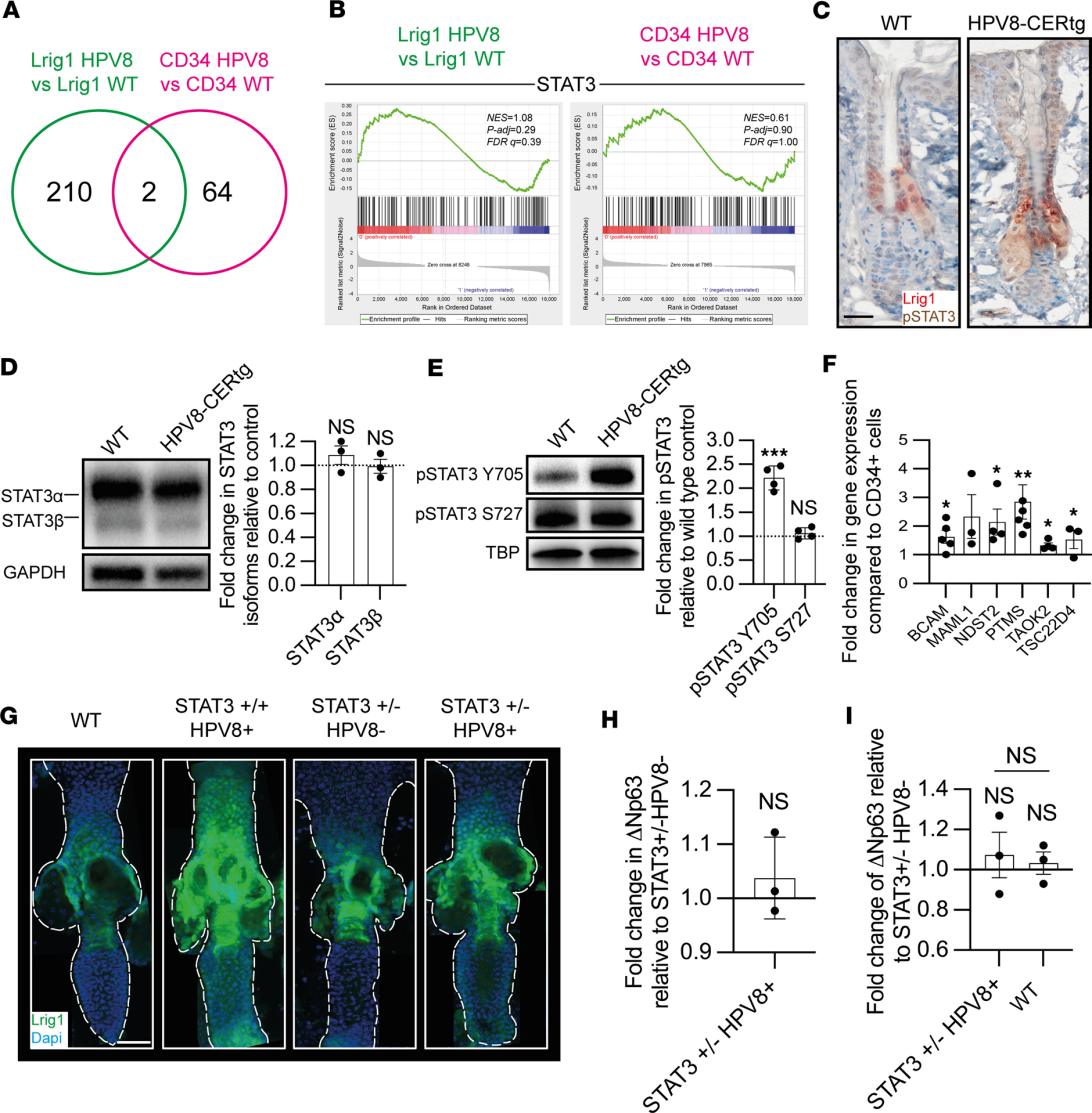
Activated STAT3 regulatory node in HPV8 in Lrig1^+^ hair follicle junctional zone KSC. (**A**) Venn diagram showing shared DEGs from HPV8-CERtg versus WT KSC comparisons. (**B**) GSEA of STAT3-regulated genes in DEGs from transcriptomic analysis. See also Supplemental Figure 2. (**C**) IHC for pSTAT3 on adult back skin from WT and HPV8-CERtg mice. Scale bar: 40 μm. (**D**) Immunoblot of total STAT3 (α and β isoforms) in WT and HPV8-CERtg adult back skin epidermal sheet extracts (*n* = 3). Dotted line is the comparator. (**E**) Immunoblot of pSTAT3 Y705 and S727 in WT and HPV8-CERtg adult back skin epidermal sheet nuclear extracts (*n* = 4). Dotted line is the comparator. (**F**) qPCR of RNA from flow-sorted cell isolates, as in Figure 1E, for STAT3 downstream target genes (*n* ≥ 3). (**G**) CLSM of whole-mount tail skins from WT, HPV8-CERtg, STAT3^+/–^, and STAT3^+/–^ HPV8-CERtg mice for Lrig1 (green) with DAPI (blue). Scale bar: 40 μm. (**H**) qPCR of RNA from STAT3^+/–^ and STAT3^+/–^ HPV8-CERtg adult back skin epidermal sheets for ΔNp63 (*n* = 3). (**I**) qPCR of RNA from WT, STAT3^+/–^, and STAT3^+/–^ HPV8-CERtg flow-sorted Lrig1^+^ KSCs for ΔNp63 (*n* = 3). Statistical tests included (**D**–**F** and **H**) 2-tailed Student’s *t* test and (**I**) 1-way ANOVA. *P < 0.05; **P < 0.01; ***P < 0.001.

While the bioinformatic analysis comparison of Lrig1^+^ and CD34^+^ HF KSC populations yielded a total of 3,802 DEGs (Figure 1G), in contrast, there were a total of 276 DEGs (*P* < 0.05) from the analysis of HPV8-CERtg versus WT HF KSC populations (Figure 2A). There were only 2 shared genes, β-1,4-galactosyltransferase 6 and olfactory receptor family 12 subfamily D member 3, suggesting that the transcriptional impact of HPV8 early region genes was unique to individual KSC populations, even when closely situated, consistent with the observed increased proliferation of the Lrig1^+^ JZSC but not the CD34 bulge KSC population.

We hypothesized that HPV8 must selectively activate a growth factor pathway(s) in the Lrig1^+^ JZSC but not the CD34 bulge KSC population for there to be selective proliferation. Therefore, we utilized Ingenuity Pathway Analysis canonical pathways comparative software package (Qiagen), inputting all DEGs (adjusted *P* < 0.05, Supplemental Table 1) from comparisons of HPV8 Lrig1 versus WT Lrig1 mice and HPV8 CD34 versus WT CD34 mice. Only the STAT3 signaling canonical pathway demonstrated activation and reached significance (*P* < 0.05) with differential expression in the Lrig1^+^ HPV8 versus WT KSC populations when compared with the CD34^+^ HPV8 versus WT KSC population analyses (Supplemental Figure 1E). There was greater phosphorylated STAT3 observed within HF keratinocytes from HPV8-CERtg mouse skin (Supplemental Figure 1F). Although the glucocorticoid receptor, HIF1-α, and Hippo signaling pathways also demonstrated differential expression, these did not reach significance. GSEA of the STAT3 gene signature in the Lrig1 HPV8 versus Lrig1 WT mouse DEGs demonstrated a normalized enrichment score of 1.1, whereas the CD34 HPV8 versus CD34 WT comparison was only 0.6 (Figure 2B). Nuclear labeling of phosphorylated STAT3 was evident within the HF junctional zone, infundibulum, and adjoining interfollicular epidermis of HPV8tg skin, consistent with its role in driving Lrig1^+^ JZSC proliferation and expansion (Figure 2C). Western blot analysis of HPV8-CERtg and WT mouse back skin keratinocytes identified similar levels of full-length and transcriptionally active STAT3α (86 kDa) as the predominant splice variant (Figure 2D). Consistent with activation of the STAT3 pathway, HPV8-CERtg mouse skin nuclear fractions demonstrated a 2.2-fold increase in Tyr705 STAT3 phosphorylation (within the transactivation domain) but no change in Ser727 STAT3 phosphorylation (within the COOH terminus) (Figure 2E). STAT3 downstream transcriptionally regulated genes were increased in expression by qPCR in HPV8-CERtg Lrig1^+^ JZSCs relative to the CD34^+^ bulge KSC population (Figure 2F).

To determine if the STAT3 regulatory node was essential for HPV8-induced Lrig1^+^ JZSC proliferation, we crossed HPV8-CERtg mice with Krt5Cre-Stat3^+/fl^ mice to generate HPV8-CER:STAT3^+/–^tg mice, since STAT3 knockout is known to be embryologically lethal. We have previously shown that HPV8-CER:STAT3^+/–^tg mice had WT levels of Tyr705 STAT3 phosphorylation and demonstrated a 4-fold reduction in tumor formation (49). Confocal laser scanning microscopy (CLSM) imaging using IMARIS 3D-rendering software of fluorescently labeled Lrig1^+^ JZSCs in whole-mount tail skin showed no expansion in HPV8-CER:STAT3^+/–^tg mice (Figure 2G). Consistent with the lack of KSC proliferation, Δ*Np63* expression levels in HPV8-CER:STAT3^+/–^tg skin were comparable to those in WT skin (Figure 2H), specifically also in the Lrig1^+^ flow-sorted cells (Figure 2I). Similarly, Δ*Np63* expression levels in the HPV8-CER:STAT3^+/–^tg flow-sorted CD34^+^ bulge KSCs remained unchanged (Supplemental Figure 1G). In summary, HPV8-induced Lrig1^+^ JZSC proliferation was dependent upon STAT3 Tyr705 phosphorylation.

### HPV8 E6–induced proliferation of Lrig1 JZSCs.

**Figure 3:**
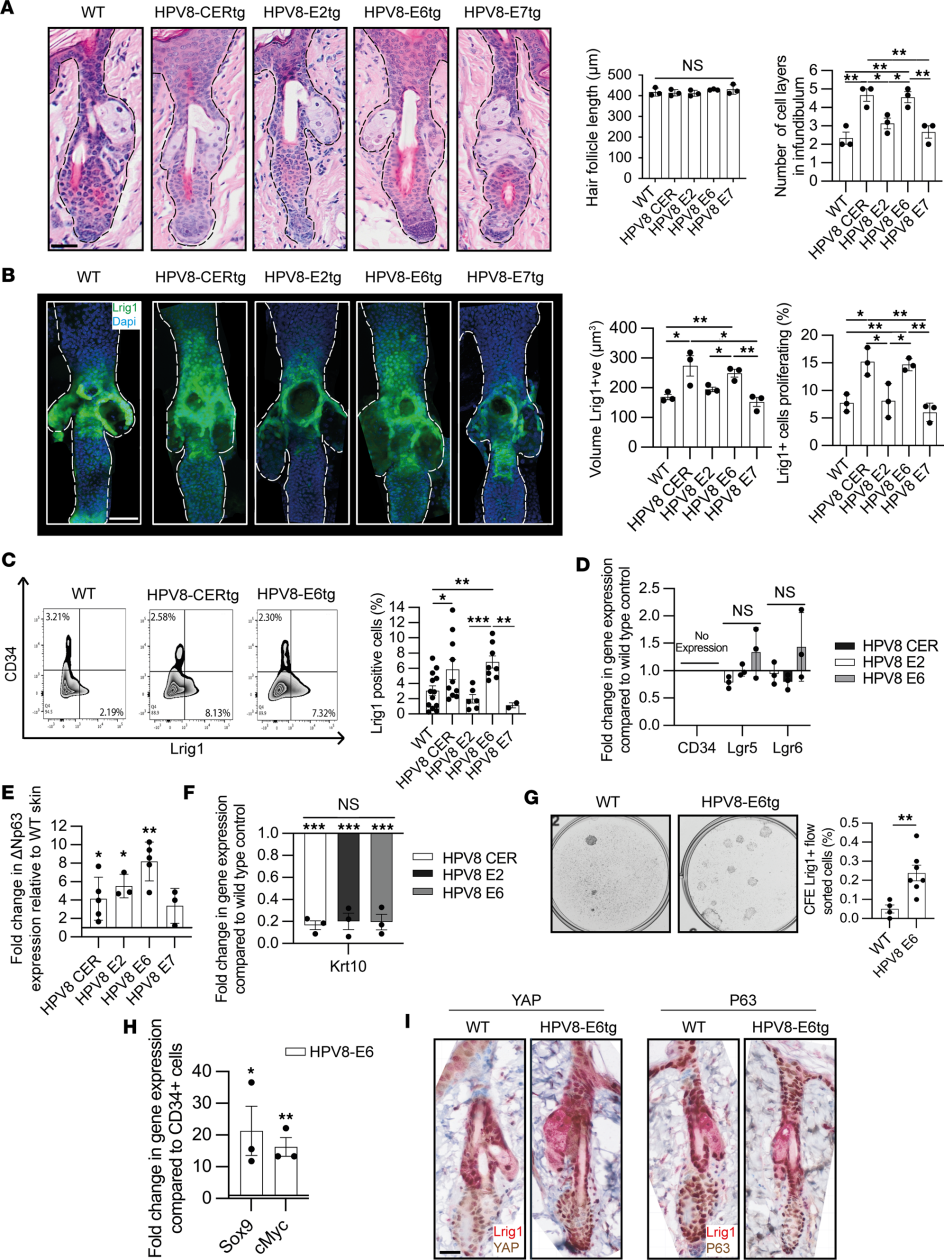
HPV8 E6 drives Lrig1^+^ hair follicle junctional zone KSC proliferation and expansion. (**A**) H&E-stained sections from WT, HPV8-CERtg, HPV8-E2tg, HPV8-E6tg, and HPV8-E7tg adult back skin, with quantification of hair follicle length and number of cell layers in the infundibulum (*n* = 3 mice/genotype, average of 20–50 hair follicles/mouse). Scale bar: 40 μm. (**B**) CLSM of whole-mount tail skins as in **A** labeled for Lrig1, with quantification of Lrig1-labeled volume and the number of colabeled Ki67^+^ cells (average of 10 hair follicles/mouse). Scale bar: 40 μm. (**C**) FACS for Lrig1^+^ and CD34^+^ populations from back skin cell isolates as in **A** (*n* = 39 total). (**D**) qPCR of RNA from Lrig1^+^ flow-sorted cell isolates as in **A** for KSC markers (*n* = 3). (**E**) qPCR of RNA from Lrig1^+^ flow-sorted cell isolates as in **A** for ΔNp63 (*n* = 20 total). (**F**) qPCR of RNA from Lrig1^+^ flow-sorted cell isolates as in **A** for keratin 10 (*n* = 3). (**G**) CFE of 2,500 flow-sorted Lrig1^+^ keratinocytes from WT and HPV8-E6tg adult back skin epidermal sheets (*n* = 11 total). (**H**) qPCR of RNA from Lrig1^+^ and CD34^+^ flow-sorted cell isolates from HPV8-E6tg adult back skin epidermal sheets (*n* = 3). (**I**) IHC for Lrig1 together with YAP (left) and p63 (right) on WT and HPV8-E6tg adult back skin. Scale bar: 40 μm. See also Supplemental Figure 2. Statistical tests included (**A**–**F**) 1-way ANOVA and (**G** and **H**) 2-tailed Student’s *t* test. *P < 0.05; **P < 0.01; ***P < 0.001.

To determine which of the HPV8 early region protein(s) was responsible for STAT3 activation and therefore specific proliferation of the Lrig1^+^ JZSC population, we compared the adult mouse skin HFs from WT mice, HPV8-CERtg mice, and mice expressing the individual early region genes: HPV8-E2tg, HPV8-E6tg, and HPV8-E7tg (Figure 3A). Anagen HF lengths were similar between WT mice and the different mouse genotypes: WT (418.20 ± 11.25 μm), HPV8-CERtg (414.38 ± 9.01 μm), HPV8-E2tg (412.26 ± 8.44 μm), HPV8-E6tg (429.56 ± 1.85 μm), and HPV8-E7tg (430.43 ± 13.31 μm) (*n* > 100 HFs per genotype in 3 mice per genotype) (Figure 3A). The expanded infundibulum area observed in the HPV8-CERtg (4.67 ± 0.33 cells) compared with WT (2.33 ± 0.33 cells) was also observed in the mice with individual early region genes E2 (3.13 ± 0.29 cells) and E6 (4.56 ± 0.29 cells) but not E7 (2.67 ± 0.33 cells) (Figure 3A). Next, we fluorescently labeled Lrig1^+^ JZSCs and used CLSM with IMARIS 3D-rendering software of whole-mount tail skin from HPV8-CERtg and WT mice expressing individual early region genes (Figure 3B). Compared with WT mice (168.79 ± 9.06 μm^3^) the Lrig1^+^ population volume in HPV8-CERtg (273.51 ± 733.89 μm^3^) and HPV8-E6tg alone mice (248.94 ± 12.86 μm^3^) was significantly larger (Figure 3B). In contrast, HPV8-E2tg (194.70 ± 7.54 μm^3^) and HPV8-E7tg mice (152.30 ± 15.49 μm^3^) demonstrated no significant increase in the Lrig1^+^ JZSC population. The CD34^+^ bulge KSC population was unchanged in the transgenic mice from that observed in WT mice (Supplemental Figure 2A). Proliferation, assessed by ki67 immunofluorescence labeling, was significantly greater in the Lrig1^+^ JZSCs in HPV8-CERtg (15.00% ± 4.51%) and HPV8-E6tg mice (14.50% ± 2.56%) compared with WT mice (7.60% ± 4.08%), whereas no increase was observed in HPV8-E2tg (8.00% ± 4.34%) and HPV8-E7tg (6.88% ± 2.10%) mice (Figure 3B and Supplemental Figure 2B). As expected, there was no increase in proliferation within the CD34^+^ HF bulge KSC population compartment in the transgenic mice compared with that in WT mice (Supplemental Figure 2A).

The Lrig1^+^ JZSC population was quantified by flow cytometry in WT and transgenic mouse dorsal back skin keratinocytes labeled with Lrig1 and CD34. In contrast to the unchanged CD34^+^ population, the Lrig1^+^ population was greater in HPV8-CERtg (5.85% ± 1.32%) and HPV8-E6tg (6.84% ± 0.80%) transgenic mice compared with WT (3.05% ± 0.65%), HPV8-E2tg (1.98% ± 0.56%), and HPV8-E7tg mice (1.130% ± 0.32%) (Figure 3C and Supplemental Figure 2B). Flow-sorted Lrig1^+^ keratinocytes from transgenic and WT mice did not express *Cd34* and, similarly, had low expression levels of other HF KSC markers *Lgr5* and *Lgr6* (Figure 3D). Consistent with KSC proliferation and enrichment, the Lrig1 population from HPV8-CERtg and HPV8-E6tg mice exhibited increased expression of Δ*Np63* relative to WT mice (Figure 3E) and reduced expression of differentiation-associated *Krt10* (Figure 3F). The reduction in *Krt10* expression was not observed in the CD34 population from the same mice when compared with WT mice (Supplemental Figure 2C). Flow-sorted Lrig1^+^ keratinocytes demonstrated a 4-fold increase in colony-forming efficiency (CFE) in the HPV8-E6tg Lrig1^+^ population (0.24% ± 0.11%) compared with WT mice (0.05% ± 0.04%) (Figure 3G). Consistent with the E6-driven Lrig1^+^ JZSC proliferation and expansion, the flow-sorted Lrig1^+^ JZSCs retained elevated expression of *Sox9* and *c-Myc* compared with CD34^+^ KSCs (Figure 3H and Supplemental Figure 2D). HPV8-E6tg mouse tissue sections had increased KSC proliferation-associated proteins YAP and p63 (Figure 3I). Thus, the HPV8 E6 alone was sufficient to cause HPV8-induced Lrig1^+^ JZSC proliferation and expansion.

### HPV8 E6 induces Lrig1^+^ JZSC expansion into the overlying epidermis.

**Figure 4:**
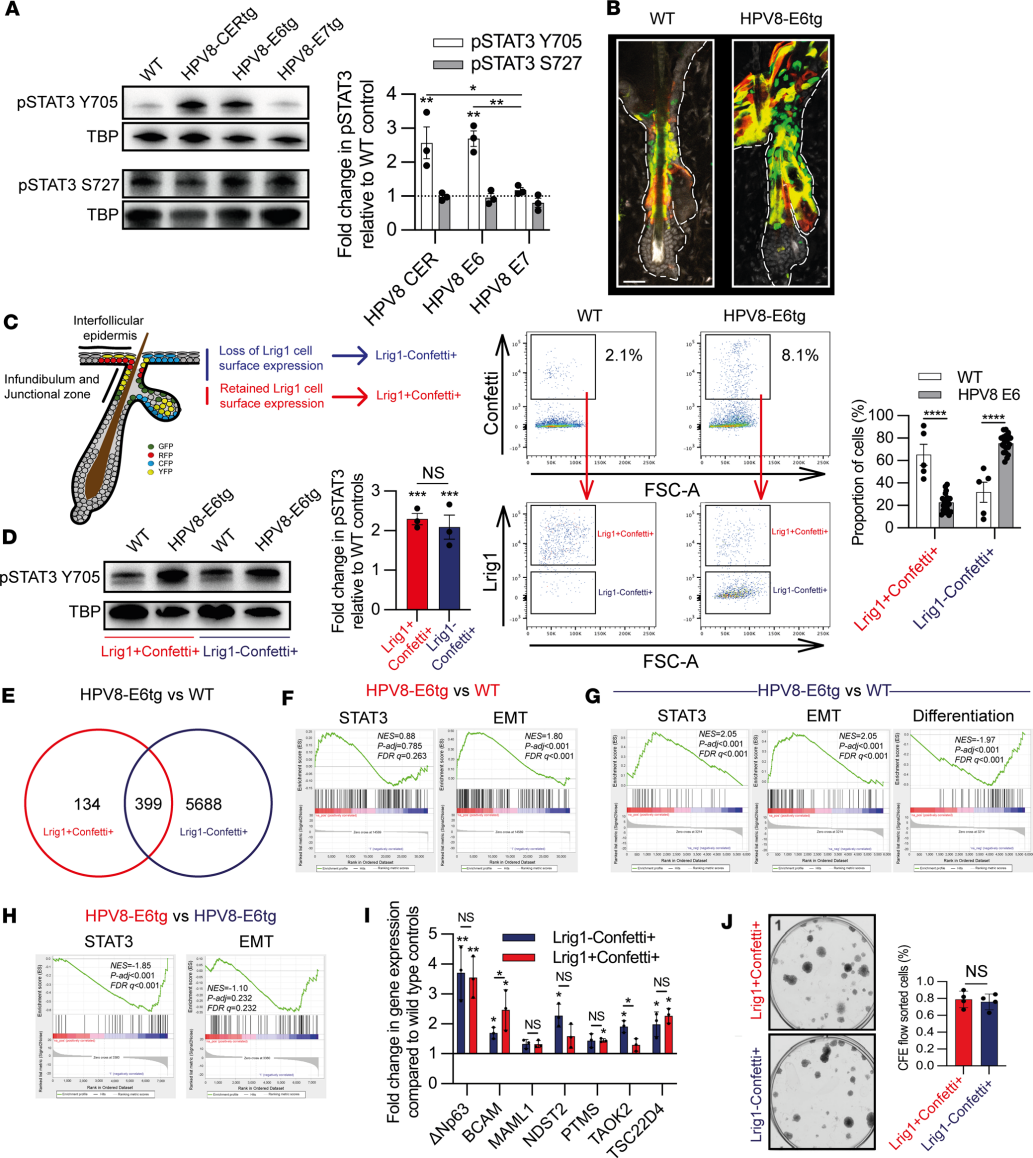
Lrig1^+^ hair follicle junctional zone KSC progeny retain KSCs. (**A**) Immunoblot of pSTAT3 Y705 and S727, with TATA-Box binding protein (TBP) control (*n* = 3). (**B**) CLSM of dorsal back skin for lineage tracing of Lrig1CreER^T2^:R26RConfetti:WT and Lrig1CreER^T2^:R26RConfetti:HPV8-E6tg progeny, 4 weeks after Cre activation. Scale bar: 40 μm**.** (**C**) Enumerated Lrig1^+^Confetti^+^ cells and their progeny Lrig1^–^Confetti^+^ flow-sorted cell populations from Lrig1CreER^T2^:R26RConfetti:HPV8-E6tg and Lrig1CreER^T2^:R26RConfetti:WT mice (*n* = 25 total). (**D**) Immunoblot of Lrig1^+^Confetti^+^ cells and their progeny Lrig1^–^Confetti^+^ flow-sorted cell populations (*n* = 3). (**E**) Venn diagram showing shared DEGs from Confetti HPV8 E6 versus Confetti WT comparisons for Lrig1^+^Confetti^+^ and Lrig1^–^ Confetti^+^ populations (see Supplemental Table 2). (**F**) GSEA for STAT3- and EMT-associated gene signatures in DEGs from Lrig1^+^Confetti^+^ transcriptomic comparison of Confetti HPV8 E6 versus Confetti WT analysis. (**G**) GSEA for STAT3-, EMT- and differentiation-associated gene signatures in DEGs from Lrig1^–^Confetti^+^ transcriptomic comparison of Confetti HPV8 E6 versus Confetti WT analysis. (**H**) GSEA for STAT3- and EMT-associated gene signatures in DEGs from Confetti HPV8 E6 transcriptomic comparison of Lrig1^+^Confetti^+^ and Lrig1^–^Confetti^+^ population analysis. See also Supplemental Figure 3. (**I**) qPCR of RNA from flow-sorted cell isolates as in **C** for STAT3-regulated genes (*n* = 3). (**J**) CFE of 2,500 flow-sorted Lrig1^+^Confetti^+^ and Lrig1^–^Confetti^+^ flow-sorted cell populations from Lrig1CreER^T2^:R26RConfetti:HPV8-E6tg (*n* = 4). See also Supplemental Figure 3. Statistical tests included (**A**, **D**, and **I**) 1-way ANOVA and (**C** and **J**) 2-tailed Student’s *t* test. *P < 0.05; **P < 0.01; ***P < 0.001; ****P < 0.0001.

HPV8-E6tg mouse skin nuclear fractions demonstrated 2.7-fold greater Tyr705 STAT3 phosphorylation but no change in Ser727 STAT3 phosphorylation (Figure 4A). Next, we sought to determine whether HPV8 E6–driven Lrig1^+^ JZSC proliferation was sufficient to cause expansion into the overlying epidermis by lineage tracing. We crossed Lrig1CreER^T2^:R26RConfetti and Krt15CrePGR:R26RConfetti mice with HPV8-E6tg mice to generate Lrig1CreER^T2^:R26RConfetti:HPV8-E6tg and Krt15CrePGR:R26RConfetti:HPV8-E6tg mice, wherein recombination occurred in nearly all cells (98%; data not shown). As before, Lrig1CreER^T2^:R26RConfetti:HPV8-E6tg mice demonstrated epidermal fluorescence, consistent with Lrig1^+^ JZSC proliferation and expansion into the overlying epidermis (Figure 4B). Confetti-labeled cells could be identified by flow cytometry in the GFP channel using the 488nm laser, enabling us to simultaneously identify Lrig1^+^ cells by antibody labeling, such that we could isolate flow-sorted Lrig1^+^Confetti^+^ cells and their Lrig1^–^Confetti^+^ progeny from Lrig1CreER^T2^:R26RConfetti:WT and Lrig1CreER^T2^:R26RConfetti:HPV8-E6tg cells (Figure 4C). As expected there were more Lrig1^–^Confetti^+^ progeny in the Lrig1CreER^T2^:R26RConfetti:HPV8-E6tg mice compared with Lrig1CreER^T2^:R26RConfetti:WT mice. WB analysis of nuclear fractions demonstrated 2-fold greater Tyr705 STAT3 phosphorylation in Lrig1^+^Confetti^+^ and Lrig1^–^Confetti^+^ cells from Lrig1CreER^T2^:R26RConfetti:HPV8-E6tg mice compared with Lrig1CreER^T2^:R26RConfetti:WT mice (Figure 4D). RNA-Seq of flow-sorted populations showed that a relatively small number of DEGs (533 DEGs) differentiated Lrig1^+^Confetti^+^ cells from Lrig1CreER^T2^:R26RConfetti:HPV8-E6tg and Lrig1CreER^T2^:R26RConfetti:WT mice (Figure 4E, Supplemental Figure 3A, and Supplemental Tables 2 and 3), but that was more than twice as many (212 DEGs) as in the reciprocal comparison between HPV8-CERtg and WT mice (Figure 2A), suggesting that the other early region genes may mitigate against the effect of E6. Consistent with the expansion and migration of Lrig1^+^ JZSCs into the overlying epidermis, GSEA identified STAT3, EMT (Figure 4F), and stem cell proliferation but not differentiation or MYC (Supplemental Figure 3B) gene signatures in the comparison of the Lrig1^+^Confetti^+^ populations. A larger number of DEGs (6,087 DEGs) distinguished the Lrig1^–^Confetti^+^ populations, wherein GSEA similarly identified STAT3 and EMT but not stem cell differentiation (Figure 4G), as well as stem cell and migration (Supplemental Figure 3C) gene signatures in the HPV-E6tg population. Surprisingly, GSEA of HPV8-E6tg Lrig1^+^Confetti^+^ versus Lrig1^–^Confetti^+^ cells identified negative enrichment for STAT3 but no difference in EMT gene signatures, suggesting that STAT3 transcriptional node activation was still evident within the Lrig1 progeny (Figure 4H). Consistent with STAT3 activation in the Lrig1^+^ JZSCs and their progeny in HPV8-E6tg mice, there was a large overlap in DEGs when these populations are compared with WT mice (Supplemental Figure 3D). Comparison of HPV8-E6tg Lrig1^+^Confetti^+^ and Lrig1^–^Confetti^+^ cells demonstrated similar expression levels of STAT3-regulated genes compared with their WT counterparts (Figure 4I). Consistent with retained KSCs within the HPV8-E6tg Lrig1^+^ JZSC progeny, we determined no difference in CFE between HPV8-E6tg Lrig1^+^Confetti^+^ and Lrig1^–^Confetti^+^ keratinocytes (Figure 4J). Hence, HPV8-E6 induced selective proliferation of Lrig1^+^ JZSCs that led to expansion of KSCs into the overlying infundibulum and epidermis.

### E6 bound p300 activates the STAT3 regulatory node.

**Figure 5:**
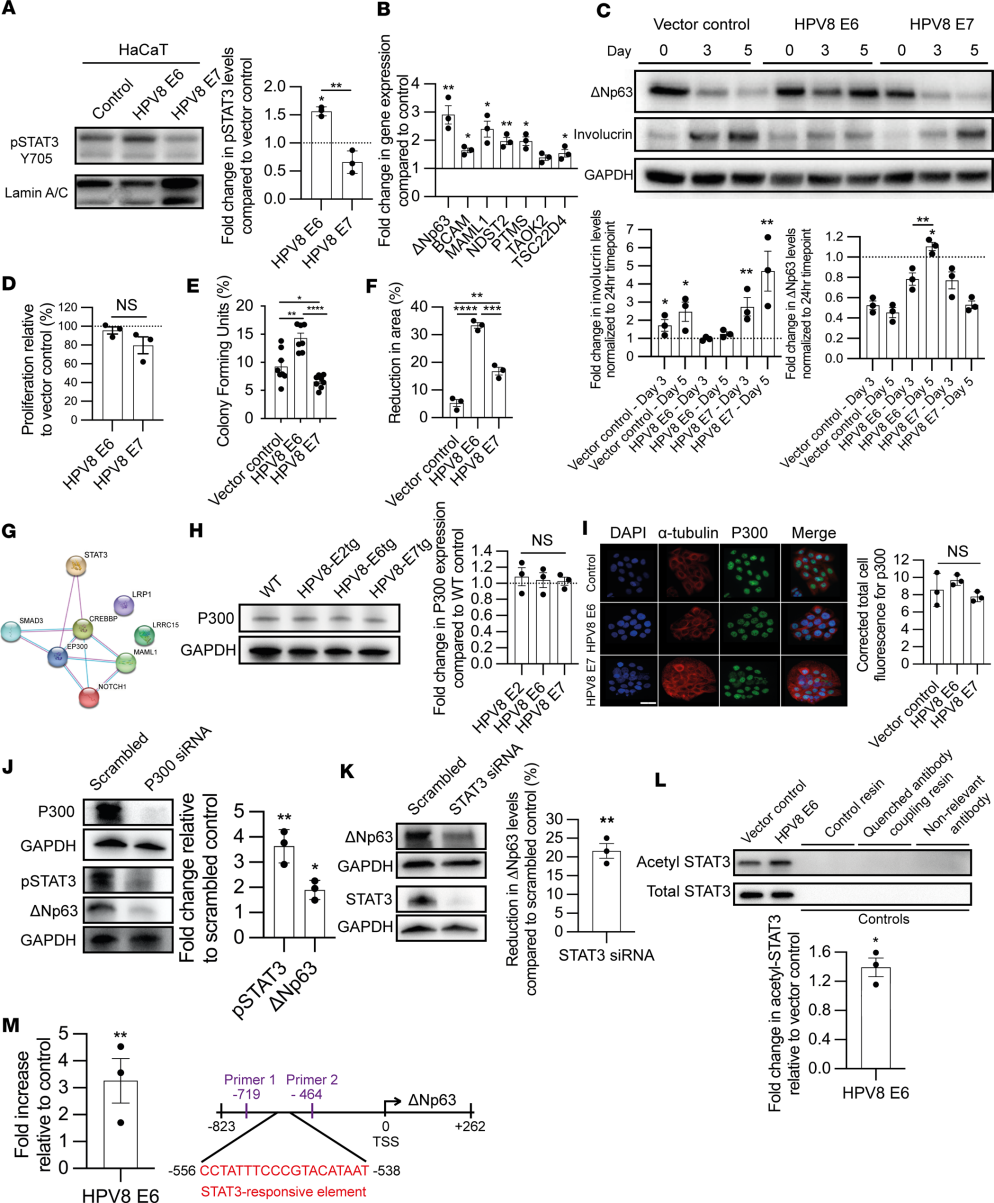
HPV8 E6 p300 interaction activates STAT3. (**A**) pSTAT3 Y705 immunoblot, with laminin A/C control, of nuclear extracts from transduced HaCaT keratinocytes (*n* = 3). (**B**) qPCR of RNA from transduced HaCaT keratinocytes for STAT3-regulated genes, with β-actin as control (*n* = 3). (**C**) Immunoblot of ΔNp63 and involucrin, with GAPDH as control, of transduced HaCaT keratinocytes cultured in high-calcium (60 μm) media for 3 and 5 days (*n* = 3). (**D**–**F**) Proliferation (*n* = 3) (**D**), CFE assay (*n* = 7) (**E**), and migration (*n* = 3) (**F**) of transduced HaCaT keratinocytes. (**G**) String analysis demonstrating the interaction of known HPV8 E6 protein binding partners and STAT3. Line colors define interactions as experimentally determined (pink) or from curated database (blue). (**H**) p300 with GAPDH control immunoblot of WT and HPV8-E2tg, -E6tg and -E7tg mouse keratinocytes (*n* = 3/genotype). (**I**) p300, α-tubulin, and DAPI immunofluorescence labeling of transduced HaCaT keratinocytes (*n* = 3). Scale bar: 40mm. (**J**) p300, pSTAT3 Y705, and ΔNp63 immunoblot, with GAPDH endogenous control, of HPV8 E6–transduced HaCaT keratinocytes treated with scrambled control and p300 targeting siRNA (*n* = 3). (**K**) STAT3 and ΔNp63 immunoblot, with GAPDH control, of HPV8 E6–transduced HaCaT keratinocytes treated with scrambled control and STAT3 targeting siRNA (*n* = 3). (**L**) Immunoblot of STAT3 immunoprecipitated nuclear protein from vector and HPV8 E6–transduced HaCaT keratinocytes probed for acetylated STAT3 and total STAT3 (*n* = 3). (**M**) qPCR analysis of ΔNp63 primers on STAT3 chromatin immunoprecipitants in HPV8 E6–transduced HaCaT keratinocytes relative to vector (*n* = 3). Schematic of the 5′-flanking region indicating primers sequences relative to STAT3-RE and ΔNp63 TSS. See also Supplemental Figure 4. Statistical tests included (**A**, **C**–**F**, **H**, and **I**) 1-way ANOVA and (**B** and **J**–**M**) 2-tailed Student’s *t* test. *P < 0.05; **P < 0.01; ***P < 0.001; ****P < 0.0001.

To determine how the HPV8 E6 protein might activate the STAT3 regulatory node, we used the previously reported stably transduced human keratinocytes, HaCaT (Figure 5) and PM1 (Supplemental Figure 4) (50, 51). Consistent with our findings in the HPV8-CERtg and HPV8-E6tg mouse keratinocytes, but in contrast to vector control– and E7-, the E6-transduced HaCaT cells demonstrated higher levels of pSTAT3 Y705 when comparing nuclear protein fractions (Figure 5A). Likewise, E6-transduced HaCaT cells, but not E7-transduced HaCaT cells, exhibited increased expression of STAT3 downstream target genes, including Δ*Np63* (Figure 5B and Supplemental Figure 4A). When cultured keratinocytes were subjected to higher calcium concentrations to simulate epidermal differentiation, E6-transduced cells maintained ΔNp63 expression and exhibited delayed expression of the differentiation marker involucrin, compared with vector control– and E7-transduced cells (Figure 5C). There was no difference in proliferation (Figure 5D and Supplemental Figure 4B); however, HPV8 E6–transduced keratinocytes demonstrated 1.5-fold greater CFE when compared with vector control– and E7-transduced keratinocytes (Figure 5E and Supplemental Figure 4C). Consistent with the egress of HPV8 Lrig1^+^ JZSCs, E6-transduced human keratinocytes migrated significantly faster when compared with vector only– and E7-transduced cells (Figure 5F and Supplemental Figure 4D). Therefore, E6-transduced human keratinocytes demonstrated activation of the STAT3 regulatory node, which regulates the expression of Δ*Np63*, increasing KSC and migratory potential.

Across several HPV genotypes E6 binding partners have been identified using immunoprecipitation and mass spectroscopy, of which HPV8 E6 bound 7 proteins: EP300 (p300), CREB binding protein, SMAD3, LRP1, LRRC15, MAML1, and NOTCH1 (52). String analysis identified two related histone acetyltransferase proteins that regulate transcription via chromatin remodeling and also acetylate STAT3, thereby enhancing its transcriptional activity: p300 (combined score: 0.986) and CREB binding protein (combined score: 0.967) (53–60) (Figure 5G and Supplemental Figure 4). HPV8 E6 has a relatively unique 132– to 136–amino acid sequence that directly facilitates binding to the ubiquitously expressed related paralog transcriptional coactivators p300 and CREB binding protein (61). A single amino acid substitution, HPV8 E6 K136N, could block p300 binding, and in transgenic mice, expressing this mutant E6 prevent papilloma formation after UVB exposure (62). Here, we show that HPV8 E6 K136N mutation did not induce STAT3 Y705 phosphorylation (Supplemental Figure 4E) or increase ΔNp63 expression (Supplemental Figure 4F). Furthermore, mice expressing HPV8 E6 K136N demonstrated normal levels of Tyr705 STAT3 phosphorylation compared with native HPV8 E6 (Supplemental Figure 4G). p300 was ubiquitously expressed in HPV8 mouse keratinocytes (Figure 5H) and transduced human keratinocytes (Figure 5I and Supplemental Figure 4H). Knockdown of p300 expression by siRNA decreased STAT3 Y705 phosphorylation and ΔNp63 expression (Figure 5J and Supplemental Figure 4K). Likewise, knockdown of STAT3 also led to a reduction in ΔNp63 expression (Figure 5K and Supplemental Figure 4L). Total STAT3 immunoprecipitation of nuclear extracts showed a greater amount of acetylated STAT3 in E6 compared with vector control (Figure 5L). Consistent with these findings, STAT3 ChIP confirmed enriched binding of endogenous STAT3 to the putative STAT3-responsive element within the 5′-flanking region ΔNp63 promoter in E6-transduced cells relative to vector control cells (Figure 5M). Hence, HPV8 E6 binding to p300 is necessary for activation of the STAT3 regulatory node.

### YAP contributes to STAT3-regulated ΔNp63 expression.

**Figure 6:**
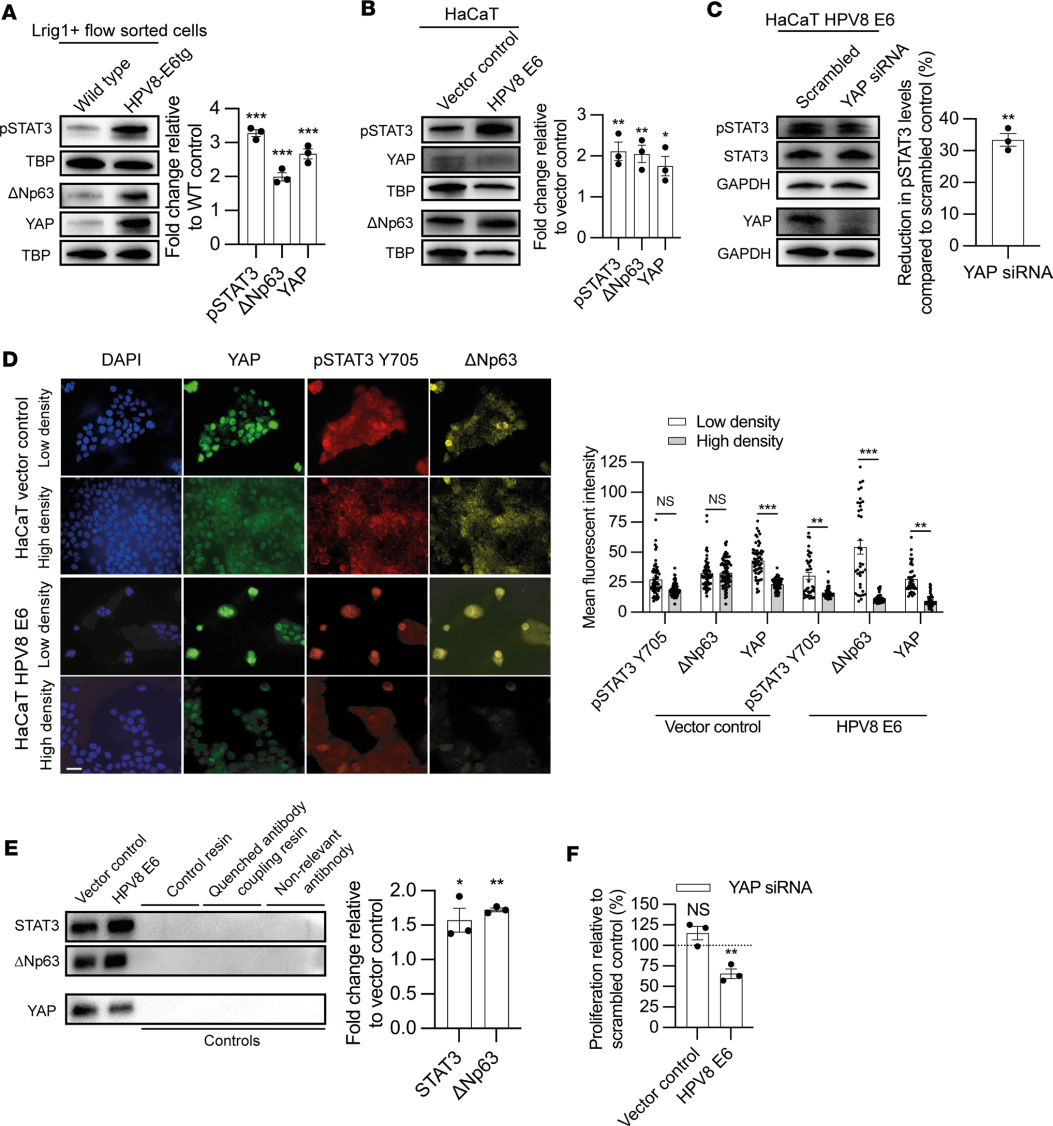
YAP a cotranscription factor for STAT3 and Δ**Np63.** (**A** and **B**) pSTAT3 Y705, ΔNp63, and YAP immunoblot, with TATA-box binding protein endogenous control, of nuclear extracts from Lrig1^+^ flow-sorted WT and HPV8-E6tg mouse keratinocytes (**A**) and HPV8 E6– and vector control–transduced HaCaT keratinocytes (**B**) (*n* = 3 per genotype/cell line). (**C**) pSTAT3 Y705, total STAT3 and YAP immunoblot, with GAPDH endogenous loading control, of HPV8 E6–transduced HaCaT keratinocytes treated with scrambled control and YAP targeting siRNA (*n* = 3). (**D**) Immunofluorescence labeling of HPV8 E6– and vector control–transduced HaCaT keratinocytes cultured at low (~50%) and high (~90%) confluency for YAP (green), pSTAT3 Y705 (red), and ΔNp63 (yellow), with quantification of nuclear mean fluorescent intensity (*n* = 82 cells total quantified over 3 independent experiments). Scale bar: 40 μm. (**E**) Immunoblot of YAP immunoprecipitated nuclear protein from vector control– and HPV8 E6–transduced HaCaT cells probed for STAT3 and ΔNp63 (*n* = 3). (**F**) Proliferation of HPV8 E6– and vector control–transduced HaCaT keratinocytes assessed by 24 hours of BrdU incorporation following treatment with YAP targeting siRNA (*n* = 3). See also Supplemental Figure 5. Statistical tests included (**A**–**E**) 2-tailed Student’s *t* test and (**F**) 1-way ANOVA. *P < 0.05; **P < 0.01; ***P < 0.001.

KSCs demonstrate nuclear YAP translocation, as diminished Hippo signaling leads to unphosphorylated YAP translocating into the nucleus where it can interact with STAT3 to participate in transcription (63). To determine if nuclear YAP was essential for HPV8 E6–induced Lrig1^+^ JZSC proliferation, we first determined that nuclear YAP was increased in nuclear protein extracts from Lrig1^+^ flow-sorted HPV8-E6tg versus WT mouse skin and the E6-transduced HaCaT cell line by Western blot (Figure 6, A and B, respectively). YAP siRNA knockdown in E6-transduced HaCaT cells, but not vector control cells, led to a reduction in Tyr705 STAT3 phosphorylation (Figure 6C and Supplemental Figure 5, respectively). As nuclear translocation of YAP has previously been reported when cells are cultured sparsely (63), we similarly observed nuclear YAP within sparsely cultured E6-transduced HaCaT cells, relative to vector control, wherein it colocalized with Tyr705 STAT3 phosphorylation and ΔNp63 expression (Figure 6D). Furthermore, immunoprecipitation of YAP nuclear protein extracts from E6-transduced HaCaT cells grown at approximately 50% confluence demonstrated higher levels of bound STAT3 and ΔNp63 compared with vector control, suggesting that YAP may be a cotranscription factor for both STAT3 and ΔNp63 (Figure 6E). Knockdown of YAP reduced HPV8 E6–induced cell proliferation (Figure 6F). Hence, HPV8-E6tg KSC proliferation was dependent upon YAP as an essential cofactor for STAT3 and ΔNp63 transcription.

### HPV8 is associated with human actinic keratoses.

**Figure 7:**
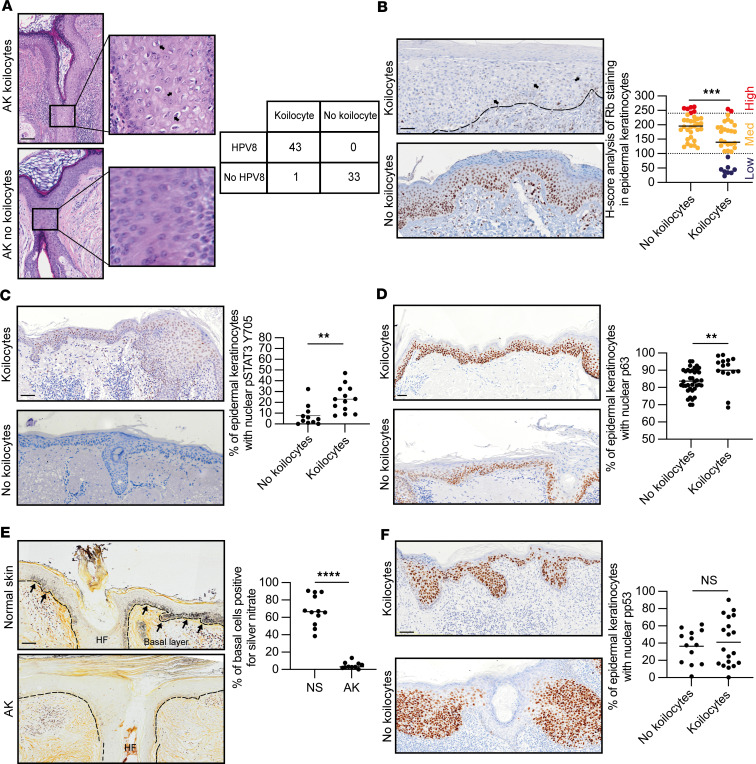
HPV8 reactivation in actinic keratosis with koilocytes. (**A**) (Left) H&E-stained human AK sections with and without koilocytes. (Right) Presence of koilocytes by HPV8 detection using β-HPV L1 open reading frame PCR-reverse hybridization assay. See also Supplemental Figure 6A. Arrows indicate the presence of koilocytes. Scale bar: 50 μm. Original magnification, ×40 (high-magnification images). (**B**–**D**) IHC of human AK tissue for Rb (*n* = 64), pSTAT3 Y705 (*n* = 24) and p63 (*n* = 53). Arrows indicate the presence of koilocytes. Scale bar: 50 μm. (**E**) Warthin-Starry stain of human AK tissue (*n* = 24). Arrows indicate the presence of koilocytes. Scale bar: 50 μm. (**F**) IHC of human AK tissue for p53 (*n* = 32). Scale bar: 50 μm. See also Supplemental Figure 6. Statistical tests included (**B**–**F**). 2-tailed Student’s *t* test. **P < 0.01; ***P < 0.001; ****P < 0.0001.

HPV8 has been linked with keratoses and cSCC in patients with the primary immunodeficiency syndrome EV, wherein koilocytes are present, and in other forms of immunosuppression. Thus, we hypothesized that the presence of koilocytes in AK may indicate HPV8 reactivation within the general population from immunosuppression associated with aging and/or sun exposure. Of 275 patients with pathologist-defined AK, we determined the presence of koilocytes in H&E-stained tissue samples in 193 (70%) (Figure 7A). Using a representative subset of 77 cases (44 with koilocytes), we determined that the presence of koilocytes in AK was not associated with significant differences in age, sex (Fisher’s exact test, NS), body location, or histological classifications (Supplemental Table 4). To determine the presence of HPV8, we used a β-HPV L1 open reading frame PCR-reverse hybridization assay (detects 25 β-HPV types), DNA analysis by nested PCR, and tissue immunofluorescence for HPV8 E4 protein (Supplemental Figure 6A). The PCR-reverse hybridization assay, which has been reported to be the most sensitive assay (64), identified 6 of 43 HPV8^+^ samples with a high yield (>100 DNA copies per cell) and 37 of 43 HPV8^+^ samples with low yield (no HPV47 was detected). The presence of koilocytes within AK was 100% sensitive for HPV8 with a 98% positive predictive value. In the remaining koilocyte AK case, HPV38 was detected, whereas in the absence of koilocytes in AK no β-HPV types were detected (Figure 7A). Thus, AK koilocytes predicted the detection of HPV8. Consistent with HPV8 E7-mediated ubiquitination and proteasomal degradation (Supplemental Figure 6B), AK samples with koilocytes had lower levels of Rb1 protein (Figure 7B), even though p16 expression showed no difference (Supplemental Figure 6C). We observed a much greater frequency of nuclear pSTAT3 Y705 labeling within the epidermis of koilocyte-containing AK than without, 23.10% ± 3.38% versus 7.68% ± 2.92%, respectively (Figure 7C). In normal skin, p63 antibody labeling identified basal cells, but in AK, suprabasal p63 labeling was observed in the HF infundibulum and adjoining epidermis (Figure 7D). The frequency of p63 labeling was marginally greater among AK with koilocytes (Figure 7D). Although the detection of HPV8 DNA does not necessarily infer viral reactivation, the presence of koilocytes in AK associated with reduction in Rb1 is highly suggestive.

Human epidermal keratinocytes are protected from UV-induced DNA damage by melanosomes transferred from adjacent melanocytes, which form a “melanin” cap over the nucleus. We therefore hypothesized that the constant proliferation and translocation of JZSCs into the adjoining epidermis may lessen melanosome protection. Compared with normal skin, melanin staining was absent in all AK, irrespective of the presence of koilocytes (Figure 7E). Thus, since the absence of melanosomes is common, next we studied the DNA damage response to DNA double-strand breaks, which involves phosphorylation of the histone variant H2AX at serine 139 in the flanking regions of chromatin and can be labeled with specific antibodies that form visible foci in mammalian cells (65). The percentage of nuclei with phosphorylated H2AX labeling was much greater in AK with koilocytes than that without (92.43% ± 3.97% vs. 53.86% ± 8.40%, respectively, Supplemental Figure 6D). UV frequently mutates p53 in human AK; as expected, there was no difference in the frequency of nuclear p53 in AK, with (41.10% ± 6.36%) and without (36.46% ± 5.34%) koilocytes in the epidermis (Figure 7F); similarly, there was no difference in p21 labeling (Supplemental Figure 6E). Therefore, based on the HPV8-CERtg mouse model, in human AK with koilocytes, HPV8 may also activate pSTAT3 to drive p63 expression leading to HF junctional zone expansion and displacement without melanin protection into the overlying UV-exposed epidermis (Supplemental Figure 6F).

## Discussion

The mammalian skin contains several adult tissue stem cell populations, wherein the Lrig1^+^ JZSC represents a transcriptionally distinct population (45, 66). Although Lrig1 expression itself has been used to identify a number of adult stem cell populations in different tissues (46, 67–71). Lrig1 is a negative regulator of EGFR signaling and therefore promotes stem cell quiescence by binding to EGFR, causing its ubiquitination and proteasomal degradation (72, 73). Furthermore, EGFR signaling activates the c-Myc transcriptional node, resulting in Lrig1 expression, such that Lrig1 expression represents an autoregulatory negative feedback loop (46, 72, 74). Loss of Lrig1 expression in the skin leads to autonomous JZSC proliferation with increased cell numbers in the overlying HF infundibulum and perifollicular epidermis (46, 75). During homeostasis, as shown in our Lrig1CreER^T2^:R26RConfetti model, the Lrig1^+^ JZSC population contributes to the maintenance of cell numbers in the sebaceous gland and infundibulum (68, 76, 77). In keeping with this, it has also been proposed that Lrig1 JZSC transformation is the basis for sebaceous carcinoma (78). Thus, during homeostasis, the Lrig1^+^ JZSCs represent a tightly regulated distinct functional HF population.

Here, we have shown that HPV8 early region genes, which are shared by other HPVs across the genera, specifically circumvent the Lrig1^+^ JZSC c-Myc regulatory node to induce proliferation and KSC expansion into the interfollicular epidermis. The c-Myc transcriptional node clearly distinguishes the Lrig1^+^ JZSCs from the CD34^+^ HF bulge KSC population, even in the context of HPV8 early region gene expression. Herein we describe an alternative pathway for Lrig1^+^ JZSC proliferation governed by the STAT3 regulatory node signaling through downstream target genes, which include *c-Myc* and Δ*Np63*. This pathway, which is only activated in the Lrig1 JZSC population, is driven by HPV8 E6 protein interaction with p300, which activates STAT3. Importantly, we show that STAT3 activation causes the proliferation and expansion of KSCs through increased symmetric cell division, allowing KSCs to be displaced from their niche into the interfollicular epidermis. Moreover, it appears that Lrig1 is an important but pleiotropic factor that inhibits STAT3 and multiple other growth factor receptors from signaling, including c-Met (79), RET (80), neurotrophic receptor tyrosine kinase 2 (TrkB, NRTK2) (81), TNF-α (82), and androgen receptors (83). Corneal wounding, in the absence of Lrig1, led to STAT3 activation and premature corneal opacification (84). Whether STAT3 regulatory node activation in the Lrig1 JZSC population is responsible for the transient transfer of KSCs into the interfollicular epidermis after injury, since epidermal loss of STAT3 is associated with delayed wound healing, remains to be determined (85–87). In the context of HPV, this mechanism may allow the virus to reside in a protected JZSC population and upon reactivation still release virions via the overlying interfollicular epidermis.

STAT3 activation, which is associated with cytokine signaling in immune cells, has been observed in several malignancies, both within cancer cells, but also, within the tumor microenvironment immune cells (88). Numerous oncogenic signaling pathways converge to give rise to constitutive STAT3 activation, although less frequent STAT3 oncogenic mutations occur in myeloproliferative and skin malignancies (89). Inhibitors of the IL-6/JAK/STAT3 pathway are already in clinical use, and novel STAT3 selective inhibitors are currently in development. Constitutive activation of STATs, in particular STAT3, is found in carcinoma from the head and neck (90), lung (91), breast (92), ovary (93), and prostate (94). Within the context of cSCC, STAT3 deficiency is sufficient to block tumor formation in the 2-step chemical skin cancer mouse model, wherein tumors develop from within the HF bulge KSC population (95). Although not described in the context of the Lrig1 JZSC population, constitutive expression of activated STAT3 in the skin leads to keratinocyte proliferation and expansion, similar to what we have observed in the Lrig1CreER^T2^:R26RConfetti:HPV8-CERtg mice but not in the Krt15CrePGR:R26RConfetti:HPV8-CERtg mice, with increased susceptibility for UV-induced transformation (96). Consistent with the importance of STAT3 signaling in HPV8, HPV8-CER:STAT3^+/–^tg mice did not demonstrate Lrig1^+^ JZSC expansion or tumorigenesis.

HPV8 E6 exhibits intrinsic oncogenic activity (43), but unlike α HPV it does not bind and inactivate p53 by rapid proteasome-mediated degradation, although it may prevent its stabilization (97, 98). Multiple studies have demonstrated the ability of HPV8 to bind p300 (52, 61, 62, 99, 100). The ability of HPV8 E6 to transform keratinocytes has been studied for other binding partners affecting tumor suppressor and oncogenic pathways: Notch (101–103), TGF-β (104), Hippo (105), EGFR (106), and Wnt (107). In addition to the ability of HPV8 E6–bound p300 to activate the STAT3 pathway, as described herein, the association has been shown to attenuate activation of 2 essential DNA repair kinases, ATM and ATR (108). We and others have shown HPV8-associated impaired DNA repair, which would facilitate the acquisition of transforming mutations (40, 109, 110).

HPV8 reactivation–associated JZSC proliferation and expansion mirrors the pathological findings in human AK (38). While the presence of koilocytes in AK has been reported, their presence has previously been attributed to UV-induced transformation. Here, we show that the presence of koilocytes in AK is indicative of HPV8, with loss of Rb1 and increased STAT3 phosphorylation. Although HPV8 E7 demonstrated lower binding of Rb1 and does not directly cause degradation, we have previously shown reduced Rb1 levels in human keratinocytes expressing E7 and all the complete early region genes (111–113). The archetypal AK pathology findings include a dilated HF infundibulum with overlying orthokeratosis and an accumulation of atypical keratinocytes within the perifollicular epidermis (114). As would be expected from viral reactivation, AKs are frequently observed with a dense inflammatory cell infiltrate. While in our mouse models constitutive expression of the HPV8 early region genes results in JZSC proliferation and expansion into the overlying interfollicular epidermis, the ensuing immune response is able to restore equilibrium in native infection. This explains the increased risk of AK in immune-suppressed individuals and similarly why in otherwise healthy individuals, 87% of AK spontaneously resolve within 4 years (115). Herein, we hypothesize that JZSC proliferation and expansion into the overlying interfollicular epidermis occurs in the absence of melanin protection, such that these keratinocytes easily accrue UV-induced mutations; thus, providing a basis for the hit-and-run mechanism. Consistent with this, we observed p21 and p53 clones throughout the AK epidermis and H2AX phosphorylation. In conclusion, our findings in the context of HPV8 reactivation redefine human AK as a HF disorder of KSCs and provide a mechanistic explanation for the hit-and-run hypothesis for HPV8-induced cSCC.

## Methods

Further information can be found in Supplemental Methods.

### Sex as a biological variable

For both human and animal models in this study, male and female samples were used, and similar findings were reported for both sexes.

### Experimental models

#### Mice.

B6.129P2-*Gt(ROSA)26Sor^tm1(CAG–Brainbow2.1)Cle^*/J (44, 116), *Lrig1^tm1.1(cre/ERT2)Rjc^*/J (117), and B6;SJL-Tg(Krt1-15-cre/PGR*)22Cot/J (118) mice were purchased from The Jackson Laboratory. Lrig1-EGFP-ires-CreER^T2^ mice were a gift from Kim Jensen (University of Copenhagen, Copenhagen, Denmark) (46). Krt14-HPV8-CER (39), Krt14-HPV8-E2 (42), Krt14-HPV8-E6 (43), and Krt14-HPV8-E7 (41) mice were used in this study. B6.129P2-*Gt(ROSA)26Sor^tm1(CAG–Brainbow2.1)Cle^*/J, *Lrig1^tm1.1(cre/ERT2)Rjc^*/J, B6;SJL-Tg(Krt1-15-cre/PGR*)22Cot/J, and Lrig1-EGFP-ires-CreER^T2^ mice were backcrossed with FVBN mice (The Jackson Laboratory) for 6 generations to yield a pure FVBN background and finally interbred with Krt14-HPV8-CER and Krt14-HPV8-E6 mice. Stat3^WT/LoxP^/FVBN and Stat3^WT/WT^/FVBN mice were crossed with Krt14-HPV8-CER mice to generate Stat3^WT/LoxP^/Krt14-HPV8/FVBN and Stat3^WT/WT^/Krt14-HPV8/FVBN mice.

#### Tamoxifen and RU486 injection.

Cre activation in Lrig1CreER^T2^ mice was induced by injecting 4-week-old mice intraperitoneally with 80 mg/kg/day of tamoxifen in corn oil for 4 consecutive days. Cre activation in Krt15CrePGR mice was induced by injecting 4-week-old mice intraperitoneally with 80 mg/kg/day of RU486 in corn oil for 4 consecutive days. Mice were harvested 30 days after induction.

#### Cell lines.

Three established cell lines; HaCaT, PM1 and J2-3T3 were used in this research. Details on cell culture conditions used can be found in the Supplemental Methods.

### Generation of transduced HaCaT and PM1 cell lines

The Moloney murine leukemia retrovirus vector pLXSN (vector control) was used to generate recombinant retroviruses containing HPV8 genes coding for HPV8 E6 and E7. Briefly, retroviral transduction of HaCaT and PM1 cell lines was performed by seeding cells into 6 cm dishes, allowing them to adhere overnight, and then adding a mixture of retroviral supernatants with an equal volume of DMEM in the presence of 5 μg/mL hexadimethrine bromide (polybrene). Spin infection was made by centrifugation for 1 hour at 300*g*. Cells were washed with PBS and cultured for 2 days before treatment with G418 at a concentration of 500 μg/mL.

### Tissue dissociation and culture

Mouse dorsal back skin was dissociated into single cells as described previously (38).

### Murine primary colony forming assay culture

Mouse dorsal back skin was dissociated into single cells as described above. Lrig1-expressing mouse keratinocytes were isolated through flow sorting, and 2,500 cells were seeded on an irradiated J2-3T3 feeder layer per well in a 6-well plate and cultured in Rheinwald and Green media for 15 days, with media changed every 3 days. The colonies were stained with crystal violet, scanned with a GelCount machine (Oxford Optronix), and quantified using ImageJ software (NIH).

### siRNA knockdown experiments

siRNA transfections were performed 24 hours after seeding keratinocytes with siRNA (ON-Targetplus SMARTpool, Dharmacon), Lipofectamine 3000 transfection reagent (Thermo Fisher Scientific), and Opti-MEM (Thermo Fisher Scientific). siRNA concentrations were optimized individually. Cell lines were transfected with siRNA targeting STAT3 (20 ηM), p300 (30 ηM), YAP (20 ηM).

### Calcium shift experiment on established cell lines

Keratinocytes were dedifferentiated by culturing cells for 48 hours in EpiLife media (containing no calcium chloride; MEPICF500). Keratinocytes were induced to differentiate by adding Epilife media (containing 60 μm calcium final concentration) and left for the number of days stated in the figure to assess differentiation levels (Figure 5C).

### Whole-mount skin preparation and fluorescence imaging

Tail skin was cut into 0.5 cm^2^ pieces and placed overnight at 4°C in Dispase (2.5 U/mL). The epidermis was gently removed from the underlying dermis using forceps and fixed in 10% neutral buffered formalin for 90 minutes at room temperature. Tissue was washed in PBS and stored in PBS+0.2% sodium azide at 4°C ready for immunofluorescence labeling. Immunofluorescence on tail skin was performed as described previously (38). Antibodies used can be found in Supplemental Table 5.

### Immunofluorescence staining and microscopy of OCT sections

Immunofluorescence was performed on either frozen OCT–embedded or paraffin-embedded sections as previously described (38). Further experimental details and antibodies used can be found in the Supplemental Methods and Supplemental Table 5, respectively.

### IHC staining

Rehydration of sections and antigen retrieval was performed as described in the immunofluorescence staining above. Further experimental details and antibodies used can be found in the Supplemental Methods and Supplemental Table 5, respectively.

### Starry-Warthin stain

All reaction solutions were reduced from pH 4 to pH 3.2 before conducting the staining as per manufacturer’s instructions (Abcam).

### FACS and analysis

Samples were analyzed and flow sorted using BD LSR Fortessa and BD FACSAria Fusion (BD Biosciences), respectively. Mouse telogen dorsal back skin was dissociated and washed with FACS buffer (0.05% sodium azide and 0.5% BSA in PBS) before primary antibody staining for 30 minutes on ice. Primary antibodies used in this study include Lrig1 488 (R&D systems, FAB3688G), Lrig1 647 (VWR, 10330-520), and CD34 PE (BD, 551387). Unbound antibodies were removed by washing with FACS buffer twice by centrifugation. Centrifugation was performed at 250*g* for 5 minutes at 4°C. Details on the gating strategy can be found in Supplemental Methods.

### Western immunoblotting

Whole protein lysate was extracted using Lysing Matrix D tubes (MP Biomedicals) by homogenization for tissue or agitation with a pipette for cell pellets in RIPA buffer (Thermo Fisher Scientific) supplemented with 1× protease/phosphatase inhibitor cocktail (Cell Signaling). Nuclear protein lysate was extracted using NE-PER Nuclear and Cytoplasmic Extraction reagents (Thermo Fisher Scientific). Details on how Western blotting was performed and the antibodies used can be found in the Supplemental Methods and Supplemental Table 5, respectively.

### Coimmunoprecipitation

Nuclear protein lysates were prepared from HaCaT cells at a confluency of 50%–60%. Co-IP experiments were performed using the Pierce Co-Immunoprecipitation kit (Thermo Fisher Scientific). Further details on how Co-Immunoprecipitation was performed can be found in the Supplemental Methods.

### ChIP-qPCR

The ChIP-qPCR experiment was performed using the High-Sensitivity ChIP kit (Abcam) as per manufacturers guidelines. Further details on how ChIP-qPCR was performed can be found in the Supplemental Methods.

### Colony-forming ability (established cell lines)

HaCaT/PM1-PLXSN, -E6 and -E7 cells were seeded at a low density of 500 cells/well in a 6-well plate and left for 7 days in growth media to allow colonies to form. Colonies were quantified by removing the media, washing with PBS, and staining with crystal violet solution for 15 minutes on a rocker at room temperature, before washing off solution by gently running the plates under tap water. Plates were scanned and enumerated using a GelCount plate reader (Oxford Optronix).

### RNA extraction and cDNA synthesis

Depending on cell numbers, RNA was isolated using the Qiagen RNeasy Plus Mini or Micro Kits (Qiagen) per manufacturer’s instructions. The quality of the extracted RNA was assessed using the Agilent RNA 6000 Nano kit. Agilent Nano chips where run on the Agilent 2100 Bioanalyzer according to manufacturer’s guidelines. cDNA synthesis was performed using the Quantitect Reverse Transcription Kit (Qiagen) in 0.2 mL PCR tubes as per manufacturer’s instructions.

### qPCR

For qPCR gene expression studies, reactions were performed using TaqMan gene expression probes. Predesigned TaqMan primer/probes were obtained from Applied Biosystems (see Supplemental Table 5). Reactions were run using the TaqMan Universal Master Mix II (Applied Biosystems) according to the manufacturer’s guidelines. Housekeeping genes (GAPDH and β-actin) were used as reference genes. All reactions were run in 3 technical triplicates, and all experiments were performed at least 3 times independently. All reactions were run on the QuantStudio 7 Flex Real-Time PCR system (Applied Biosystems) with the QuantStudio software. Gene expression analysis of qPCR data was performed using the ΔΔCt method to calculate fold change (2^–ΔΔCt^) relative to control.

### DNA extraction, precipitation and β-HPV genotyping PCR

DNA was extracted from 25 mg of FFPE sections using the QIAamp DNA Mini Kit (Qiagen). For human samples with a DNA concentration of less than 10 ηg/μL, DNA precipitation was performed to gain a higher concentration and purity. To perform genotyping, the PM-PCR Reverse Hybridisation Assay method was performed using the RHA kit Stain (β) HPV kit (Labo Bio-Medical Products BV). Details can be found in Supplemental Methods.

### Nested PCR for HPV8 E6

The molecular detection of HPV8 in the skin tissue samples was performed using nested-PCR amplification. DNA was extracted from 44 FFPE samples exhibiting koilocytes and 33 FFPE samples with no koilocytes. Two sets of primers were designed, outer and nested. Both outer and nested sets were flanking an area in the E7–E1 region of HPV8. Details on primer sequences and PCR reaction specifications can be found in Supplemental Methods.

### RNA-Seq of mouse dorsal back skin samples

RNA for RNA-Seq was extracted, and RNA quality was assessed as mentioned previously. Upper HF (Lrig1) and bulge (CD34) stem cells were isolated by flow sorting from telogen dorsal back skin in 7-week-old Krt14-HPV8-CER and WT mice. Twelve samples (3 Lrig1^+^ WT, 3 Cd34^+^ WT, 3 Lrig1^+^ Krt14-HPV8-CER, and 3 Cd34^+^ Krt14-HPV8-CER) were sequenced (GSE248056) by Wales Gene Park. Confetti^+^ cells with and without Lrig1 cell surface expression were flow sorted from Lrig1CreER^T2^:R26RConfetti:HPV8-E6tg and Lrig1CreER^T2^:R26RConfetti:WT mice. Twelve samples (3 HPV8-E6tg Lrig1^–^Confetti^+^, 3 HPV8-E6tg Lrig1^+^Confetti^+^, 3 WT Lrig1^–^Confetti^+^, and 3 WT Lrig1^+^Confetti^+^) were sequenced (GSE248056). Total RNA was extracted using an RNeasy Micro Kit (Qiagen). RNA was then frozen at –80°C and shipped to Novogene on dry ice for library preparation and sequencing. Further details on bioinformatic analyses can be found in Supplemental Methods.

### Statistics

#### Statistics and reproducibility.

Statistical analyses were performed in GraphPad Prism v9. Data are presented as mean ± SEM. Two-tailed Student’s *t* test was used to measure significance between 2 groups, while 1-way ANOVA was used when comparing multiple groups. Specifically, statistical tests applied for each figure can be found in Supplemental Methods. *P* values of less than 0.05 are considered significant. For each experiment, *n* represents the number of experimental replicates. For animal experiments, age- and sex-matched mice were randomly assigned to groups, and at least 3 biological replicates were used for each experiment.

#### Identification of koilocyte-like keratinocytes.

Human FFPE AK tissues (*n* = 77) were serially sectioned with the first section stained with H&E and imaged. Each image was then carefully assessed for the presence of koilocytes in the epidermis. A sample was determined to be positive if multiple koilocytes were observed within 100 μm of epidermis as single entities or clusters.

#### Quantifying positively stained tissue sections.

To determine positive cell expression, images were analyzed using Qupath software (119). For pp53, p63, pSTAT3, p21, Rb1, p16, and pH2AX, the number of cells was determined using the automated cell detection tool; nuclei with either a hematoxylin or DAB optical density over the defined intensity threshold were counted, and those with a DAB value over the predetermined positive threshold value were defined as positive cells.

### Study approval

#### Animals.

All mouse experiments carried out in this study were performed with the approval of the UK Home Office (project license 30/3382).

#### Patient samples.

Human tissue samples were obtained after informed written consent was acquired from patients following NHS Research and Development and Regional Ethics Committee approval (19/NS/0012). Pathologist-diagnosed actinic keratosis samples together with anonymized clinical reports were collected. 275 patient samples were analyzed for the presence of koilocytes by histology, with a prospective cohort of the first 77 samples further studied in more detail for the presence of HPV8 via genotyping.

### Data and code availability

RNA-Seq data have been deposited at GEO (accession GSE248056). This paper does not report original code. Data are available in the Supporting Data Values file. The lead contact can provide any additional information required to reanalyze the data within this paper.

## Author contributions

GKP, BA, and MG conceived and supervised this study. GKP, HJM, CO, and BYS designed the experiments. HJM and CO performed in vitro and protein experiments. HJM, CO, and BYS performed in vivo experiments. AG, HJM, and CO performed bioinformatic analyses on RNA-Seq data sets. HJM, CO, AG, ALP, LA, and AXHL performed work on human AK samples. LM and CB performed in vivo experiments and mouse tissue processing. MDA, MH, BA, and MG contributed new reagents and expertise throughout the project; GKP and RG conducted the human study. HJM, CO, and BYS analyzed the data. GKP, HJM, and CO wrote the manuscript. All authors edited the manuscript. The order of co–first authorship was determined by effort in data analysis and drafting the manuscript.

## Supplementary Material

Supplemental data

Unedited blot and gel images

Supplemental table 1

Supplemental table 2

Supplemental table 3

Supporting data values

## Version 1 06/25/2024

In-Press Preview

## Version 2 08/08/2024

Electronic publication
