# Supplementary material for: HPV8-induced STAT3 activation led keratinocyte stem cell expansion in human actinic keratoses
Source: JCI Insight. 2024 Jun 25;9(15):e177898. doi: 10.1172/jci.insight.177898 (PMC11383611; doi:10.1172/jci.insight.177898)
Supplement: Supplemental data [file jciinsight-9-177898-s164.pdf]

# Supplemental Information

## HPV8-induced STAT3 activation led keratinocyte stem cell expansion in human actinic keratoses

Huw J Morgan,*[1] Carlotta Olivero,*[1] Boris Y Shorning,[1] Alex Gibbs,[1] Alexandra L Phillips,[1] Lokapriya Ananthan,[1] Annabelle Xiao Hui Lim,[1] Licia Martuscelli,[2] Cinzia Borgogna,[2] Marco De Andrea,[3,4] Martin Hufbauer,[5] Richard Goodwin,[6] Baki Akgül,[5] Marisa Gariglio,[2] Girish K Patel[1]

1 European Cancer Stem Cell Research Institute, School of Biosciences, Cardiff University, Maindy Road, Cardiff, UK

2 University of Piemonte Orientale, Dept. of Translational Medicine, Via Solaroli, 17 - 28100 Novara, Italy

3 Viral Pathogenesis Unit, Department of Public Health and Pediatric Sciences, University of Turin, Medical School, 10126 Turin, Italy

4 Intrinsic Immunity Unit, CAAD - Center for Translational Research on Autoimmune and Allergic Disease, University of Eastern Piedmont, 28100 Novara, Italy

5 Institute of Virology, University of Cologne, Medical Faculty and University Hospital Cologne, Cologne, Germany

6 Department of Dermatology, Aneurin Bevan University Health Board, Royal Gwent Hospital, Newport, UK

Correspondence: Girish K Patel, European Cancer Stem Cell Research Institute, Cardiff University, CF24 4HQ, Tel: (+44) 02920 88520, Email: PatelGK@Cardiff.ac.uk

* Authors contributed equally to this work

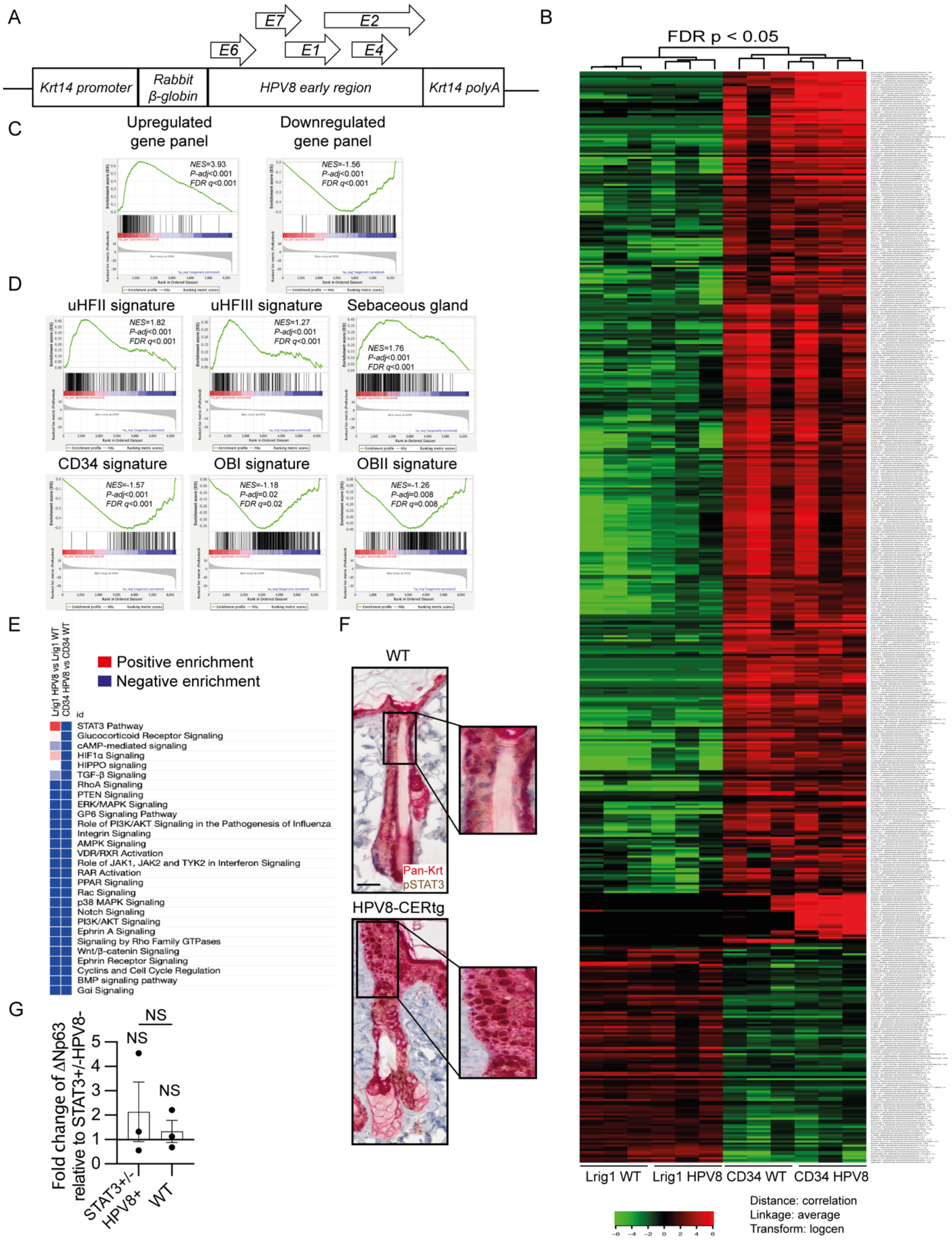


**Supplemental Figure 1. HPV8-CERtg mouse model follicle keratinocyte stem cell populations, Related to Figure 1 and 2.** (A) Schematic illustrating the genetic strategy to generate mice expressing HPV8 early region genes in skin keratinocytes. (B) Heat map of DEGs from Lrig1 vs CD34 comparisons from WT and HPV8-CERtg mice. (C) GSEA of upregulated (left) and downregulated (right) gene signatures from a previous comparison of Lrig1 and CD34 HF KSC. (D) GSEA for upper hair follicle (uHFII and uHFIII), sebaceous gland, outer layer bulge keratinocytes (OBI and OBII) and hair follicle bulge (CD34) gene signatures. (E) Ingenuity comparative analysis (IPA, Qiagen) of WT and HPV8-CERtg Lrig1 and CD34 KSC populations. (F) IHC for pSTAT3 and pan-cytokeratin on adult back skin from WT and HPV8-CERtg mice. Scale bar = 40$\mu$m. (G) QPCR of RNA from WT, STAT3$^{+/-}$, and STAT3$^{+/-}$ HPV8 epidermal keratinocytes for ΔNp63 (n=3 per genotype). Data are presented as mean $\pm$ SEM. NS, non-significant. Statistical test(s) Supplemental Figure 1F one-way ANOVA.

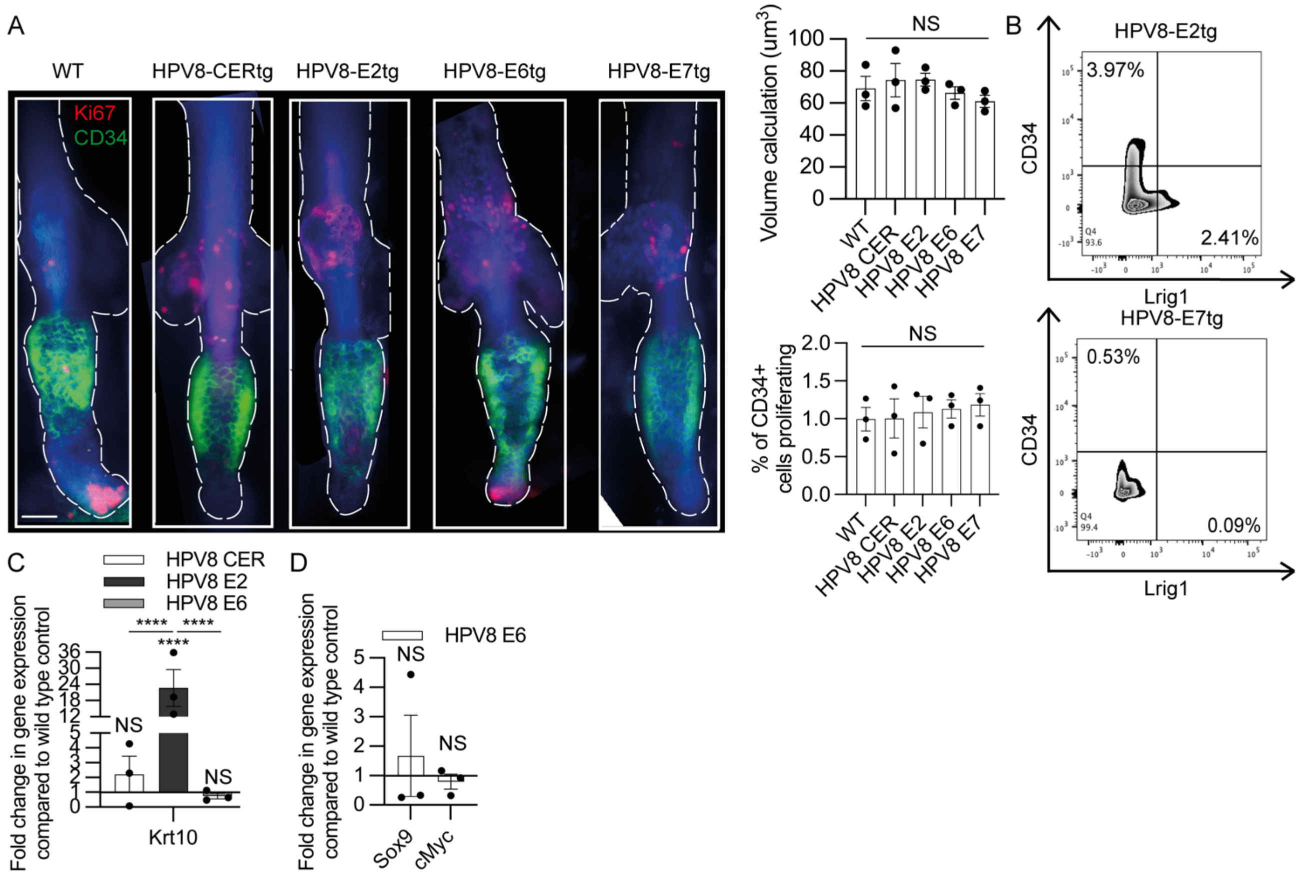


**Supplemental Figure 2. HPV8 early region genes in primary mouse keratinocytes, Related to Figure 3.** (A) CLSM of whole mount tail skins labeled for CD34, with quantification of CD34 volume and the number of co-labeled Ki67+ cells (average of 10 hair follicles per mouse). Scale bar = 40μm. Data are presented as mean ± SEM. NS, non-significant. (B) FACS for Lrig1$^+$ and CD34$^+$ populations from HPV8-E2tg and HPV8-E7tg back skin cell isolates. (C and D) QPCR of RNA from flow-sorted CD34$^+$ hair follicle KSCs from WT, HPV8-E2tg, HPV8-E6tg and HPV-CERtg mice for expression of *Krt10* (C) or *Sox9* and *c-Myc* (D) (n=3 per genotype). Data are presented as mean ± SEM. NS, non-significant. Statistical test(s) Supplemental Figure 2A and C one-way ANOVA, Supplemental Figure 2D 2-tailed Student's t-test. ****$P$<0.0001.

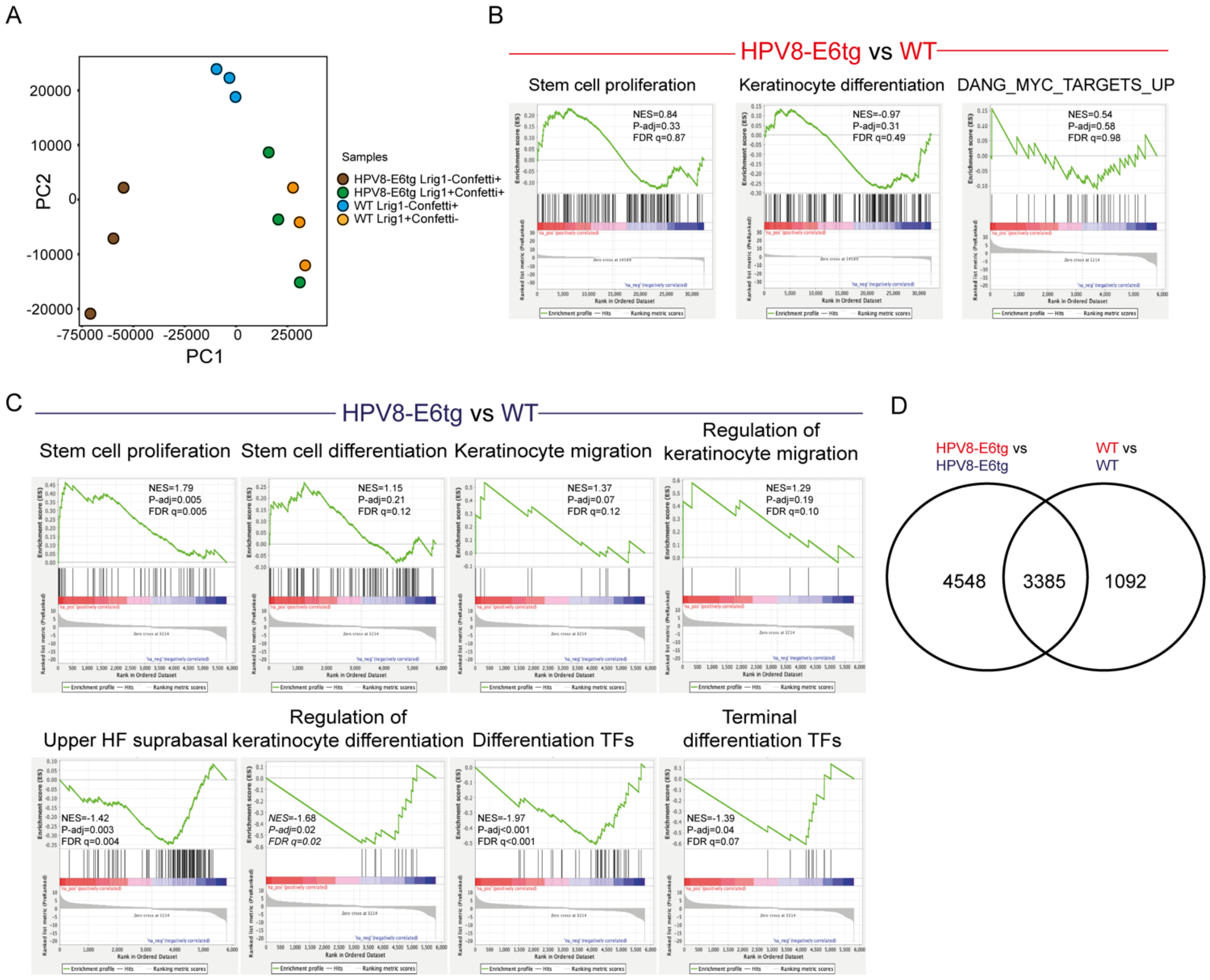


**Supplemental Figure 3. HPV8 E6tg Hair follicle keratinocyte stem cells, Related to Figure 4.** (A) PCA of RNA-seq transcriptome analysis of skin KSC populations. (B) GSEA of DEGs from transcriptomic analysis from HPV8-E6tg vs WT for the Lrig1$^{+}$Confetti$^{+}$ populations. (C) GSEA of DEGs from transcriptomic analysis from HPV8-E6tg vs WT for the Lrig1$^{-}$Confetti$^{+}$ populations. (D) Venn diagram showing shared DEGs from Lrig1$^{+}$ vs Lrig1$^{-}$ comparisons.

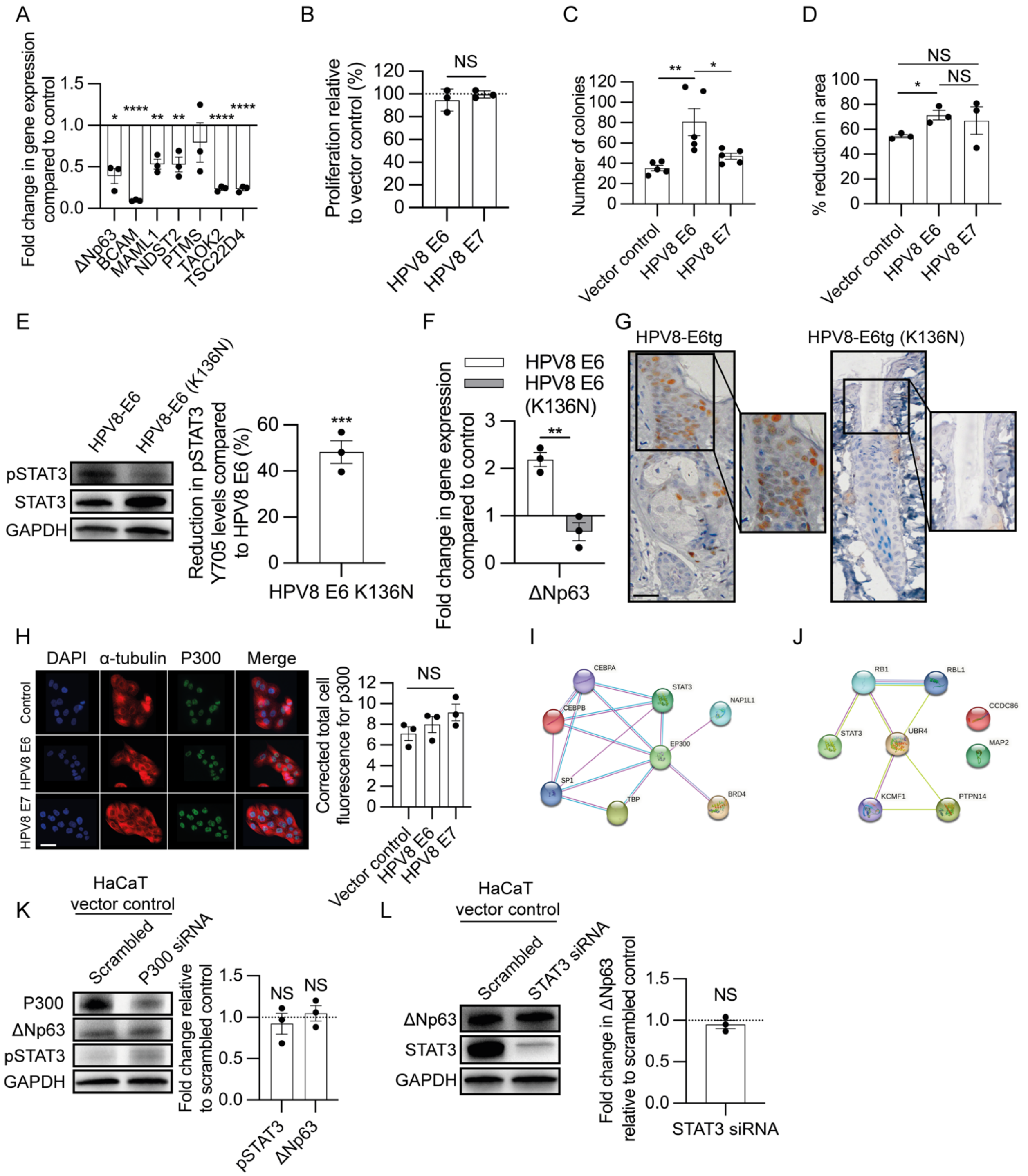


**Supplemental Figure 4. HPV8 early region genes in established human keratinocytes, Related to Figure 5.** (A) QPCR of RNA from vector control and HPV8 E7 transduced HaCaT keratinocytes for STAT3-regulated genes, with β-actin as an internal control (n=3 per cell line). (B) *In vitro* proliferation assessed by 24 hours BrdU incorporation of HPV8 E6 and E7 transduced PM1 keratinocytes compared to vector control (n=3 per cell line). Data are presented as mean ± SEM. Dotted line is the comparator. NS, non-significant. (C) CFE of 500 vector control and HPV8 E6 and E7 transduced PM1 keratinocytes per well (n=7 per cell line). **p < 0.01 by one-way ANOVA. Data are presented as mean ± SEM. (D) Cell migration assay of HPV8 E6 and E7 transduced PM1 keratinocytes assessed by percentage of closure over 24 hours compared to vector control cells (n=3 per cell line). *p < 0.05 by one-way ANOVA. Data are presented as mean ± SEM. (E) Immunoblot of pSTAT3 Y705, total STAT3 and GAPDH in HPV8 E6 and HPV8 E6 (K136N) transduced N/TERT

keratinocytes, with quantification (n=3 per genotype). (F) QPCR of RNA from HPV8 E6 and HPV8 E6 (K136N) transduced N/TERT keratinocytes for ΔNp63, with β-actin as an internal control (n=3 per cell line). (G) IHC for pSTAT3 on adult back skin from HPV8-E6tg and HPV8-E6tg (K136N) mice. Scale bar = 40μm. (H) P300, α-tubulin and DAPI immunofluorescence labeling of cultured vector control, HPV8 E6 and E7 transduced PM1 keratinocytes, with quantification (n=3 per cell line). Scale bar = 40μm. Data are presented as mean ± SEM. NS, non-significant. (I and J) String™ analysis demonstrating the interaction of known HPV8 E2 (I) and E7 (J) protein binding partners and STAT3. Line colours define interactions as experimentally determined (pink) or from curated database (blue). (K) P300, pSTAT3 Y705 and ΔNp63 immunoblot, with GAPDH endogenous control, of vector control transduced HaCaT keratinocytes treated with scrambled control and p300 targeting siRNA, with quantification (n=3 per group). (L) STAT3 and ΔNp63 immunoblot, with GAPDH endogenous control, of vector control transduced HaCaT keratinocytes treated with scrambled control and STAT3 targeting siRNA, with quantification (n=3 per group). Statistical test(s) Supplemental Figure 4A, E, F, K and L 2-tailed Student's t-test, Supplemental Figure 4B, C, D and H one-way ANOVA. *$P$<0.05; **$P$<0.01; ***$P$<0.001; ****$P$<0.0001.

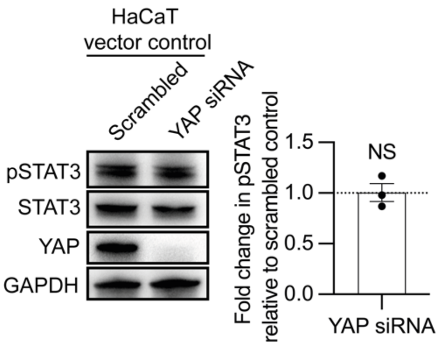


**Supplemental Figure 5. YAP siRNA treatment in established human keratinocytes, Related to Figure 6.** Total STAT3, pSTAT3 Y705 and YAP immunoblot, with GAPDH endogenous control, of vector control transduced HaCaT keratinocytes treated with scrambled control and YAP targeting siRNA, with quantification (n=3 per group). NS, non-significant. Statistical test 2-tailed Student's t-test.

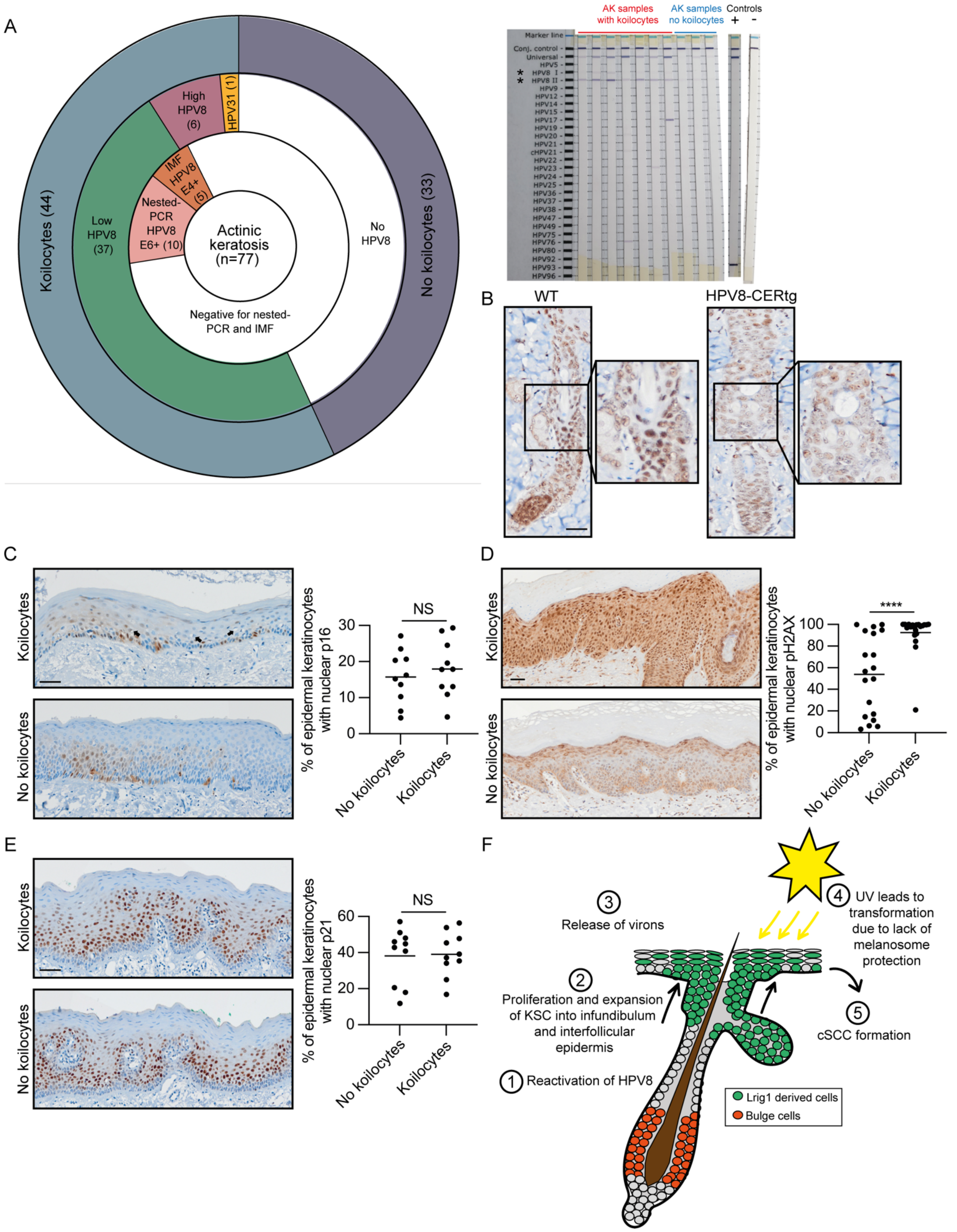


**Supplemental Figure 6. HPV8 associated with actinic keratosis with koilocytes, Related to Figure 7.** (A) Schematic of various approaches used to identify HPV8 in AK, with representative blot for HPV8 detection. (B) IHC of HPV8-CERtg and WT mouse back skin for Rb, with close-up of hair follicle junctional zone. Scale bar = 40μm. (C-E) IHC of human AK tissue for p16 (n=20), pH2AX (n=40) and p21 (n=20) with quantification. ****p < 0.0001 by Student's t-test. Scale bar = 50μm. Data are presented as mean ± SEM. NS, non-significant. Arrows indicate the presence of koilocytes. (F) Graphical illustration outlining HPV8 reactivation in human AK. Statistical test(s) Supplemental Figure 6C, D and E 2-tailed Student's t-test. ****$P$<0.0001.

**Supplemental Table 4. Patient characteristics based on the presence of koilocytes in actinic keratoses, Related to Figure 7**

| | All AK<br>(n=77) | AK with koilocytes<br>(n=44) | AK without koilocytes<br>(n=33) |
|---|---|---|---|
| **Age at time of surgery** | | | |
| Mean (SD) | 70 (9) | 70 (9) | 69 (9) |
| Minimum-Maximum | 48-89 | 49-88 | 48-89 |
| | | | |
| **Gender** | | | |
| Male | 28 (36%) | 20 (45%) | 8 (24%) |
| Female | 49 (64%) | 24 (55%) | 25 (76%) |
| | | | |
| **Body Site** | | | |
| Head and Neck | 46 (62%) | 27 (61%) | 19 (63%) |
| Torso | 7 (9%) | 5 (11%) | 2 (7%) |
| Arm | 13 (18%) | 6 (14%) | 7 (23%) |
| Leg | 8 (11%) | 6 (14%) | 2 (7%) |
| | | | |
| **Pathology findings** | | | |

| | | | |
|---|---|---|---|
| Bowenoid | 4 (5%) | 4 (9%) | 0 |
| Acantholytic | 2 (3%) | 2 (5%) | 0 |
| Hyperkeratotic/hypertrophic lesion | 8 (10%) | 5 (11%) | 3 (9%) |
| Lichenoid infiltrate | 3 (5%) | 1 (2%) | 3 (9%) |

**Supplemental Table 5. Resources table**

| REAGENT or RESOURCE | SOURCE | IDENTIFIER |
|---|---|---|
| **Antibodies** | | |
| anti-human Cytokeratin 14 (LL002) | Abcam | Cat# ab7800; RRID: AB_306091 |
| anti-human Involucrin | Abcam | Cat# ab68; RRID: AB_305656 |
| Mouse LRIG1 Alexa Fluor 488-conjugate antibody | R&D Systems | Cat# FAB3688G; RRID: AB_10888489 |
| Normal Goat IgG Alexa Fluor 488-conjugated control | R&D Systems | Cat# IC108G; RRID: AB_10890944 |
| Anti-LRIG1 Rabbit Polyclonal Antibody (Alexa Fluor 647) | VWR | Cat# 10330-520; RRID: AB_3068582 |
| Rabbit IgG Isotype Control, Alexa Fluor 647 Conjugated | Bioss | Cat# bs-0295P-A647; RRID: AB_3068583 |
| Anti-mouse CD34 PE | BD Biosciences | Cat# 551387; RRID: AB_394176 |

| | | |
|---|---|---|
| BD Pharmingen PE Rat IgG2a, k Isotyope Control | BD Biosciences | Cat# 553930; RRID: AB_479719 |
| Mouse LRIG1 Antibody | R&D Systems | Cat# AF3688; RRID: AB_2138836 |
| Anti-CD34 antibody [MEC 14.7] | Abcam | Cat# ab8158; RRID: AB_306316 |
| Phospho-Stat3 (Tyr705) (D3A7) XP® Rabbit mAb | Cell Signaling | Cat# 9145; RRID: AB_2491009 |
| Phospho-Stat3 (Ser727) Antibody | Cell Signaling | Cat# 9134; RRID: AB_331589 |
| Anti-GFP antibody | Abcam | Cat# ab13970; RRID: AB_300798 |
| Anti-Ki67 antibody | Abcam | Cat# ab15580; RRID: AB_443209 |
| YAP (D8H1X) XP® Rabbit mAb | Cell Signaling | Cat# 14074; RRID: AB_2650491 |
| Anti-p63 antibody [4A4] | Abcam | Cat# ab735; RRID: AB_305870 |
| Anti-ΔNp63 | Biolegend | Cat# 619002; RRID: AB_2207170 |

| | | |
|---|---|---|
| TBP Antibody | Cell Signaling | Cat# 8515; RRID: AB_10949159 |
| Stat3 (124H6) Mouse mAb | Cell Signaling | Cat# 9139; RRID: AB_331757 |
| Lamin A/C (4C11) Mouse mAb | Cell Signaling | Cat# 4777; RRID: AB_10545756 |
| p300 (E8S2V) Rabbit mAb | Cell Signaling | Cat# 57625; RRID: AB_3068009 |
| alpha Tubulin Monoclonal Antibody | ThermoFisher Scientific | Cat# A11126; RRID: AB_2534135 |
| Acetyl-Stat3 (Lys685) Antibody | Cell Signaling | Cat# 2523; RRID: AB_561524 |
| Stat3 (79D7) Rabbit mAb | Cell Signaling | Cat# 4904; RRID: AB_331269 |
| YAP (1A12) Mouse mAb | Cell Signaling | Cat# 12395; RRID: AB_2797897 |
| p53 (PAb 240) antibody | Thermo Fisher Scientific | Cat# MA5-15244; RRID: AB_10981260 |
| p21 (12D1) Rabbit mAb | Cell Signaling | Cat# 2947; RRID: AB_823586 |

| | | |
|---|---|---|
| Rb (EPR17512) antibody | Abcam | Cat# ab181616; RRID: AB_2848193 |
| Phospho-Histone H2A.X (Ser139) (20E3) Rabbit mAb | Cell Signaling | Cat# 9718; RRID: AB_2118009 |
| DAPI | Sigma-Aldrich | Cat# **10236276001** |
| eBioscience™ Fixable Viability Dye eFluor™ 780 | Thermo Fisher Scientific | Cat# 65–0865-14 |
| TO-PRO-3 Iodide (642/661) | ThermoFisher Scientific | Cat# T3605 |
| GAPDH | Merck Millipore | Cat# MAB374; RRID: AB_2107445 |
| Donkey anti-Goat IgG (H+L) Cross-Adsorbed Secondary Antibody, Alexa Fluor 488 | Invitrogen | Cat# A-11055; RRID: AB_2534102 |
| Goat anti-Rabbit IgG (H+L) Highly Cross-Adsorbed Secondary Antibody, Alexa Fluor 568 | Invitrogen | Cat# A-11011; RRID: AB_143157 |
| Goat anti-Mouse IgG (H+L) Cross-Adsorbed Secondary Antibody, Alexa Fluor 647 | Invitrogen | Cat# A-21235; RRID: AB_2535804 |
| Goat anti-Mouse IgG2a Cross-Adsorbed Secondary Antibody, Alexa Fluor 647 | Invitrogen | Cat# A-21241; RRID: AB_2535810 |
| Goat anti-Mouse IgG (H+L) Cross-Adsorbed Secondary Antibody, Alexa Fluor 568 | Invitrogen | Cat# A-11004; RRID: AB_2534072 |

| | | |
|---|---|---|
| Goat anti-Rabbit IgG (H+L) Cross-Adsorbed Secondary Antibody, Alexa Fluor 488 | Invitrogen | Cat# A-11008; RRID: AB_143165 |
| Goat anti-Mouse IgG1 Cross-Adsorbed Secondary Antibody, Alexa Fluor 488 | Invitrogen | Cat# A-21121; RRID: AB_2535764 |
| Goat anti-Rat IgG (H+L) Cross-Adsorbed Secondary Antibody, Alexa Fluor 568 | Invitrogen | Cat# A-11077; RRID: AB_2534121 |
| Goat anti-Rabbit IgG H&L (HRP) | Abcam | Cat# ab97051; RRID: AB_10679369 |
| Goat anti-Mouse IgG1 (HRP) | Abcam | Cat# ab98693; RRID: AB_10674928 |
| Goat anti-Mouse IgG2a heavy chain (HRP) | Abcam | Cat# ab97245; RRID: AB_10680049 |
| | | |
| **Chemicals, Peptides and Recombinant Proteins** | | |
| DMEM | ThermoFisher Scientific | Cat# 11965092 |
| DMEM:F12 | ThermoFisher Scientific | Cat# 21331-020 |
| EpiLife$^{TM}$ CF kit | ThermoFisher Scientific | Cat# MEPICF500 |
| Glutamax | ThermoFisher Scientific | Cat# 35050038 |
| Opti-MEM | ThermoFisher Scientific | Cat# 31985062 |

| | | |
|---|---|---|
| 0.05% Trypsin-EDTA | ThermoFisher Scientific | Cat# 25300054 |
| TrypLE Express Enzyme | ThermoFisher Scientific | Cat# 12604021 |
| Fetal bovine serum | ThermoFisher Scientific | Cat# 10270106 |
| Donor bovine serum | ThermoFisher Scientific | Cat# 16030074 |
| Penicillin-Streptomycin | ThermoFisher Scientific | Cat# 15140122 |
| Epidermal Growth Factor | PeproTech Ltd | Cat# AF-100-15 |
| Hydrocortisone | ThermoFisher Scientific | Cat# A16292.03 |
| Cholera toxin | Sigma-Aldrich | Cat# C8052 |
| Transferrin | Sigma-Aldrich | Cat# 11107018 |
| Liothyronine | Sigma-Aldrich | Cat# 1368008 |
| Insulin | Sigma-Aldrich | Cat# I0516 |
| Tamoxifen | Sigma-Aldrich | Cat# T5648 |
| Mifepristone (RU486) | Selleckchem | Cat# S2606 |
| Isoflurane | Boehringer Ingelheim Animal Health UK Ltd | Cat# 3661103035138 |
| Dispase | Worthington | Cat# LS02104 |
| Collagenase IV | Worthington | Cat# LS004188 |
| Hyaluronidase | Worthington | Cat# LS005475 |

| | | |
|---|---|---|
| DNAse I | Stem Cell Technologies | Cat# 07900 |
| Donkey Serum | Sigma-Aldrich | Cat# D9663 |
| VECTASHIELD® Antifade Mounting Medium | Vector Laboratories | Cat# H-1000-10 |
| Protease Inhibitor Cocktail (100x) | Cell Signalling | Cat# 5871S |
| QuantiTect Reverse Transcription Kit | Qiagen | Cat# 205313 |
| TaqMan® Universal Master Mix II | ThermoFisher Scientific | Cat# 4440040 |
| SYBR-Green Master Mix | ThermoFisher Scientific | Cat# 4309155 |
| Bovine Serum Albumin Powder | Fisher Bioreagents | Cat# BP9702-100 |
| Paraformaldehyde | TAAB | Cat# F017/2 |
| TWEEN®20 | Sigma-Aldrich | Cat# P9416 |
| Triton X-100 | Sigma-Aldrich | Cat# A16046.AE |
| Immobilon Forte Western HRP substrate | Merck Millipore | Cat# WBLUF0100 |
| RIPA buffer | ThermoFisher Scientific | Cat# 89900 |

| | | |
|---|---|---|
| Restore™ PLUS Western Blot Stripping Buffer | ThermoFisher Scientific | Cat# 46430 |
| Trans-Blot Turbo RTA Mini 0.2 µm PVDF Transfer Kit, for 40 blots | Bio-Rad | Cat# 1704272 |
| TGX™ FastCast™ Acrylamide Kit, 7.5% | Bio-Rad | Cat# 1610171 |
| 96 well plates, flat bottomed, white walled | Greiner Bio-One | Cat# 655098 |
| MicroAmp Optical 96-well reaction plate with barcode | ThermoFisher Scientific | Cat# 4306737 |
| Pierce™ BCA Protein Assay Kit | ThermoFisher Scientific | Cat# 23225 |
| Lipofectamine 3000 reagent | ThermoFisher Scientific | Cat# L3000008 |
| Crystal violet | ThermoFisher Scientific | Cat# V5265 |
| **Complementary DNA** | | |
| | | |
| **Retroviral Expression Vector** | | |
| Moloney murine leukemia retrovirus vector pLXSN | Available upon request | NA |
| pLXSN-8E6 vector | Available upon request | NA |
| pLXSN-8E7 vector | Available upon request | NA |
| **Critical Commercial Assays** | | |
| RNeasy Plus Mini Kit | Qiagen | Cat# 74134 |

| | | |
|---|---|---|
| RNeasy Plus Micro Kit | Qiagen | Cat# 74034 |
| QuantiTect Reverse Transcription Kit | Qiagen | Cat# 205314 |
| QIAamp DNA Mini Kit | Qiagen | Cat# 51304 |
| CellTiter-Glo® Luminescent Cell Viability Assay | Promega | Cat# G7572 |
| Warthin-Starry staining kit | Abcam | Cat# ab150688 |
| VECTASTAIN® Elite ABC-HRP Kit, Peroxidase | VECTOR Laboratories | Cat# PK-6100 |
| VECTASTAIN® Elite ABC-AP Kit, Alkaline Phosphotase | VECTOR Laboratories | Cat# AK-5000-1 |
| Pierce™ Co-Immunoprecipitation Kit | ThermoFisher Scientific | Cat# 26149 |
| NE-PER™ Nuclear and Cytoplasmic Extraction Reagents | ThermoFisher Scientific | Cat# 78835 |
| High-Sensitivity ChIP Kit | Abcam | Cat# ab185913 |
| RHA kit Stain (beta) HPV kit | Labo Bio-Medical Products BV | Cat# SPF10-LiPA25 |
| **Biological Samples** | | |
| Paraffin embedded human tumour samples | Hywel Dda and Cardiff and Vale University Health Boards | |
| **Experimental Models: Cell Lines** | | |

| | | |
|---|---|---|
| HaCaT | NA | NA |
| PM1 | Gifted by Sigrun Smola | |
| J2-3T3 | ATCC (CCL-92) | |
| HaCaT PLXSN (control) | NA | NA |
| HaCaT HPV8 E6 | NA | NA |
| HaCaT HPV8 E7 | NA | NA |
| PM1 PLXSN (control) | Gifted by Sigrun Smola | NA |
| PM1 HPV8 E6 | Gifted by Sigrun Smola | NA |
| PM1 HPV8 E7 | Gifted by Sigrun Smola | NA |
| **Experimental Models: Organisms/Strains** | | |
| Mouse: FVB/NCrl | Charles River, UK | |
| Mouse: Krt14-HPV8-CER/FVB | Schaper et al., 2005 (39) | NA |
| Mouse: Krt14-HPV8-E6/FVB | Marcuzzi et al., 2009 (43) | NA |
| Mouse: Krt14-HPV8-E2/FVB | Pfefferle et al., 2008 (42) | NA |
| Mouse: Krt14-HPV8-E7/FVB | Heuser et al., 2016 (41) | NA |

| | | |
|---|---|---|
| Mouse: $Stat3^{WT/LoxP}$/FVB | De Andrea et al., 2010 (49) | NA |
| Mouse: $Stat3^{WT/WT}$/FVB | De Andrea et al., 2010 (49) | NA |
| Mouse: B6.129P2-*Gt(ROSA)26Sor*$^{tm1(CAG-Brainbow2.1)Cle}$/J | The Jackson Labs | Cat# 017492; RRID: IMSR_JAX:017492 |
| Mouse: *Lrig1*$^{tm1.1(cre/ERT2)Rjc}$/J (Lrig1-CreERT2) | The Jackson Labs | Cat# 018418; RRID: MSR_JAX:018418 |
| Mouse: B6;SJL-Tg(Krt1-15-cre/PGR*)22Cot/J | The Jackson Labs | Cat# 005249; RRID: IMSR_JAX:005249 |
| Mouse: Lrig1-EGFP-ires-CreERT2 | Gifted by Kim Jensen (46) | NA |
| **Oligonucleotides (Taqman Probe)** | | |
| ACTB (FAM-MGB) | ThermoFisher Scientific | Cat# Hs00357333_g1 |
| ACTB (FAM-MGB) | ThermoFisher Scientific | Cat# Mm02619580_g1 |
| GAPDH (FAM-MGB) | ThermoFisher Scientific | Cat# Hs02786624_g1 |
| GAPDH (FAM-MGB) | ThermoFisher Scientific | Cat# Mm99999915_g1 |
| cMYC (FAM-MGB) | ThermoFisher Scientific | Cat# Mm01192721_m1 |
| BCAM (FAM-MGB) | ThermoFisher Scientific | Cat# Mm00522336_g1 |
| MAML1 (FAM-MGB) | ThermoFisher Scientific | Cat# Mm01295191_g1 |

| | | |
|---|---|---|
| NDST2 (FAM-MGB) | ThermoFisher Scientific | Cat# Mm00447818_m1 |
| PTMS (FAM-MGB) | ThermoFisher Scientific | Cat# Mm07298679_m1 |
| TAOK2 (FAM-MGB) | ThermoFisher Scientific | Cat# Mm01139108_m1 |
| TSC22D4 (FAM-MGB) | ThermoFisher Scientific | Cat# Mm00470231_m1 |
| ΔNP63 (FAM-MGB) | ThermoFisher Scientific | Cat# Mm01169470_m1 |
| CD34 (FAM-MGB) | ThermoFisher Scientific | Cat# Mm00519283_m1 |
| LGR5 (FAM-MGB) | ThermoFisher Scientific | Cat# Mm00438890_m1 |
| LGR6 (FAM-MGB) | ThermoFisher Scientific | Cat# Mm05916284_s1 |
| KRT10 (FAM-MGB) | ThermoFisher Scientific | Cat# Mm03009921_m1 |
| SOX9 (FAM-MGB) | ThermoFisher Scientific | Cat# Mm00448840_m1 |
| BCAM (FAM-MGB) | ThermoFisher Scientific | Cat# Hs00170663_m1 |
| MAML1 (FAM-MGB) | ThermoFisher Scientific | Cat# Hs01070499_m1 |
| NDST2 (FAM-MGB) | ThermoFisher Scientific | Cat# Hs00234335_m1 |

| | | |
|---|---|---|
| PTMS (FAM-MGB) | ThermoFisher Scientific | Cat# Hs00767048_s1 |
| TAOK2 (FAM-MGB) | ThermoFisher Scientific | Cat# Hs00191170_m1 |
| TSC22D4 (FAM-MGB) | ThermoFisher Scientific | Cat# Hs00229526_m1 |
| ΔNP63 (FAM-MGB) | ThermoFisher Scientific | Cat# Hs00978339_m1 |
| **Genotyping primers** | | |
| CER-HPV8: 842 bp, E6 HPV8 (F) CAATTTTCCTAAGCAAATGGAC 22 E7 HPV8 (R) CACTACATTCAGCTTCCAAAATACA | Sigma Aldrich | NA |
| E6-HPV8: 504 bp, F: GGATCCTTTCCTAAGCAAATGGACGG G R: GGATCCGCATGCCACAAAATCTTGCA CAGTGACCTC | Sigma Aldrich | NA |
| Brainbow confetti Gt(ROSA)26Sor: 300 bp (mutant) and 386 bp (wt), Mutant Forward GAA TTA ATT CCG GTA TAA CTT CG, Wild type Forward AAA GTC GCT CTG AGT TGT TAT, Common CCA GAT GAC TAC CTA TCC TC | Sigma Aldrich | NA |
| CRE, CRE A TGACCGTACACCAAAATTTG, CRE B ATTGCCCCTGTTTCACTATC | Sigma Aldrich | NA |

| | | |
|---|---|---|
| **Oligonucleotides (ChIP primers)** | | |
| ΔNP63 sense (5'-CATAGATGCATCACGTGCA-3') | Sigma Aldrich | NA |
| ΔNP63 antisense (5'-GCAATTACAAAATAAGCTACCTG-3') | Sigma Aldrich | NA |
| **siRNA (ON-TARGETplus SMARTpool)** | | |
| Non-Targeting | Dharmacon | Cat# D-001810-10-20 |
| P300 | Dharmacon | Cat# L-003486-00-0005 |
| YAP | Dharmacon | Cat# L-012200-00-0005 |
| STAT3 | Dharmacon | Cat# L-003544-00-0005 |
| **Other** | | |
| Migration assay inserts | Ibidi | Cat# 80209 |
| microTUBE-50 AFA Fiber Screw-Cap tubes | Covaris | Cat# 520166 |
| **Deposited data** | | |
| Sequence data (Figure 1 and 2; Supplemental Figure 1) | This paper | GSE248056 |
| Sequence data (Figure 4; Supplemental Figure 3) | This paper | GSE248056 |
| **Software and Algorithms** | | |
| FlowJo v10.9.0 | Becton Dickinson & Company | https://www.flowjo.com |
| GraphPad Prism v9 | GraphPad Software | https://www.graphpad.com |
| Image J v1.53t | NIH | https://imagej.nih.gov/ij/ |

| | | |
|---|---|---|
| Qupath v0.4.4 | Bankhead *et al.*, 2017 (119) | https://qupath.github.io |
| ImageLab v6.1 | Bio-Rad software | https://www.bio-rad.com |
| Imaris 10.0 | Oxford Instruments software | https://imaris.oxinst.com |
| GSEA MsigDB v2023.1.Mm | Subramanian *et al.*, 2005 (47); Mootha *et al.*, 2003 (48) | https://www.gsea-msigdb.org/gsea/index.jsp |
| Ingenuity Pathway Analysis 23.0 | Qiagen | https://digitalinsights.qiagen.com/products-overview/discovery-insights-portfolio/analysis-and-visualization/qiagen-ipa/ |
| RStudio v1.4.1564 | | https://cran.r-project.org |
| **Other** | | |
| BD LSRFortessa | BD Biosciences | |
| Axio Scan.Z1 Slide Scanner | Zeiss | |
| QuantStudio$^{TM}$ 7 Flex Real-Time PCR System | ThermoFisher Scientific | |

| | | |
|---|---|---|
| GelCount™ Colony Counter | Oxford Optronix | |
| CLARIOstar plate reader | BMG LABTECH | |
| Covaris® M220 ultrasonicator | Covaris | |

## Supplemental Methods

### Cell lines

HaCaT and PM1 cells represent spontaneously immortalized human keratinocyte cell lines. J2-3T3 cells are derived from mouse embryonic fibroblast cells and are a subclone of the original mouse embryonic fibroblast line, 3T3-Swiss Albino. HaCaT cell lines were maintained in T75 flasks with DMEM-high glucose, 10% fetal bovine serum, 1% L-glutamine, 1% penicillin-streptomycin media at 37°C in a 5% $CO_2$ incubator. PM1 cells were maintained in T75 flasks in RM+ media (consisting of DMEM and DMEM:F12 at a ratio of 3:1) supplemented with fetal bovine serum, 1% L-glutamine, 0.4μg hydrocortisone, $10^{-10}$ M cholera toxin, 5μg/mL transferrin, $2x10^{-11}$ M liothyronine, 5μg/mL insulin, 10ηg/mL epidermal growth factor, 1% penicillin-streptomycin. J2-3T3 cells were maintained in T75 flasks in DMEM-high glucose, 10% donor bovine serum, 1% L-glutamine media at 37°C in a 5% $CO_2$ incubator and were used as feeder cells when growing primary mouse keratinocytes.

### Immunofluorescence staining and microscopy of OCT sections

For paraffin embedded samples, slides were heated at 60°C for 30 minutes, then deparaffinized with xylene (2 x 10 minutes) and decreasing concentrations of ethanol (100-75%) for 5 minutes each, followed by a 5 min wash in PBS. For antigen retrieval, samples

were submerged in citrate buffer (pH6.0) and heated in a microwave for 10 minutes in a pressure cooker and allowed to cool to room temperature gradually. Samples were washed for 5 minutes in PBS, then a hydrophobic barrier pen was used to draw around the tissue sample. Sections were then incubated in blocking buffer (10% goat serum in PBS) for 1 hour at room temperature. Primary antibodies (Supplemental Table 5) were diluted in 5% goat serum and incubated overnight at 4°C. Samples were washed 4 x 5 minutes in PBS Tween-20 (0.05%) before incubating with fluorescence-conjugated secondary antibodies in PBS for 1 hour in the dark at room temperature. Samples were again washed 4 x 5 minutes in PBS Tween-20 (0.05%) before mounting with Vectashield mounting solution (Vector Labs). For frozen OCT samples, sections were fixed in 4% paraformaldehyde for 10 minutes at room temperature, then washed 3 x 5 minutes in PBS before being permeabilized with 0.25% PBS/Tween-20 for 10 minutes at room temperature. Blocking, primary and secondary antibody steps were carried out as described above. Finally, sections were mounted with Vectashield and scanned on a Olympus Slideview VS2000 slide scanner.

**Immunohistochemistry staining**

Tissue sections were incubated overnight with anti-pSTAT3 (1:300, Cell Signalling, #9145), anti-p63 (1:300, Abcam, #ab735), anti-YAP (1:400, Cell Signalling, #14074), anti-pp53 (1:1000, ThermoFisher, #MA5-15244), anti-p21 (1:400, Cell Signalling, #2947), anti-Rb1 (1:1000, Abcam, #ab181616), anti-pH2AX (1:1000, Abcam, #9718) or anti-pan cytokeratin (1:2000, Novus Biologicals #NBP2-29429) diluted in 5% goat serum overnight at 4°C. Sections were washed in PBS-Tween (0.05%) then incubated for 1 hour at room temperature with biotinylated species-specific secondary antibodies diluted in 5% serum. Secondary antibodies used were: Goat anti-Mouse IgG Antibody (Vector Laboratories, BP-9200) and Goat anti-Rabbit IgG Antibody (Vector Laboratories, BP-9100). Expression was detected using ABC-HRP for 30 minutes at room temperature and then DAB stained until

optimal staining was observed. Sections were then counterstained with haematoxylin (Atom Scientific) and dehydrated using a series of ethanol and xylene rinses and mounted with DPX mountant (Merck). Images were acquired using an Olympus Slideview VS2000 slide scanner.

**Fluorescence-activated cell sorting (FACS) or analysis**

Samples were gated on the basis of forward- and side-scatter. Doublets and dead cells were excluded. eBioscience™ Fixable Viability Dye eFluor™ 780 (Thermo Fisher Scientific #65–0865-14) was used to gate out dead cells when sorting Confetti (GFP, YFP, RFP and CFP) mice. Single stained samples were used as compensation controls and use of an isotype control was used to determine background fluorescence. Data was processed using FlowJo analysis software (FlowJo, LLC).

**Protein extraction and quantification**

Cells in culture were collected and counted. Adherent cells in culture were detached using Versene or TrypLE (ThermoFisher Scientific). Cells were centrifuged at 250xg for 5 minutes and pellet was washed with PBS, and again pelleted. PBS was removed, and pellet resuspended in 100 μL per $1x10^6$ cells of RIPA buffer containing protease inhibitor cocktail (Cell Signalling). Samples were homogenized by pipetting and incubated for 30 minutes on ice. Following incubation, samples were centrifuged at 10,000xg for 10 minutes at 4°C and the supernatant was collected. Nuclear protein lysates were extracted as outlined in manufacturers guidelines (ThermoFisher Scientific). The BCA assay kit (Pierce, ThermoFisher Scientific) was used to determine protein concentrations.

**Western immunoblotting**

Laemmli buffer (4x) was added to the samples, which were then heated at 95°C for 5 minutes. TGX™ FastCast™ premixed acrylamide solutions (BioRad) were used to cast gels. 10 μg of protein were loaded into the wells. Once the samples were loaded, gels were run at 250V until the desired marker separation was achieved. Trans-Blot® Turbo™ Transfer System (BioRad) was used to transfer protein to the PVDF membrane. Following confirmation of transfer and washing steps in TBS/T, membranes were incubated with gentle agitation in blocking buffer of 10% milk or BSA (depending on antibody) in TBS/T for 1 hour at room temperature. Following incubation, membranes were incubated in the desired primary antibody diluted in 5% BSA or milk in TBS/T and incubated overnight at 4°C on a roller. The following antibodies were used for immunoblotting: anti-phospho-STAT3 Y705 (1:500, Cell Signalling, 9145), anti-phospho-STAT3 S727 (1:500, Cell Signalling, 9134), anti-total STAT3 (1:1,000, Cell Signalling, 9139), anti-ΔNp63 (1:500, Biolegend, 619002), anti-involucrin (1:500, Abcam, ab68), anti-p300 (1:1,000, Cell Signalling, 57625), anti-acetyl-STAT3 Lys685 (1:500, Cell Signalling, 2523), anti-GAPDH (1:10,000, Millipore, MAB374), anti-Lamin A/C (1:2000, Cell Signalling, 4777), anti-TBP (1:1000, Cell Signalling, 8515). Anti-GAPDH was used as a loading control for whole protein lysates and both anti-Lamin A/C and anti-TBP were used as loading controls for nuclear protein lysates. Following incubation, the membrane is washed 4 x 5 minutes in TBS/T before incubating in HRP-conjugated secondary antibody (Abcam) diluted 1:2000 in TBS/T for 1 hour at room temperature. The following secondary antibodies were used for immunoblotting: goat anti-rabbit IgG H&L HRP (Abcam), goat anti-mouse IgG1 (Abcam), goat anti-mouse IgG2a (Abcam). Membranes are washed again for 4 x 5 minutes in TBS/T before antibody binding was detected by incubating Illumina Forte chemiluminescence reagent (Millipore) on the membrane for 30-60 seconds and membrane imaged using the ChemiDoc MP Imaging System (BioRad). Western blot data were quantified by

densitometry, using ImageLab software (BioRad). The pixel density over the selected areas was quantified and compared.

**Co-Immunoprecipitation**

Briefly, columns were prepared by incubating 50μL of coupling resin to 50 μg of YAP (Cell Signalling, 14074) and STAT3 (Cell Signalling, 4904) antibodies. $1x10^6$ cells were lysed in 100μL of lysis buffer to yield ~300μg of protein. Control agarose resin slurry was used to pre-clear the protein lysate, before being added to the antibody-coupled resin and incubated with gentle mixing overnight at 4°C. Resins were then washed and co-immunoprecipitated proteins YAP and STAT3 were identified by Western blot with the antibodies anti-YAP (1:500, Cell Signalling, 12395) and anti-STAT3 (1:1000, Cell Signalling, 9139) respectively. To ensure Co-IP specificity, all pull downs were performed with the following controls: 1) a non-activated control resin to ensure no non-specific binding of the antibodies and lysate to the resin, 2) a quenched antibody coupling resin which is a resin that has been processed in the exact same way as the test resin but with the antibody present and 3) a non-relevant antibody which was unrelated to the study for all pulldowns shown.

**Chromatin Immunoprecipitation (ChIP)-qPCR**

The input amount of cells for each reaction was 25mg. Briefly, cells were cross-linked using 1% formaldehyde in cell culture media for 10 minutes at room temperature, and then quenched with 1.25 M Glycine on ice. Cells were collected by centrifugation at 250xg for 5 minutes, and pellet washed with ice cold PBS. Cells were pelleted again, resuspended in working lysis buffer and incubated on ice for 10 minutes, before being vortexed and centrifuged at 3000 rpm for 5 minutes. Supernatant was carefully removed and pellet

resuspended in 50 μL of ChIP buffer and incubated on ice for 10 minutes. For ChIP-qPCR roughly 100 μg of sheared chromatin and 5 μg of antibody were used. Chromatin was sheared to a fragment size of 200-600bp in a microTUBE-50 AFA Fiber Screw-Cap tube (Covaris) in a waterbath sonicator (Covaris). Sheared chromatin was centrifuged at 12000 rpm at 4°C for 10 minutes and supernatant transferred to a new tube. Input material was reserved. The remainder was included in a ChIP reaction (0.2 mL) PCR tube containing pre-incubated (bound) ChIP grade anti-STAT3 antibody (Cell Signalling, 4904), along with anti-RNA Polymerase II and non-Immune IgG which were used as positive and negative ChIP controls, respectively. Reactions were incubated at room temperature for two hours on an orbital shaker. Wells were then washed with wash buffer and DNA extracted using DNA Release Buffer. Cross-links were reversed by incubating with RNase A solution at 42°C for 30 minutes, then adding Proteinase K and incubating for a further 45 minutes at 60°C. DNA solution was transferred to a fresh PCR tube and incubated at 95°C for 15 minutes. DNA was purified by spin columns provided with the kit and eluted in DNA elution buffer. After purification, qPCR was used to analyze immunoprecipitated DNA with the following primers: ΔNp63–719, 5′-CATAGATGCATCACGTGCA-3′ (sense); ΔNp63–464, 5′-GCAATTACAAAATAAGCTACCTG-3′ (antisense) using SYBR Green qPCR Master mix (ThermoFisher Scientific).(1) Since RNA polymerase II is enriched in the GAPDH gene promoter, DNA immunoprecipitated by the RNA polymerase II antibody was used with GAPDH primers as a positive control. The amount of immunoprecipitated DNA was determined as the fraction of the input (amplification efficiency $^{(\mathrm{Ct\ Input} - \mathrm{Ct\ ChIP})}$) and normalized to IgG control. HPV8-E6 data were then plotted as fold change relative vector control.

## Cell proliferation assay

HaCaT/PM1-PLXSN, -E6 and -E7 cells were plated (5,000 cells per well) in a white-walled 96 well plate and left overnight to adhere. Cell proliferation was determined using the BrdU

cell proliferation ELISA kit (Abcam, ab126556) as per manufacturers guidelines. BrdU was allowed to incorporate with cells for 24 hours before ELISA assay was conducted. Luminescence was recorded using a CLARIOstar plate reader with wells containing only media serving as a blank. Blank corrected values were used to calculate the amount of proliferation when compared to vector control cells.

**Migration assay (established cell lines)**

HaCaT/PM1-PLXSN, -E6 and -E7 cells were seeded into ibidi culture inserts (2 well, Ibidi, #80209) at a density of 100,000 cells in 70 μL media (for each half of the insert) in a 24 well plate and allowed to adhere for 8 hours in the incubator. Cells were then checked for confluence and culture inserts were removed gently using a forceps. Cells were washed to remove any debris using PBS, and fresh media was placed in each well, before being placed in a Leica DM6000 timelapse microscope overnight for image acquisition every hour for 16 hours. Data were quantified by plotting the cell covered area against the time to show the rate of gap closure. The linear phase of gap closure was used to calculate the percentage of gap closure over a defined period of time relative to vector control cells.

**DNA extraction, precipitation and β-HPV genotyping PCR**

Briefly, an equal volume of 5M ammonium acetate was added to each sample and thoroughly mixed. Two volumes of 100% ethanol was added to each sample, thoroughly mixed and placed at -80°C for 30 minutes. Samples were removed from -80°C and centrifuged for 30 minutes at 14,000 x g at 4°C. After carefully removing the supernatant, 1 mL of 70% ethanol was added, thoroughly mixed, and centrifuged at 14,000 x g for 10 minutes at 4°C. The supernatant was again carefully discarded, and samples were placed in a hot block for 10-15 minutes at 37°C until the residual ethanol evaporated. Each sample

was then resuspended in 50 μL nuclease free water and DNA concentration determined using a NanoDrop 2000 (Thermo Fisher Scientific).

PCR was performed on each sample to amplify the relatively conserved β-HPV E1 region and PCR-amplified samples were run through a genotype hybridization protocol as per manufacturer's instructions. The positive PCR control was used from the kit, and Milli-Q water without DNA was used as a negative control. The master mix was first added to each PCR tube and 10 μL of sample was added and mixed by pipetting. PCR tubes were briefly spun and placed in a thermocycler as per the PCR protocol in the manual. Amplified PCR products were then hybridized for detection. 10 μL of the PCR-amplified sample, 10 μL Denaturation solution and 10 μL of 3B solution were added to their respective trough and mixed by pipetting. Following a 5-minute incubation at room temperature, 2 mL pre-warmed Hybridization solution was added to each trough and mixed by gently shaking the troughs. Test strips were then immediately placed into their respective troughs and troughs were placed into a pre-heated 50°C shaking water bath for 60 minutes at approximately 80 rpm. Following incubation, test troughs were removed from the shaking water bath and the troughs were carefully aspirated using a vacuum aspirator. Test strips were washed twice in 2 mL pre-warmed Stringent Wash Solution for 10 seconds by gently shaking the troughs. A final washing step was then performed via a 30-minute shaking water bath incubation with 2 mL Stringent Wash Solution. All wash steps and incubations were next performed on a rocker at room temperature using the highest possible speed and avoiding spillages. Samples were washed twice in diluted Rinse Solution for 1 minute. A 30-minute incubation was performed using 2 mL of Conjugate Solution. A further two wash steps were performed with 2mL Diluted Rinse Solution, and a third wash step using 2 mL Substrate Diluent.

Colour development was performed through incubation of each test strip with 2 mL Substrate Solution for 30 minutes and covering the troughs with foil. To stop colour development, strips were washed twice for 3 minutes in Milli-Q water. Strips were then

removed from the troughs and placed on absorbent paper until dry. Each strip was then cello taped to the results page and results were recorded.

**Nested PCR for HPV8 E6**

The outer set of primers was 5'-GCTGCTGTCAGGTCAAGCTA-3' (forward) and 5'-TCTTCCGGCCGAGAGTCTAT-3' (reverse) and the nested set of primers was 5'-TGCTGTCAGGTCAAGCTACG-3' (forward) and 5'-GCCTTCGCTTGGATTTCTGC-3'(reverse). The outer set flanked 545bp fragment 828 to 1372 and the nested set flanked 440bp fragment 830 to 1269 (GenBank accession no. M12737.1). DNA amplification was performed using GoTaq® G2 Hot Start Taq (Promega, Madison, WI). DNA extracted (2 μL) from each FFPE sample was used for the first PCR round with the outer primers, then 10 μL from each reaction was aliquoted and diluted 10-fold with Milli-Q water and 2 μL was then used in the reaction with nested primers. The total reaction volume in both rounds was 25 μL including 2 μL of templates, 200 ηM of primers, 50 μM of each of the four dNTPs and 0.4 U of Taq polymerase (Promega, Madison, WI).

Both rounds of PCR were performed as follows: 94°C, 5 minutes as the hot start step and then 30 seconds at 94°C, 1 minute 30 seconds at 60°C, and 1 minute 30 seconds at 72°C, 35 cycles of amplification. Products from both the first and the second round of PCR were electrophoresed on a 2% agarose gel and visualized under UV light after staining with SafeView (NBS Biologicals, Cambridgeshire, UK). A sample was considered positive if 545bp and 440bp fragments were detected in the first and second round respectively. Negative and positive controls were run in each reaction. The DNA quality was monitored by running a β-globin PCR using DNA extracted from each FFPE sample. β-globin primers were as follows: 5'-GAAGAGCCAAGGACAGGTAC-3' (GH20), 5'-CAACTTCATCCACGTTCACC-3' (PC04), 5'-GCTCACTCAGTGTGGCAAAG-3' (RS42), 5'-GGTTGGCCAATCTACTCCCAGG-3' (KM29), 5'-ATTTTCCCACCCTTAGGCTG-3' (RS40),

5'-TGGTAGCTGGATTGTAGCTG-3' (RS80). The PCR conditions for β-globin PCR were identical to the HPV8 PCR.

**Bioinformatic analysis**

For the Lrig1 vs CD34 experiment, cDNA libraries were generated and NEBNext sequencing adaptors were added along with sample barcodes. Libraries were then sequenced on a Illumina HiSeq4000 at a depth of 20M reads per sample. Sequenced reads were then run through FastQC and subsequently mapped to the murine GRCM38 reference genome using STAR. FeatureCounts were then used to quantify reads and DEG analysis was performed via the standard DESeq2 pipeline. For the confetti-positive cells (with and without Lrig1 cell surface expression) experiment, libraries were generated using the Illumina TruSeq RNA Library Prep Kit (v2) and were subsequently sequenced on a NovaSeq 6000 PE150 to generate a total of 20-million paired-end reads per sample. Raw sequencing data was processed by NovoGenes in-house pipelines to remove bad quality reads, map to the mouse GRCm39 reference genome using Hisat2 (v2.0.5) and generate normalized reads using featureCounts (v1.5.0). Normalized reads were used to generate PCA plots in order assess sample clustering. DEGs were then generated using edgeR (v3.22.5) using a false discovery rate (FDR) of 0.1 and then filtered for significance (adjusted p value $< 0.05$). DEGs were then returned to us from Novogene, where we performed pathway analysis with Gene Set Enrichment Analysis (GSEA) GSEAPreRanked tool and Ingenuity Pathway Analysis (Qiagen) software where causal analysis was run with default parameters.

The following gene sets used were taken from the Molecular Signatures Database (GSEA, Broad Institute): 1) "DANG_MYC_TARGETS_UP" used in Figure 1H, I and Supplemental Figure 3B; 2) "STAT3_02" used in Figure 2B, 4F, G, H; 3)

“GOBP_EPITHELIAL_TO_MESENCHYMAL_TRANSITION” used in Figure 4F, G and H; 4) “GOBP_KERATINOCYTE_DIFFERENTIATION” used in Figure 4G, Supplemental Figure 3B; 5) “GOBP_STEM_CELL_PROLIFERATION” used in Supplemental Figure 3B and C; 6) “GOBP_STEM_CELL_DIFFERENTIATION”, “GOBP_KERATINOCYTE_MIGRATION” and “GOBP_REGULATION_OF_KERATINOCYTE_MIGRATION” gene signatures were used in Supplemental Figure 3C. “Upregulated gene panel” and “Down regulated gene panel” gene signatures used in Supplemental Figure 1C were taken from Jensen *et al*., 2009 (2). “uHFII signature”, “uHFIII signature”, “Sebaceous gland”, “CD34 signature”, “OBI signature” and “OBII signature” gene signatures used in Supplemental Figure 1D were taken from Joost *et al*., 2016 (3). “Upper HF suprabasal”, “Differentiation TFs” and “Terminal Differentiation TFs” gene signatures used in Supplemental Figure 3C were taken from Joost *et al*., 2016 (3).

**Statistical analysis**

Figure 1B Student’s t-test, Figure 1J one-way ANOVA; Figure 2D, E, F and H Student’s t-test, Figure 2I one-way ANOVA; Figure 3A, B, C, D, E and F one-way ANOVA, Figure 3G and H Student’s t-test; Figure 4A, D and I one-way ANOVA, Figure 4C and J Student’s t-test; Figure 5A, C, D, E, F, H and I one-way ANOVA, Figure 5B, J, K, L and M Student’s t-test; Figure 6A, B, C, D and E Student’s t-test, Figure 6F one-way ANOVA; Figure 7B, C, D, E and F Student’s t-test; Supplemental Figure 1F one-way ANOVA; Supplemental Figure 2A and C one-way ANOVA, Supplemental Figure 2D Student’s t-test; Supplemental Figure 4A, E, F, K and L Student’s t-test, Supplemental Figure 4B, C, D and H one-way ANOVA; Supplemental Figures 6C, D and E Student’s t-test.

## References


1. Chu WK, et al. Transcriptional activity of the ΔNp63 promoter is regulated by STAT3. *Journal of Biological Chemistry*. 2008;283(12). doi:10.1074/jbc.M800183200

2. Jensen KB, et al. Lrig1 Expression Defines a Distinct Multipotent Stem Cell Population in Mammalian Epidermis. *Cell Stem Cell*. 2009;4(5). doi:10.1016/j.stem.2009.04.014

3. Joost S, et al. Single-Cell Transcriptomics Reveals that Differentiation and Spatial Signatures Shape Epidermal and Hair Follicle Heterogeneity. *Cell Syst*. 2016;3(3). doi:10.1016/j.cels.2016.08.010
