# Supplementary material for: HPV8-induced STAT3 activation led keratinocyte stem cell expansion in human actinic keratoses
Source: JCI Insight. 2024 Jun 25;9(15):e177898. doi: 10.1172/jci.insight.177898 (PMC11383611; doi:10.1172/jci.insight.177898)

Figure 2D – STAT3α/β

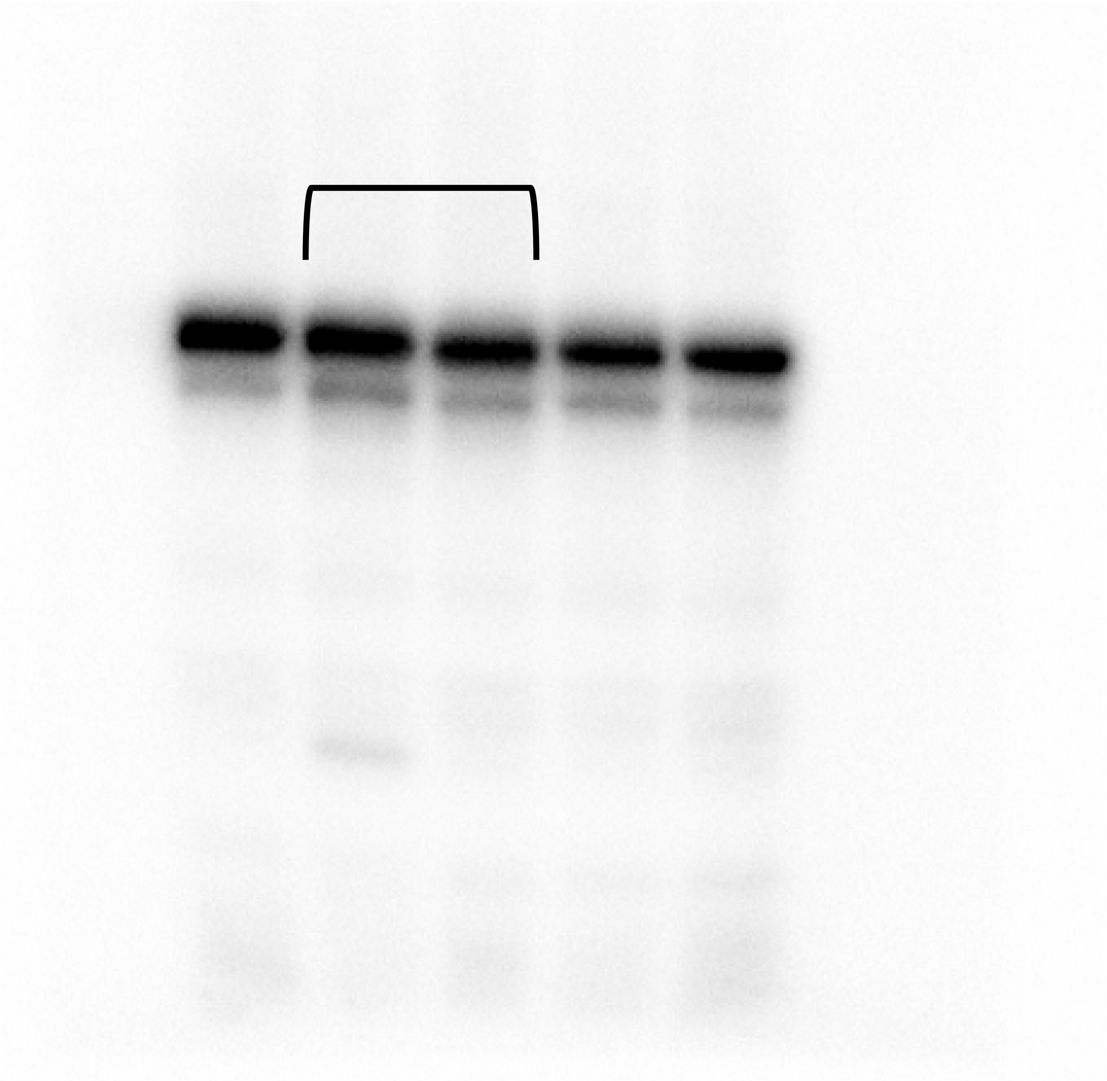

Figure 2D – GAPDH

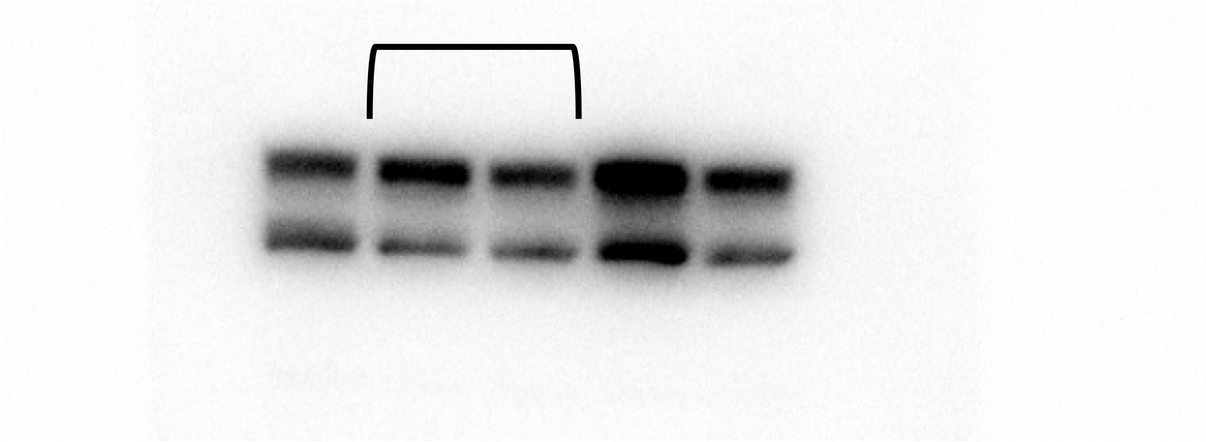

Figure 2E – pSTAT3 Y705

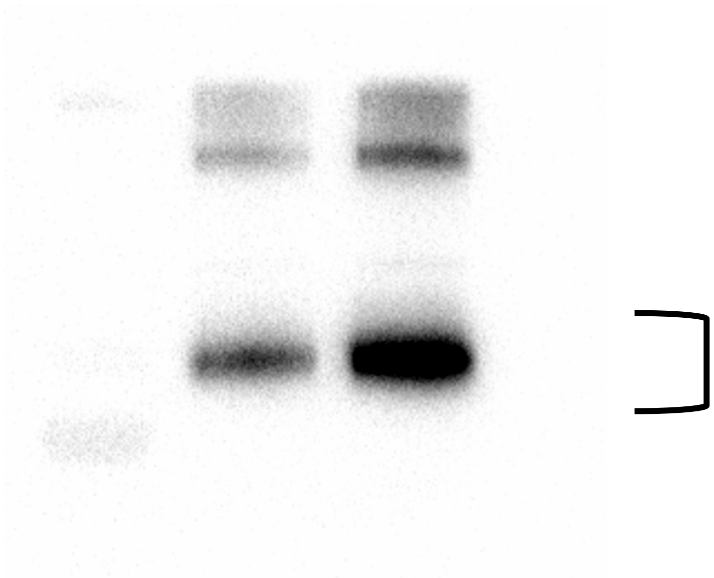

Figure 2E – pSTAT3 S727

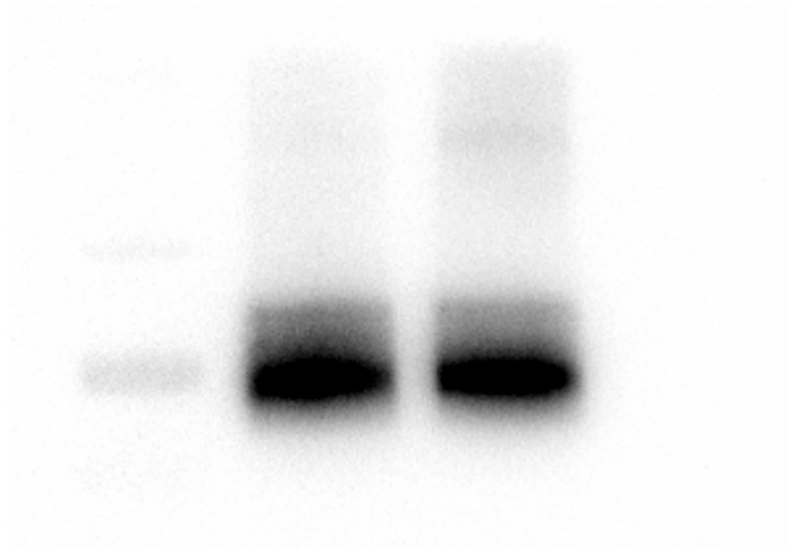

Figure 2E – TBP

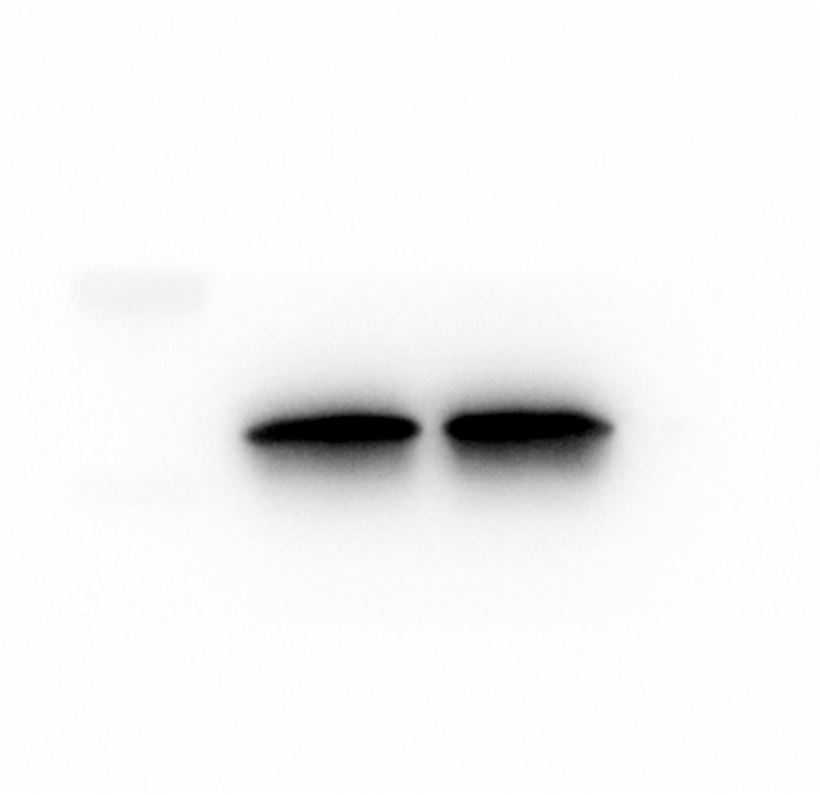

Figure 4A – pSTAT3 Y705

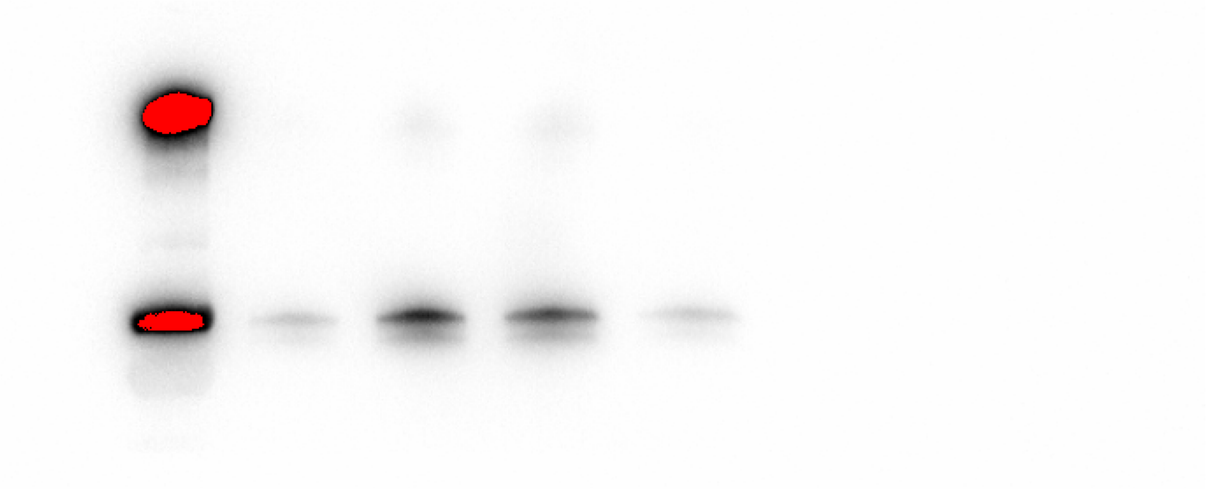

Figure 4A – TBP (pSTAT3 Y705 blot)

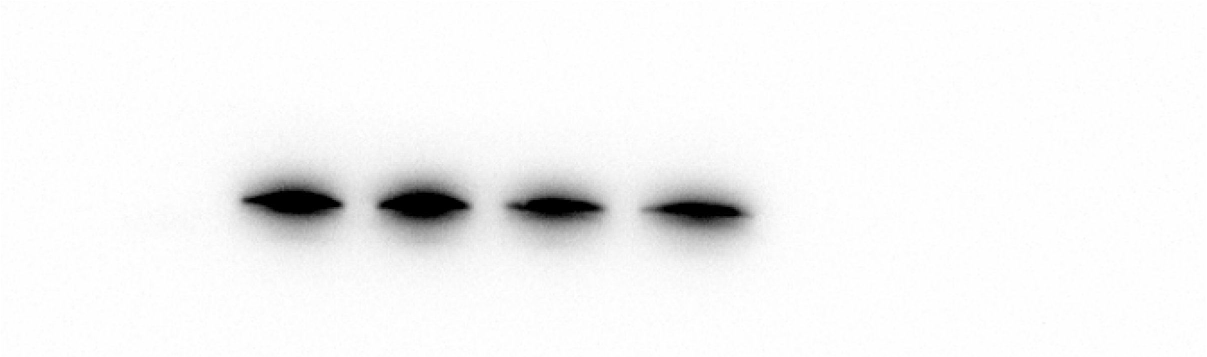

Figure 4A – pSTAT3 S727

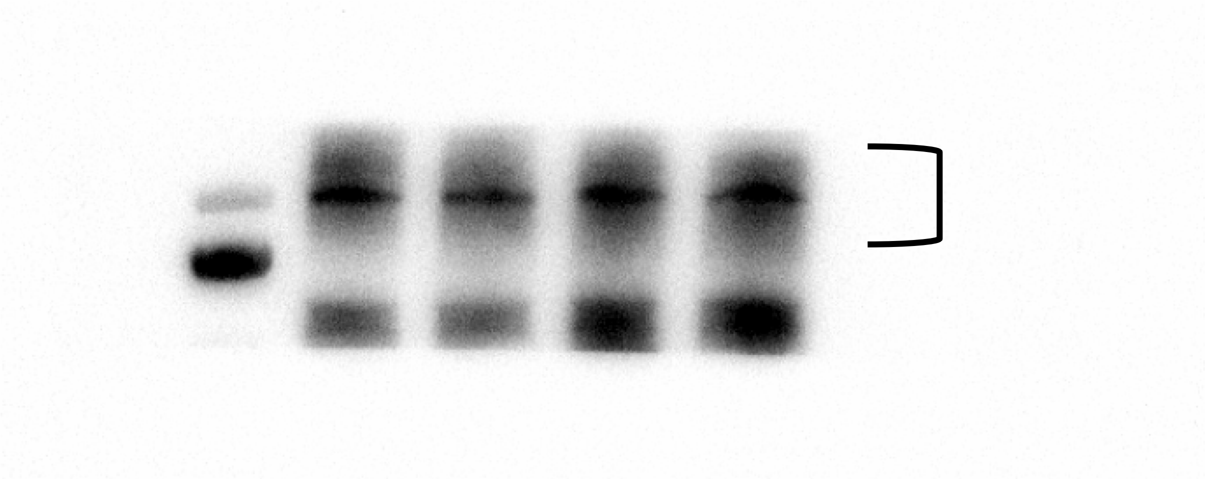

Figure 4A – TBP (pSTAT3 S727 blot)

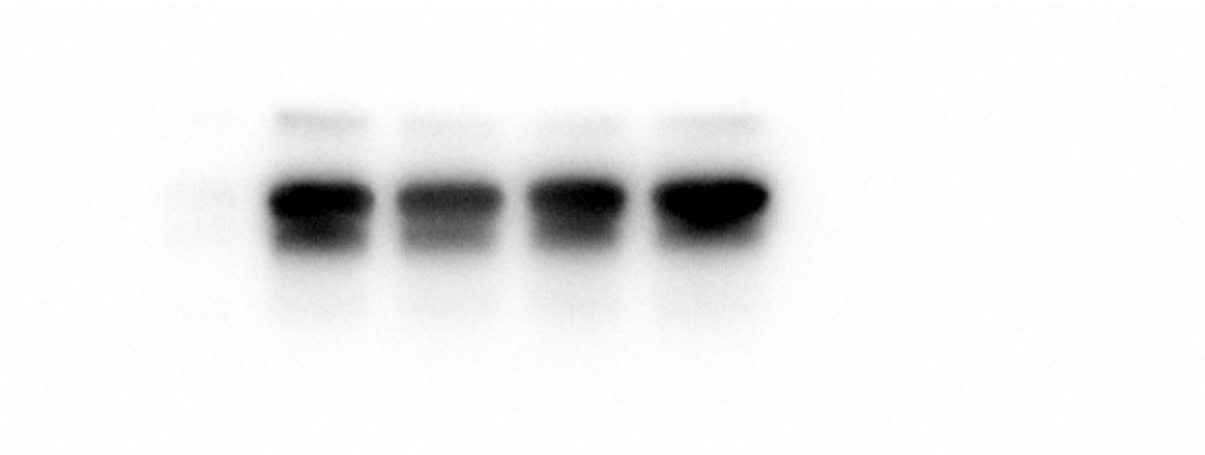

Figure 4D – pSTAT3 Y705

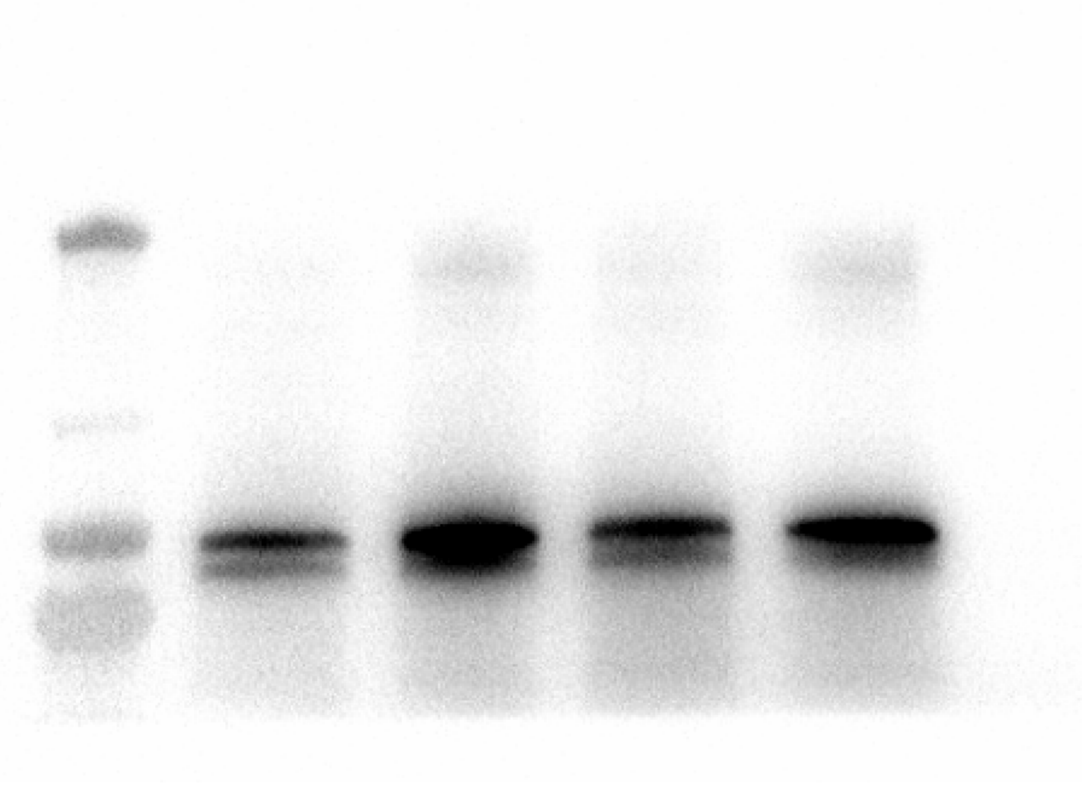

Figure 4D – TBP

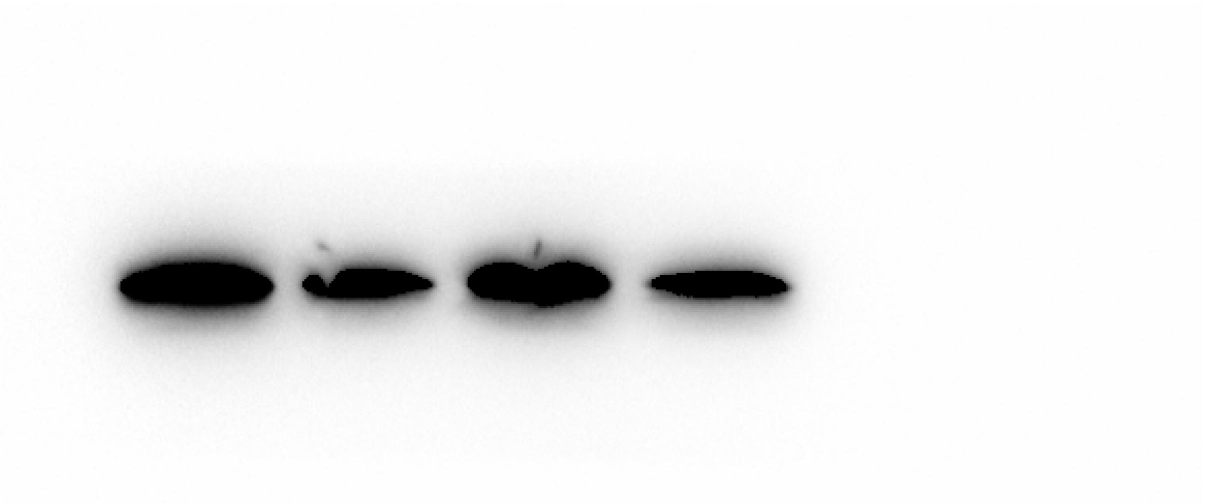

Figure 5A – pSTAT3 Y705

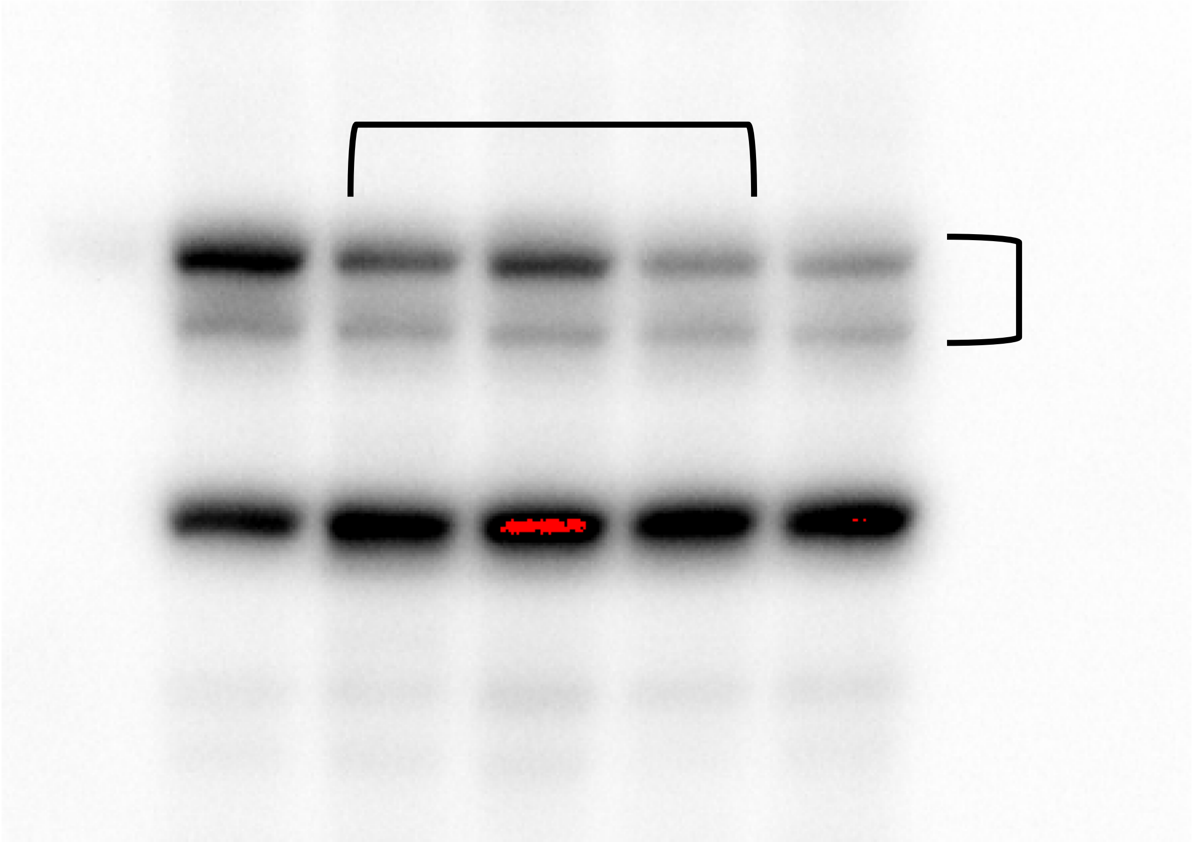

Figure 5A – Lamin A/C

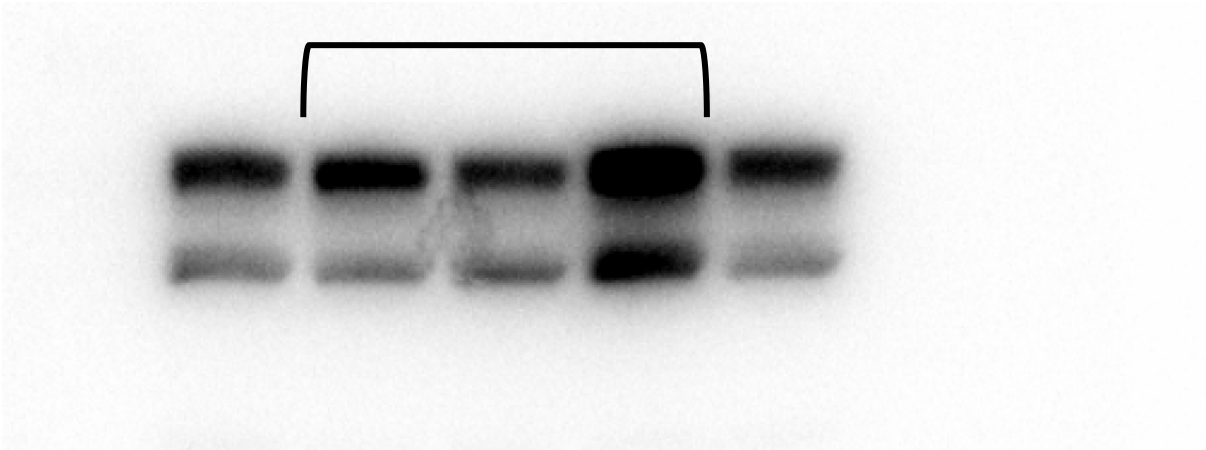

Figure 5C - ΔNp63

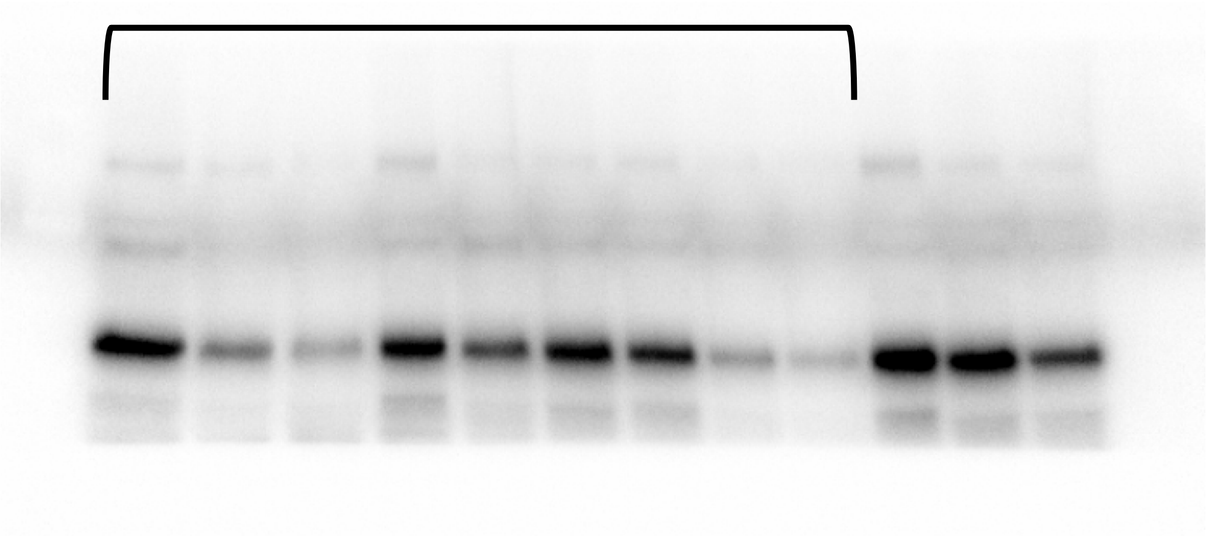

Figure 5C - Involucrin

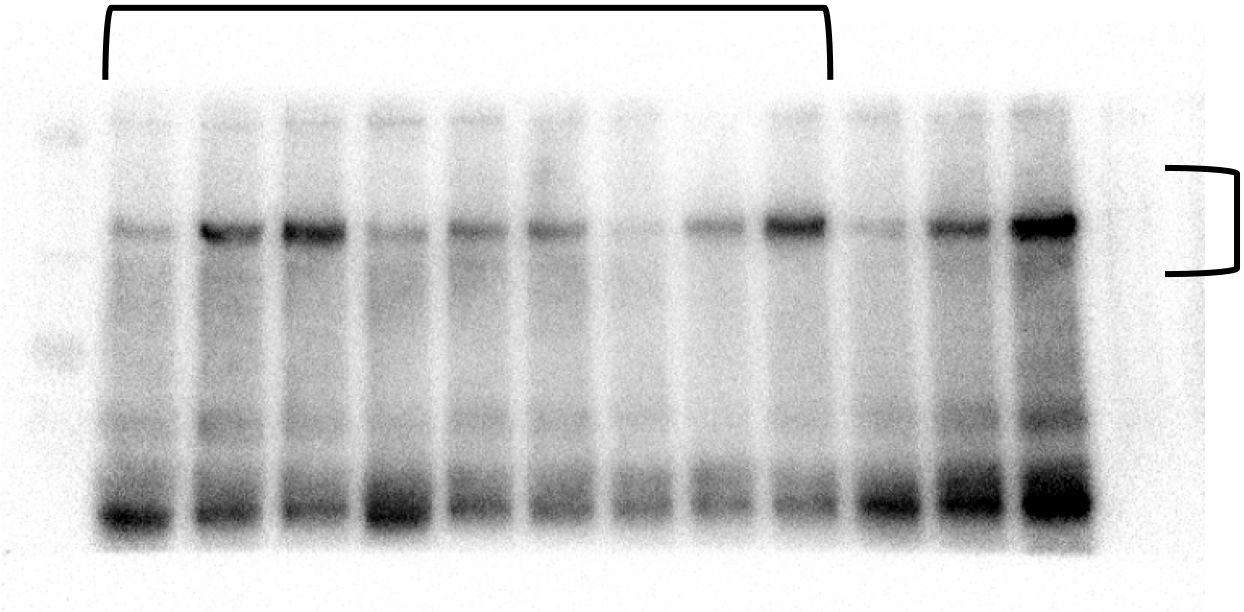

Figure 5C - GAPDH

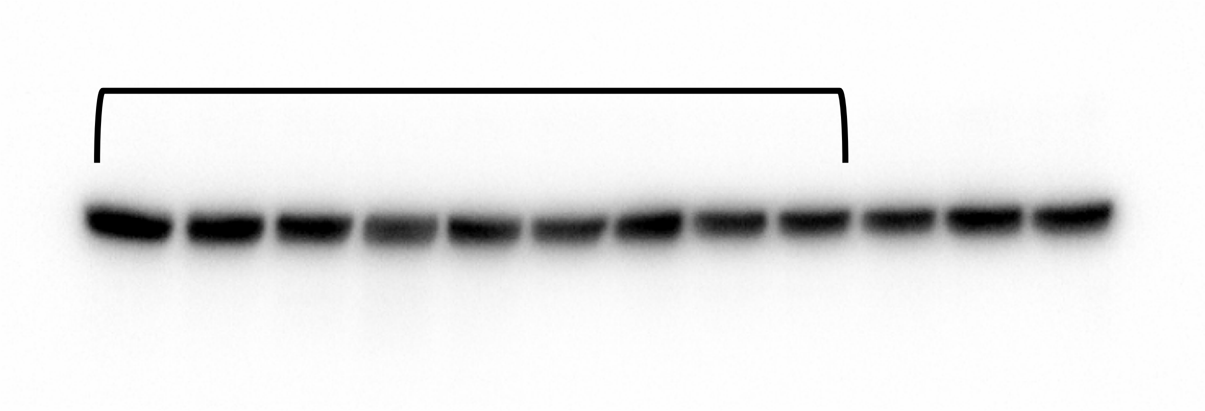

Figure 5H – P300

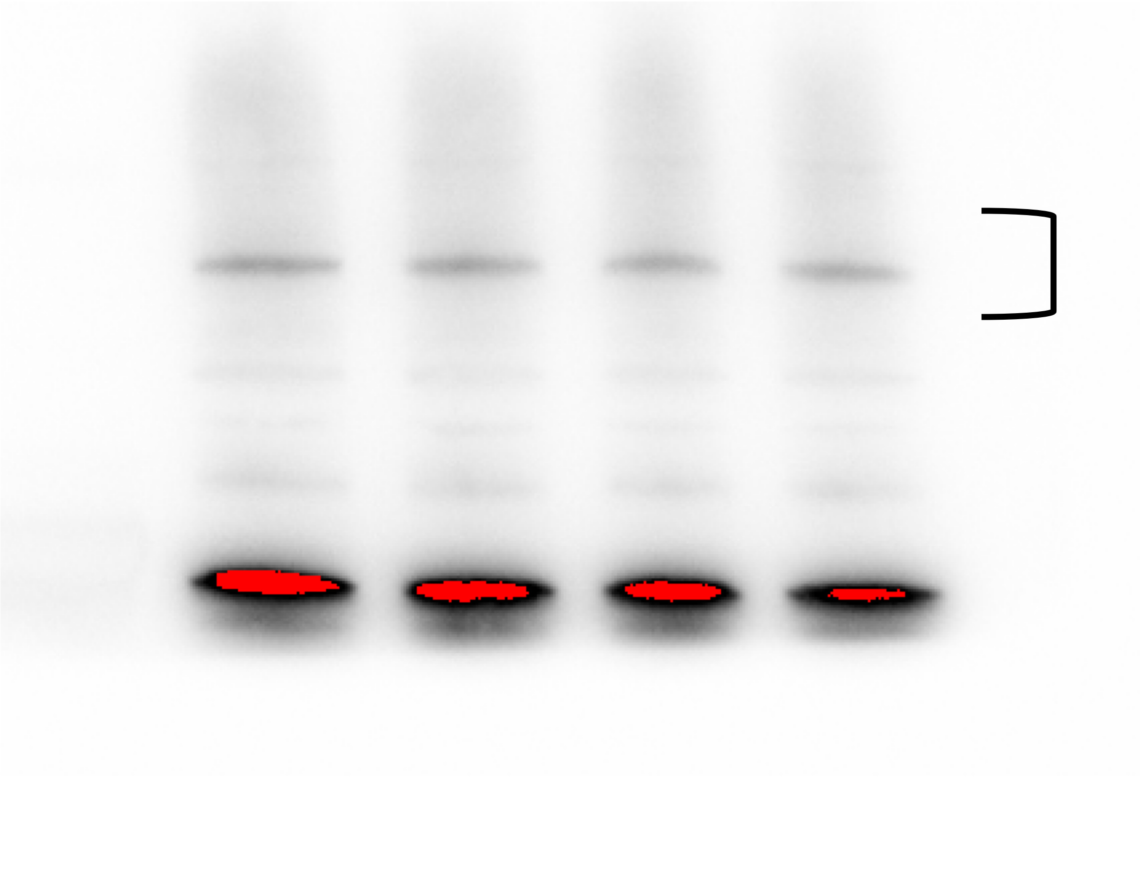

Figure 5H – GAPDH

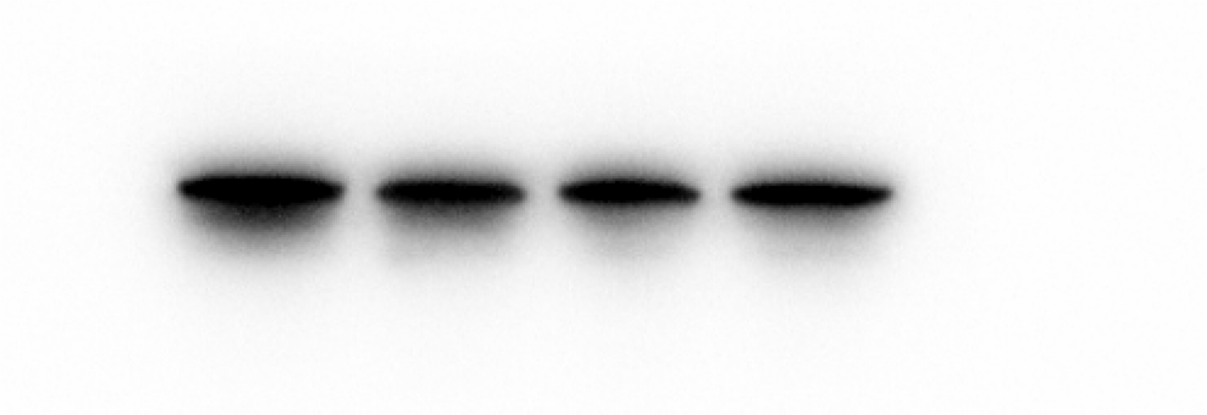

Figure 5J – P300

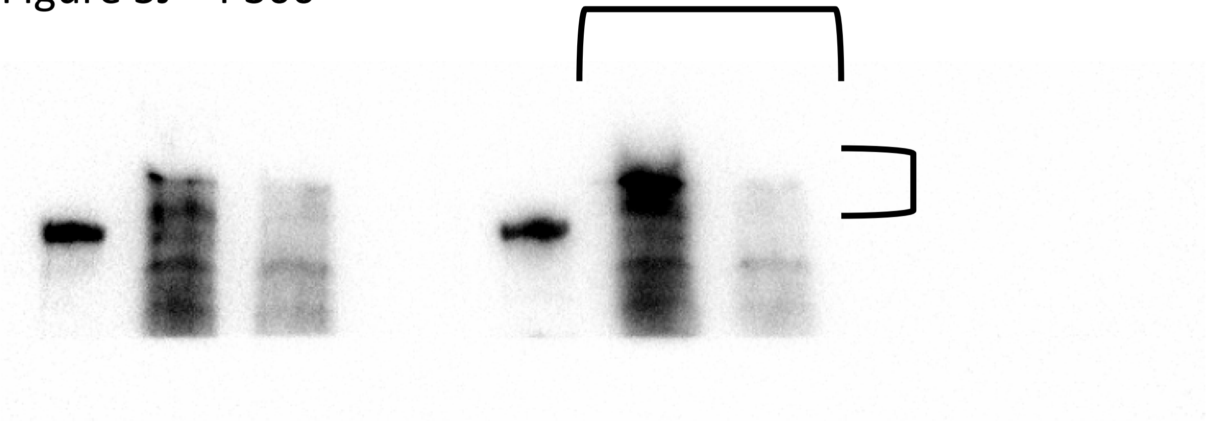

Figure 5J – GAPDH (P300 blot)

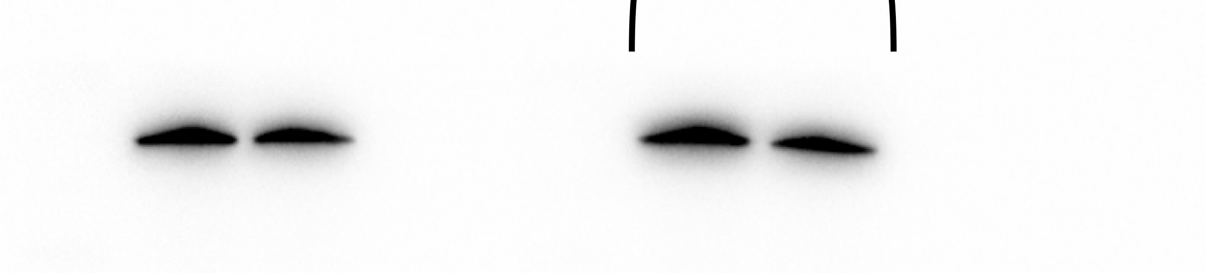

Figure 5J – pSTAT3

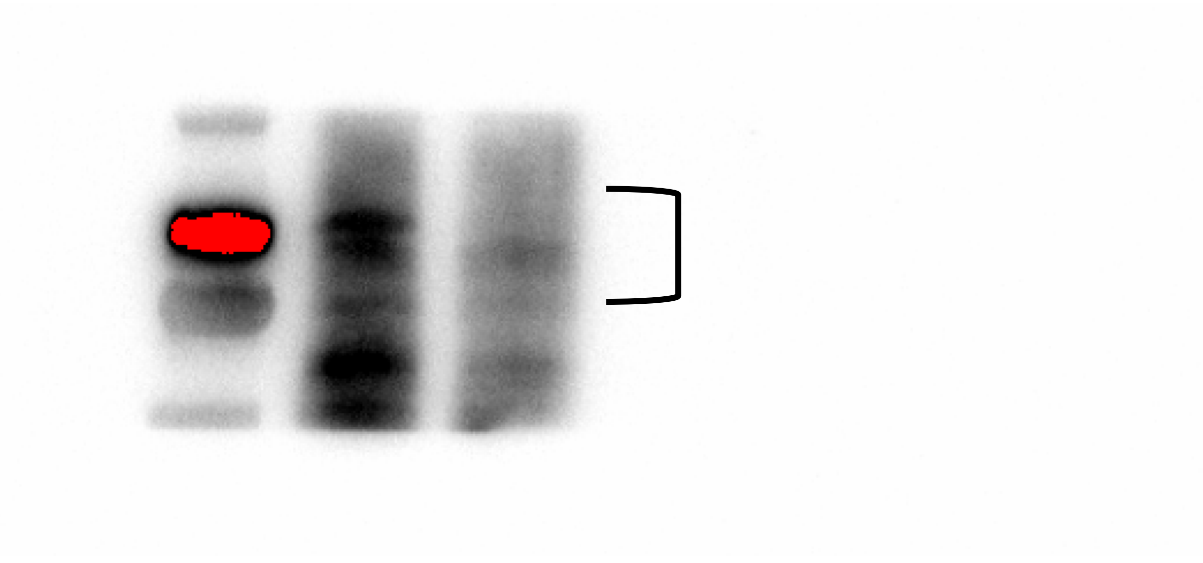

Figure 5J – ΔNp63

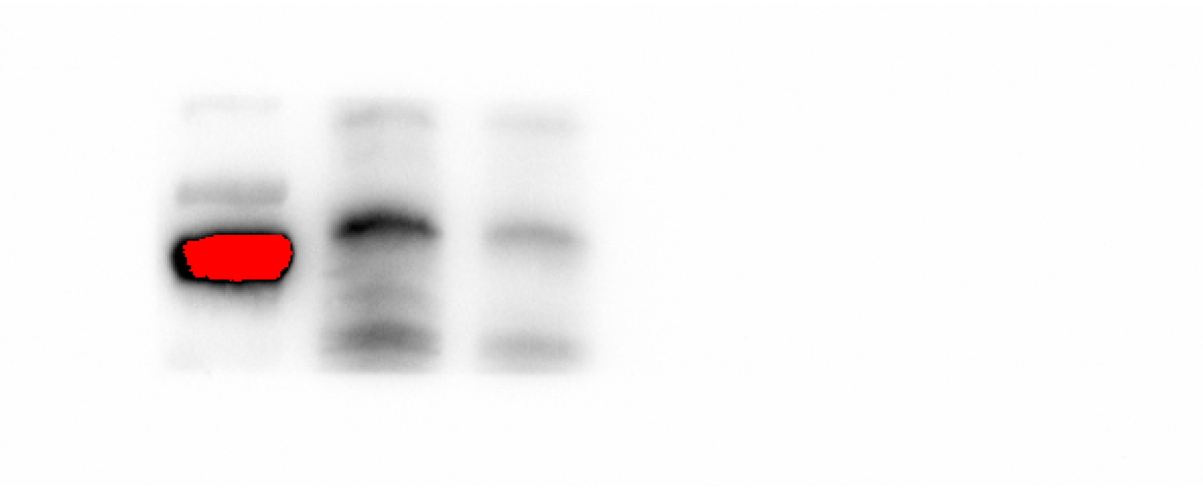

Figure 5J – GAPDH (pSTAT3/ΔNp63 blot)

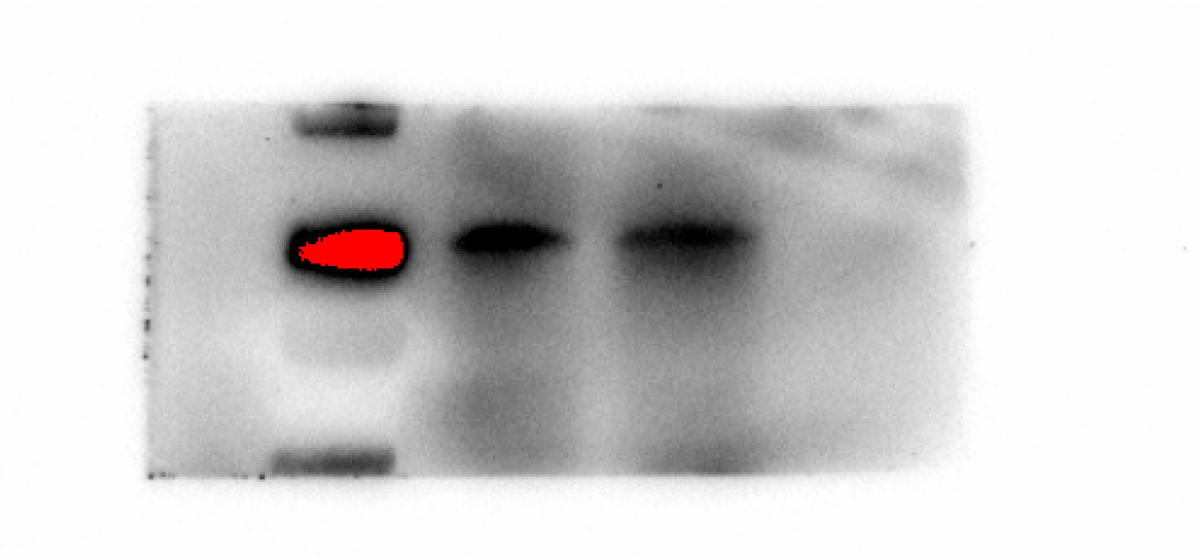

Figure 5K - ΔNp63

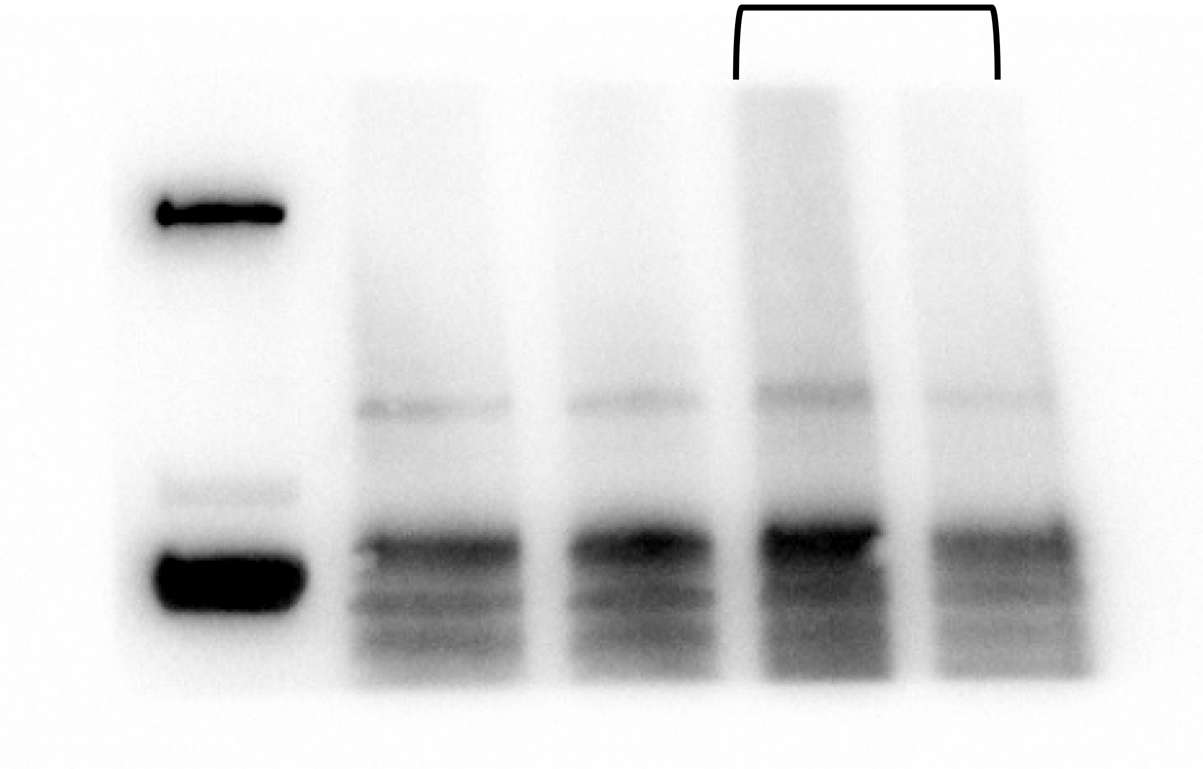

Figure 5K – GAPDH (ΔNp63 blot)

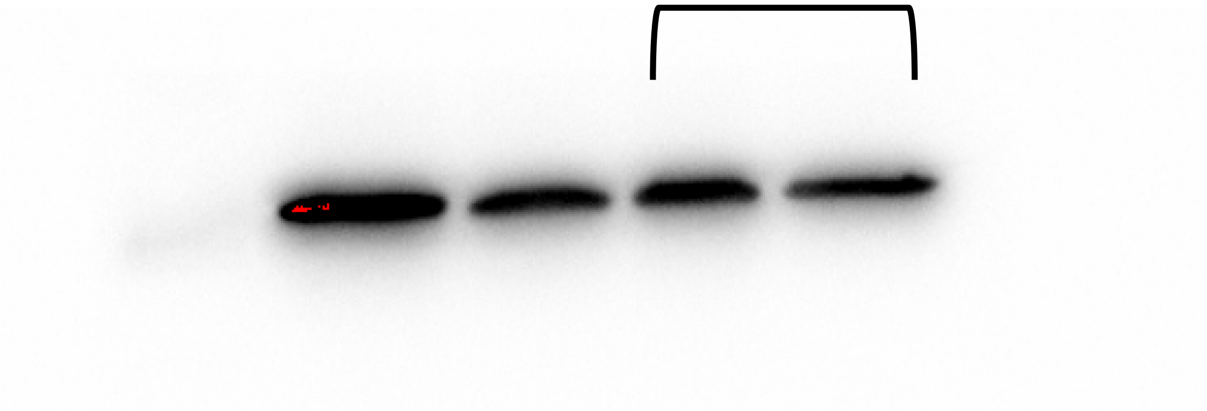

Figure 5K – STAT3

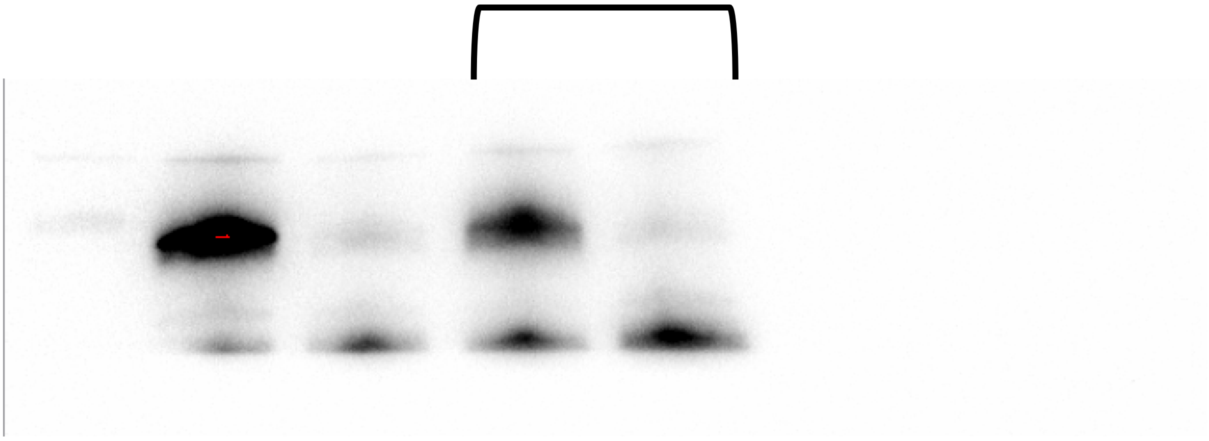

Figure 5K – GAPDH (STAT3 blot)

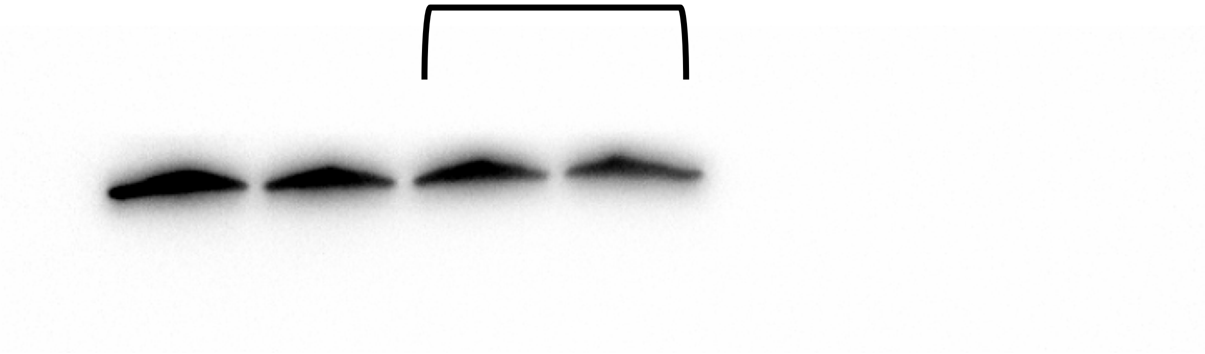

Figure 5L – Acetyl STAT3

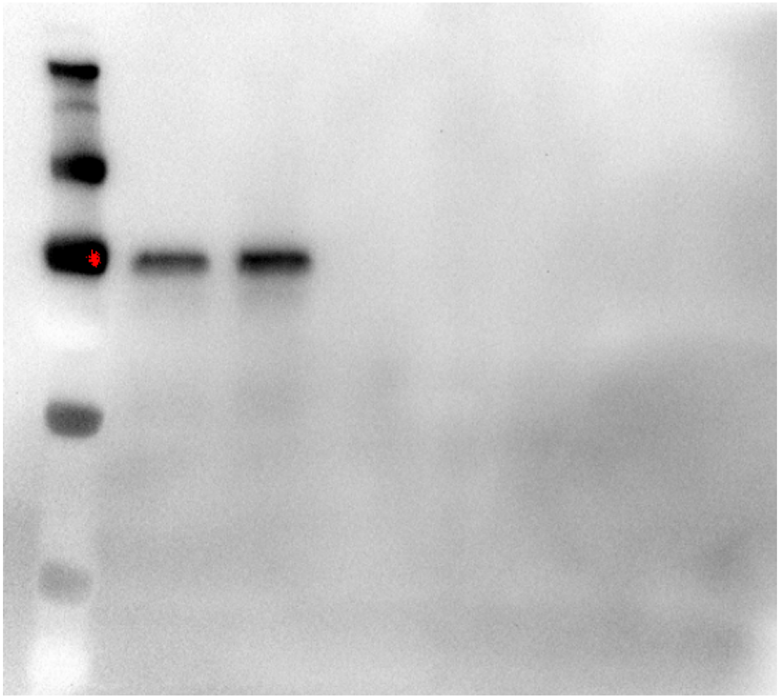

Figure 5L – Total STAT3

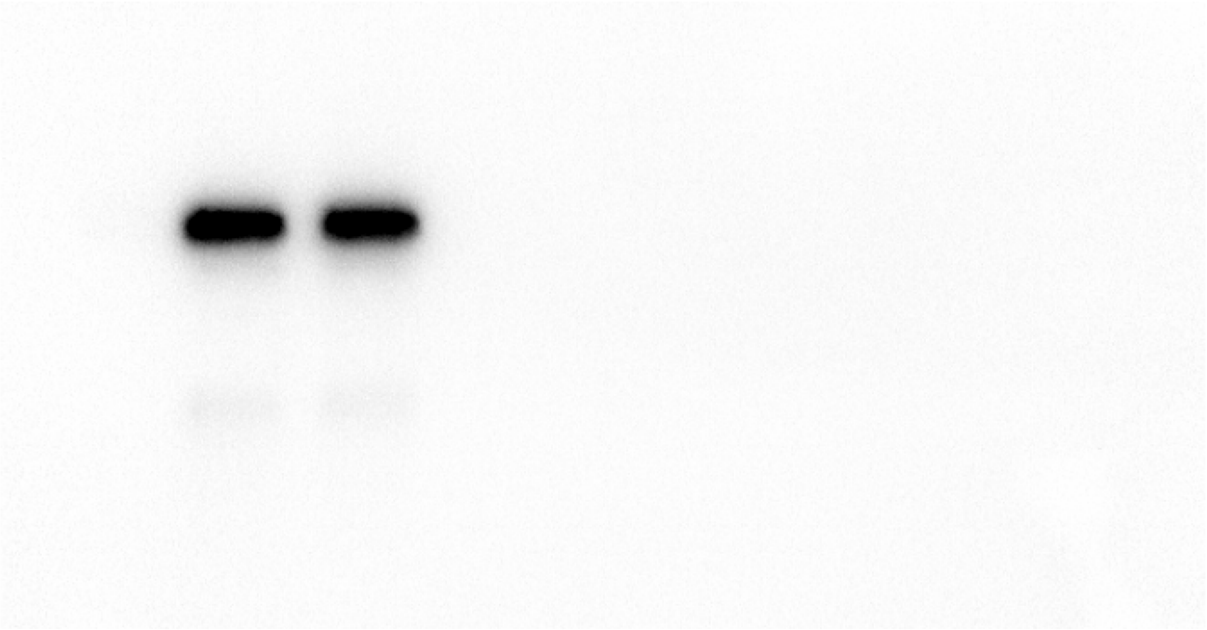

Figure 6A – pSTAT3

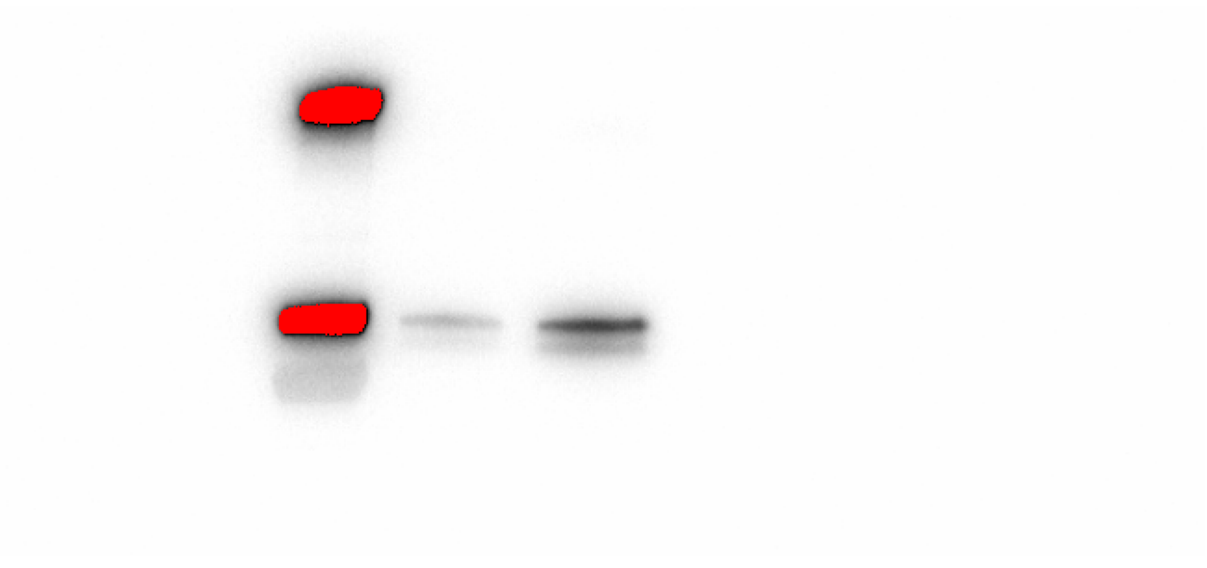

Figure 6A – TBP

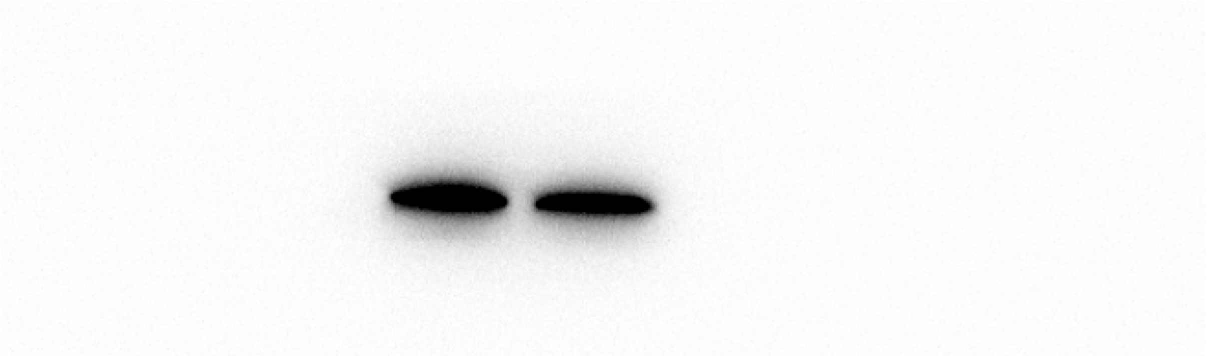

Figure 6A – ΔNp63

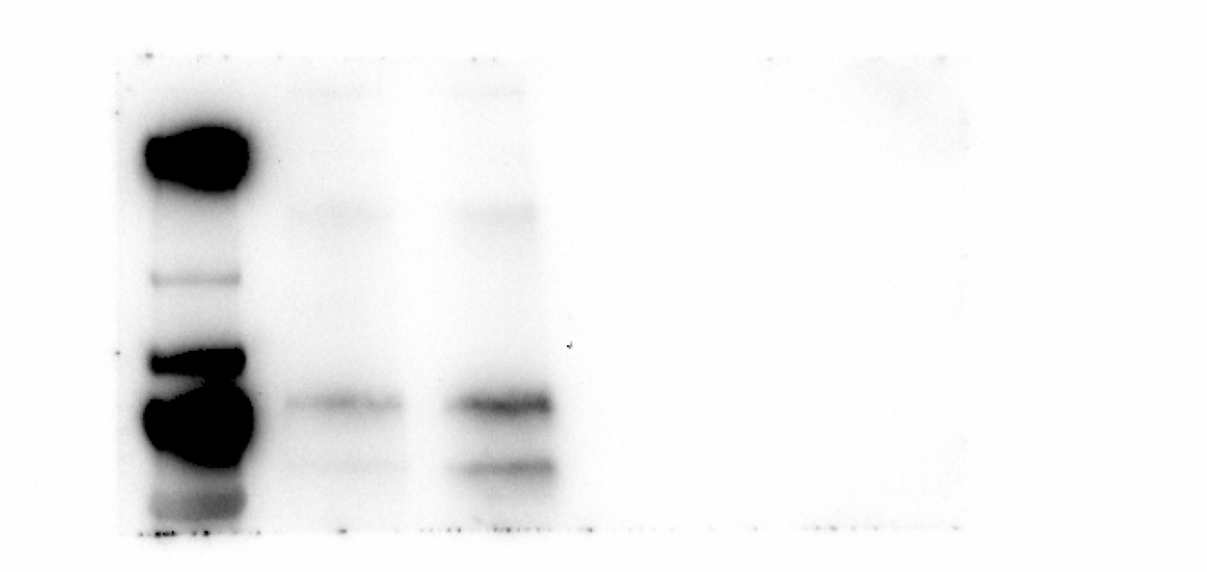

Figure 6A – YAP

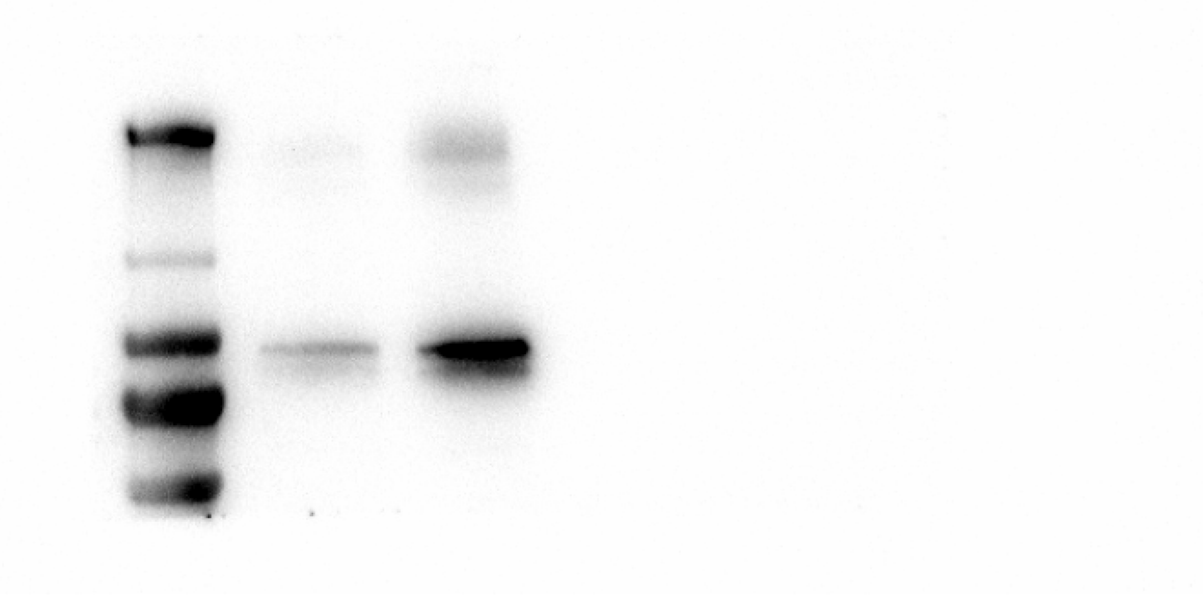

Figure 6A – TBP (ΔNp63/YAP blot)

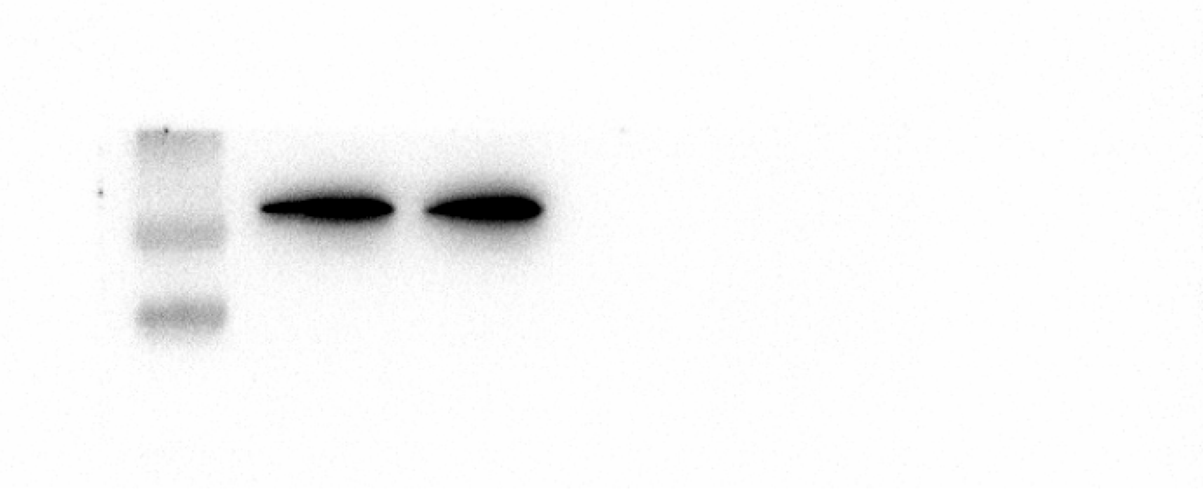

Figure 6B - pSTAT3

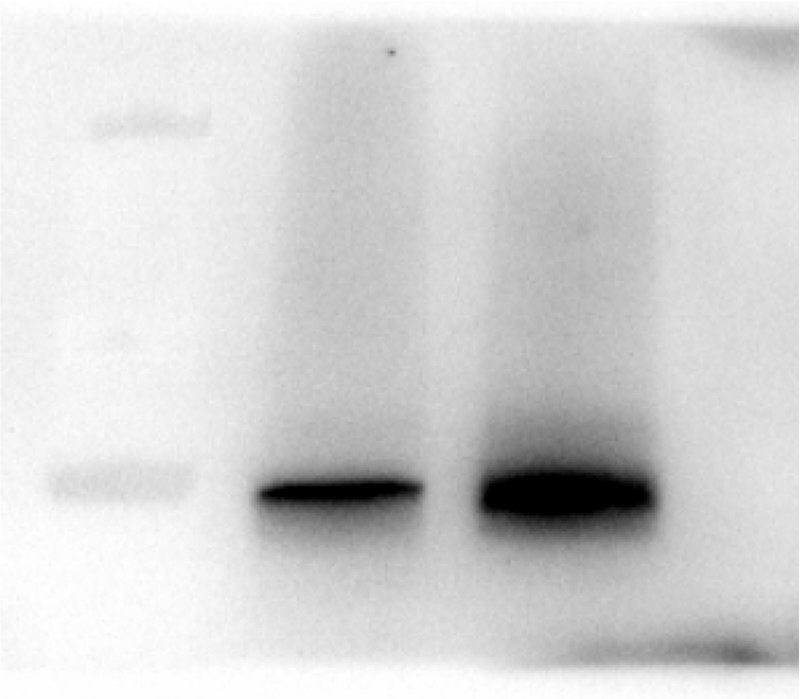

Figure 6B - YAP

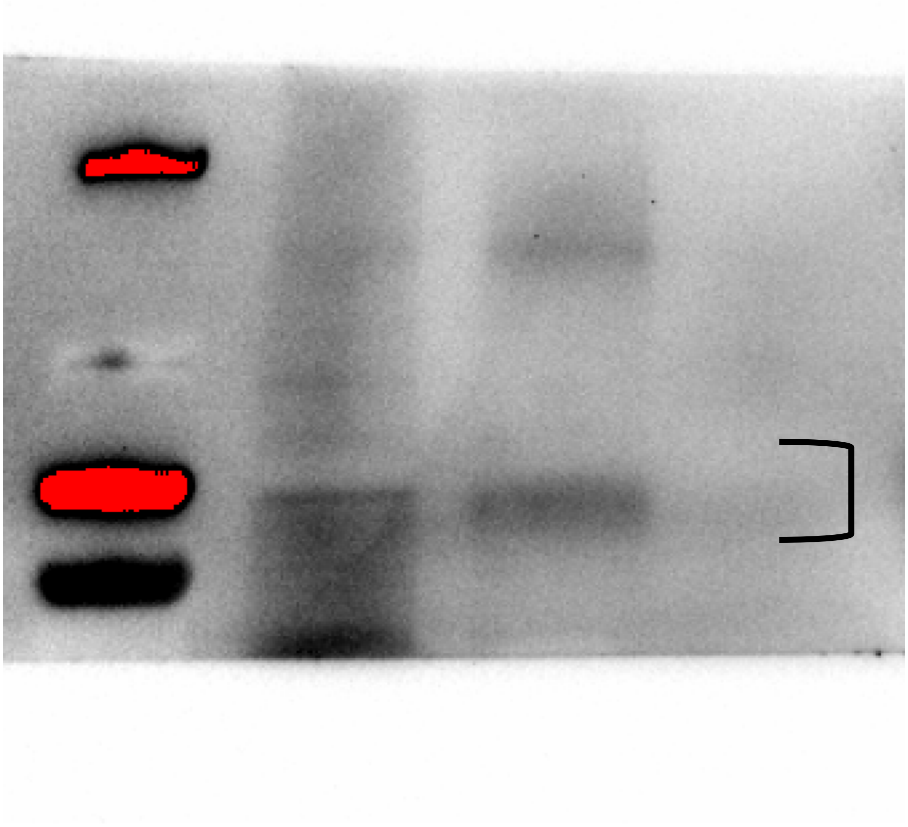

Figure 6B - TBP (pSTAT3/YAP blot)

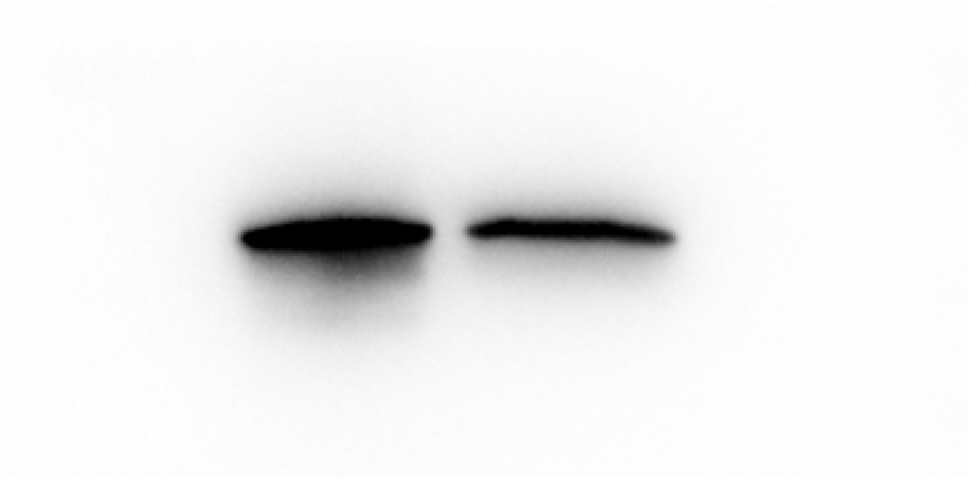

Figure 6B - ΔNp63

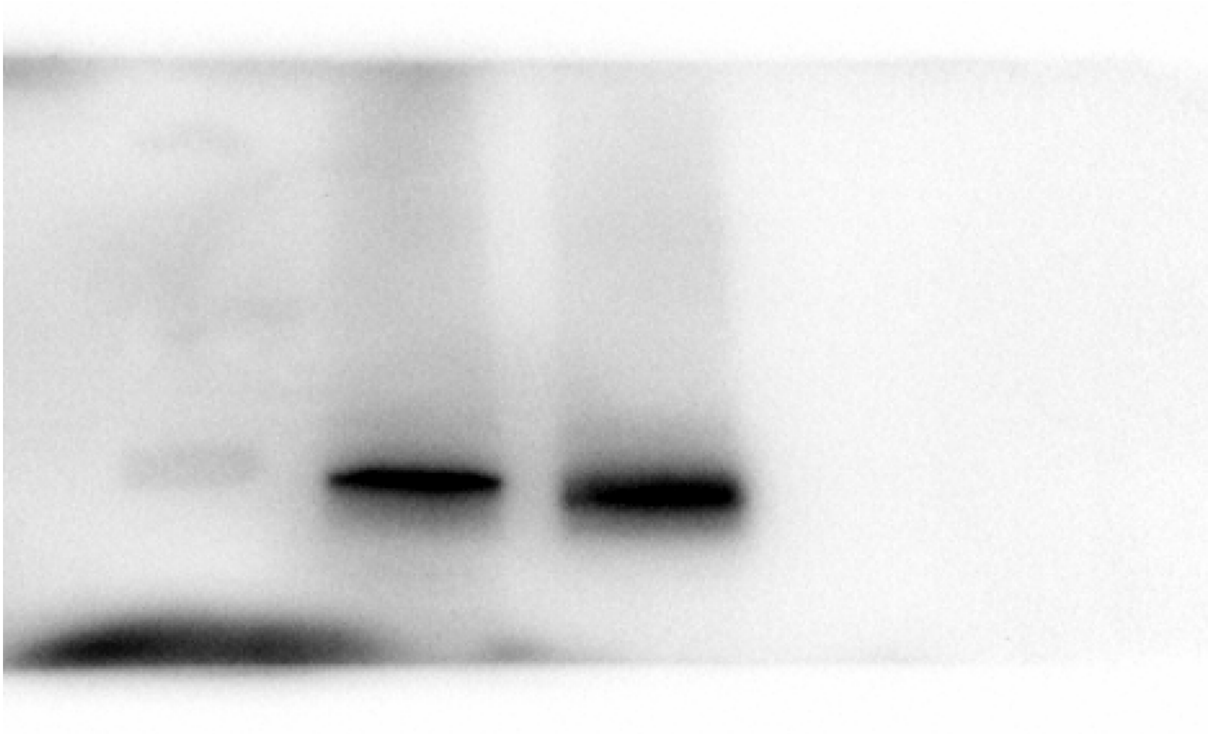

Figure 6B - TBP (ΔNp63 blot)

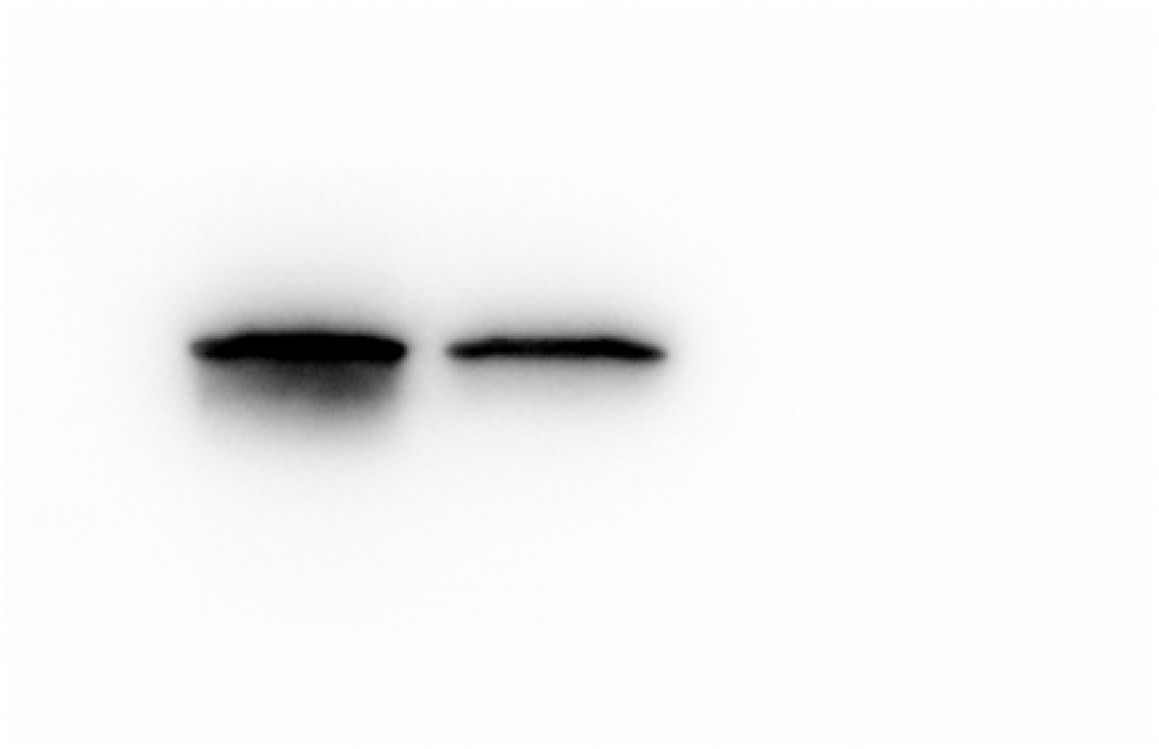

Figure 6C – pSTAT3

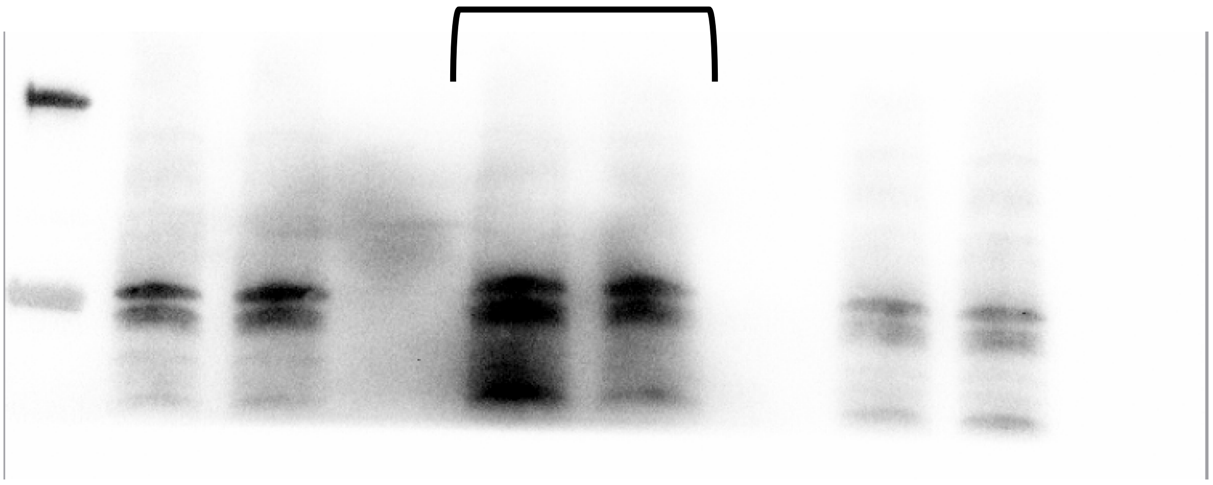

Figure 6C – STAT3

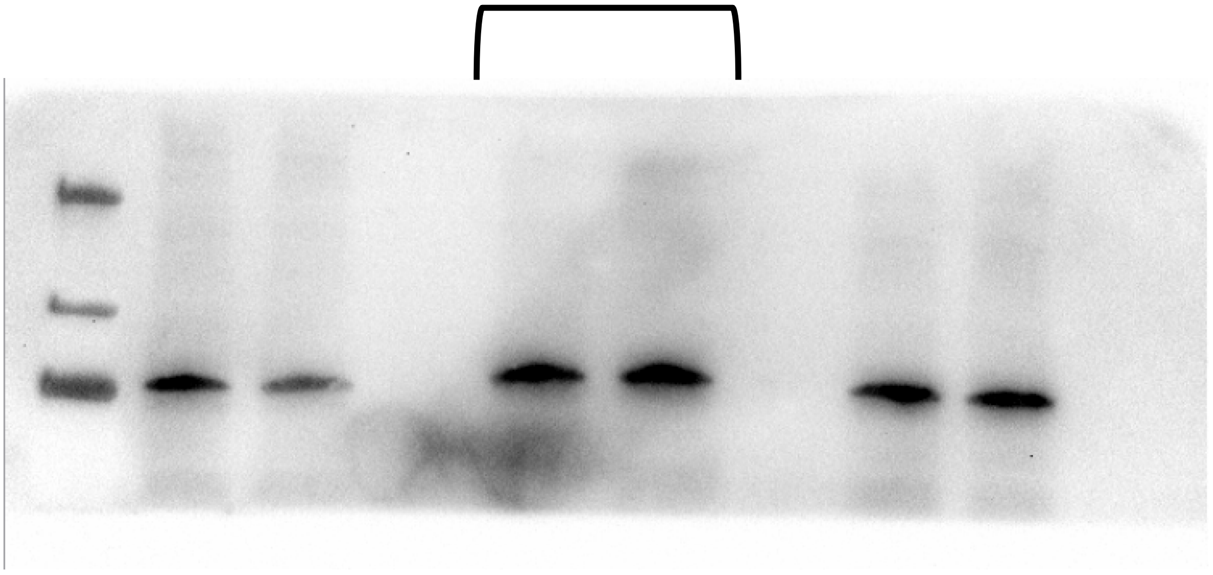

Figure 6C – GAPDH (pSTAT3/STAT3 blot)

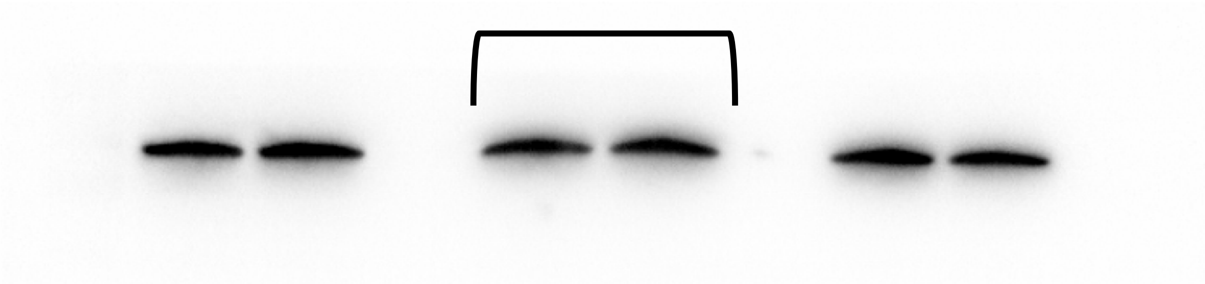

Figure 6C – YAP

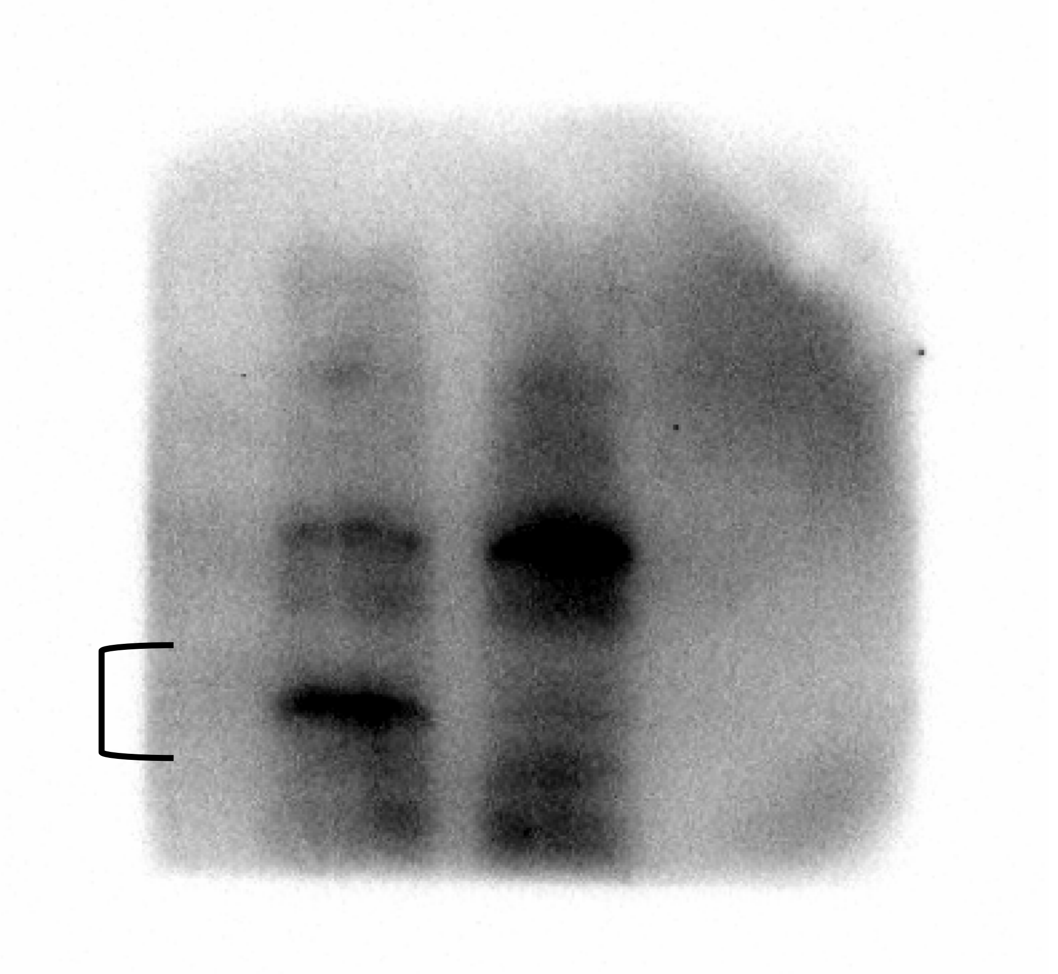

Figure 6C – GAPDH (YAP blot)

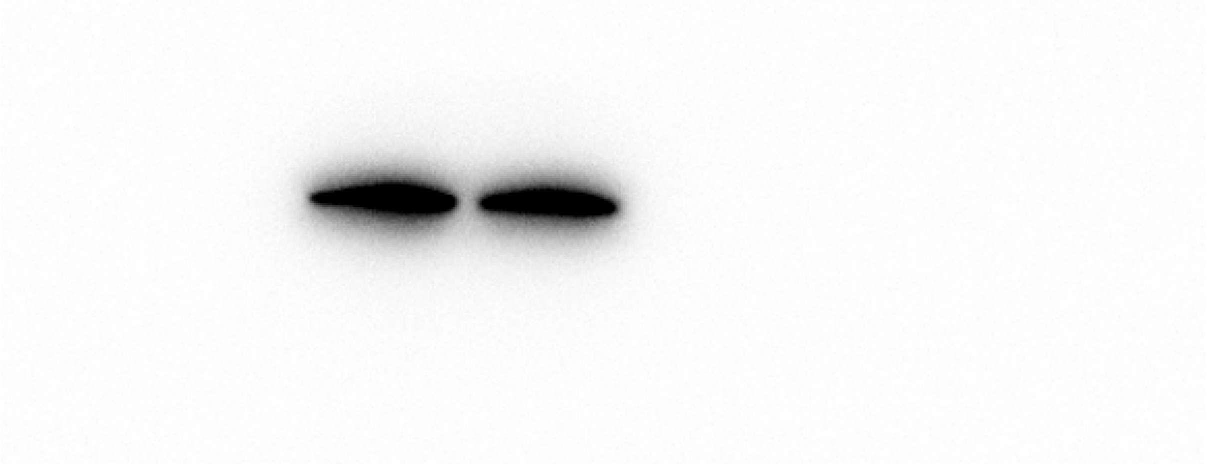

Figure 6E – STAT3

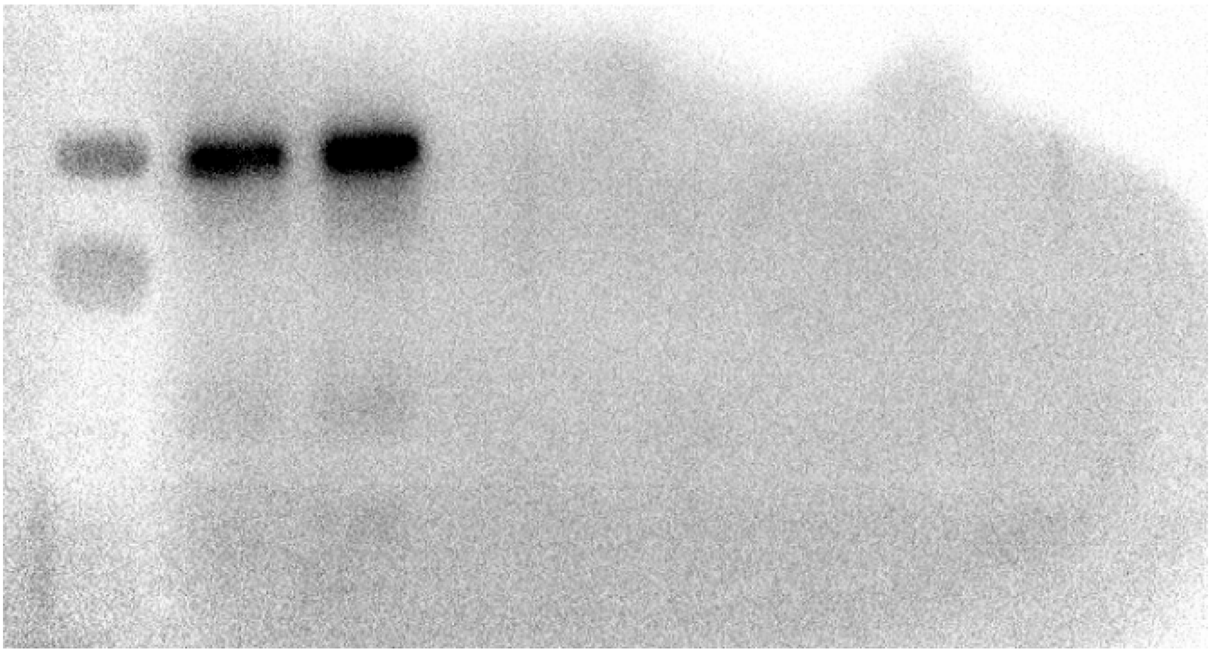

Figure 6E - ΔNp63

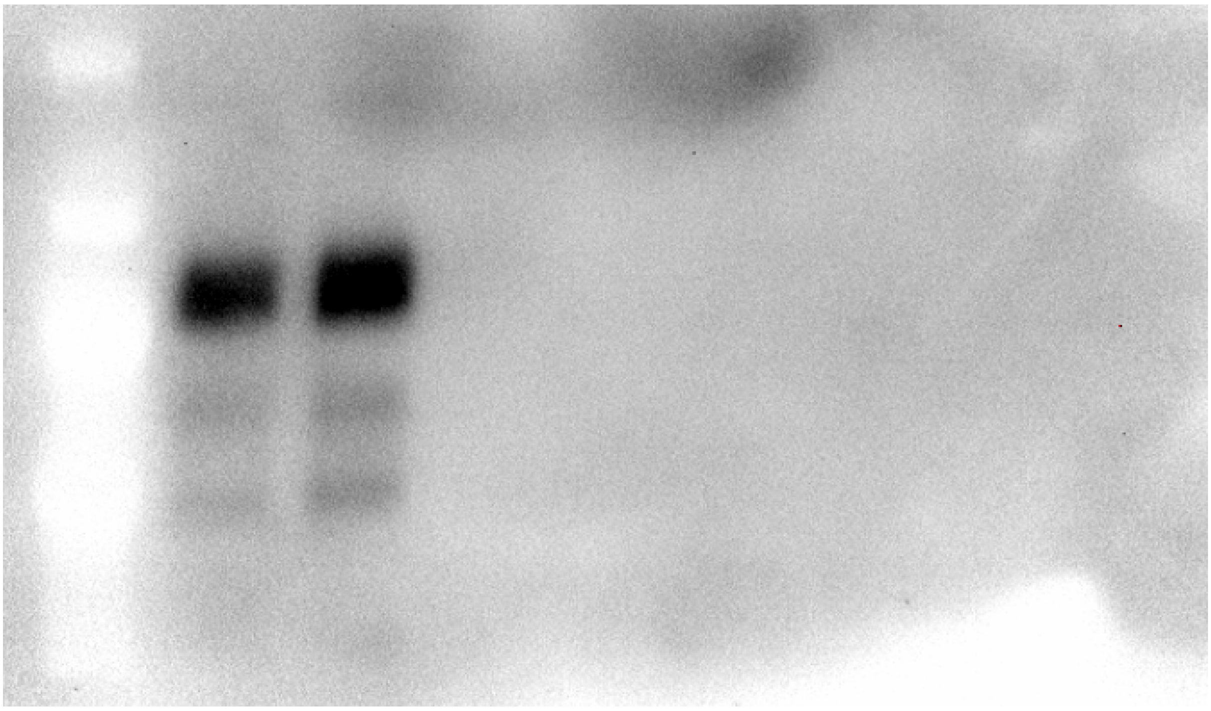

Figure 6E - YAP

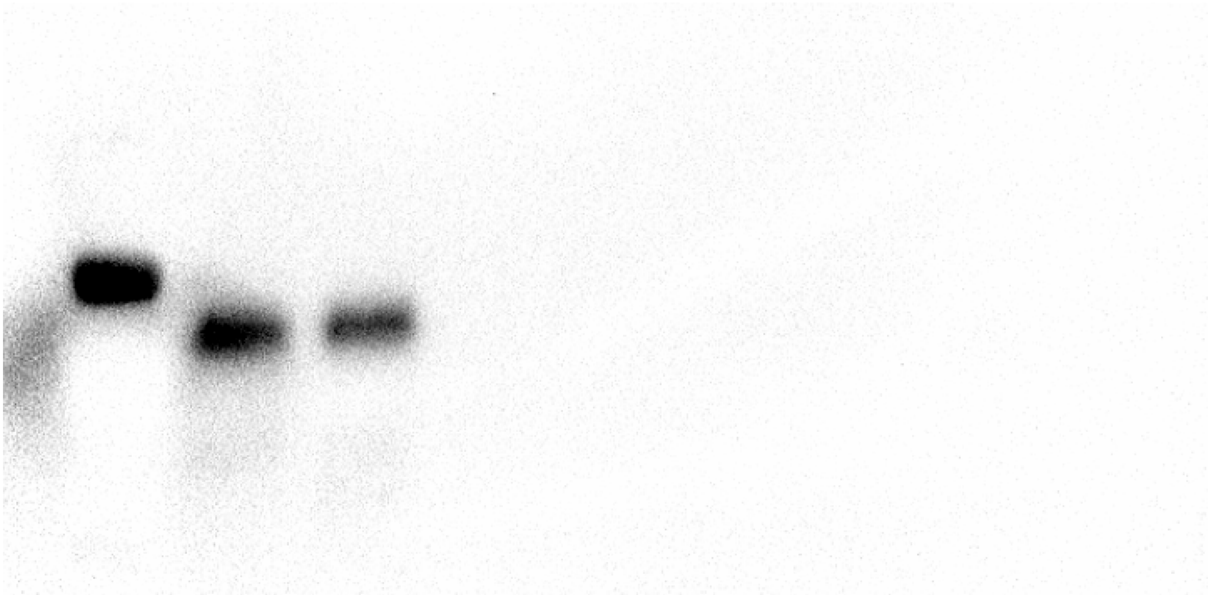

Supplemental Figure 4E - pSTAT3

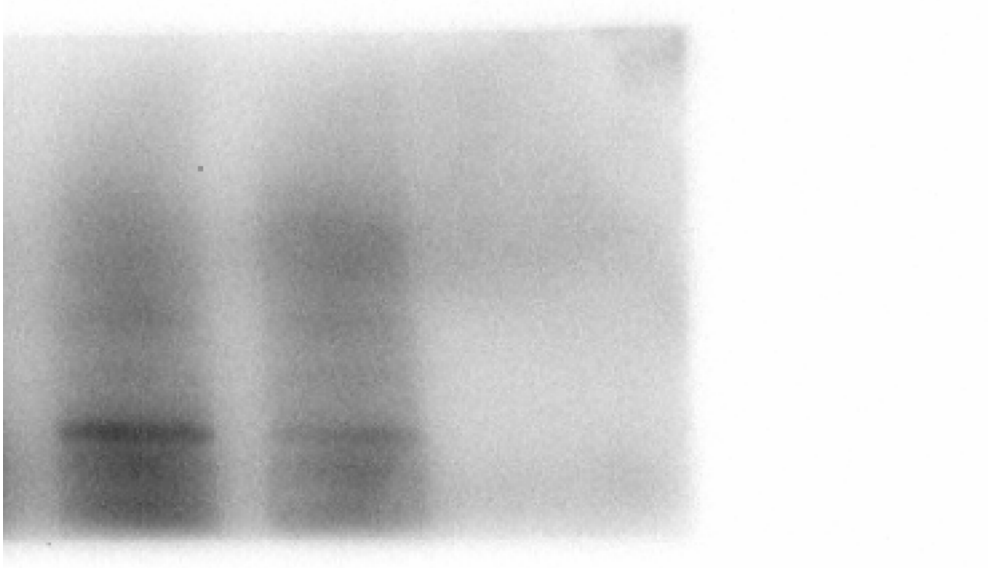

Supplemental Figure 4E - Total STAT3

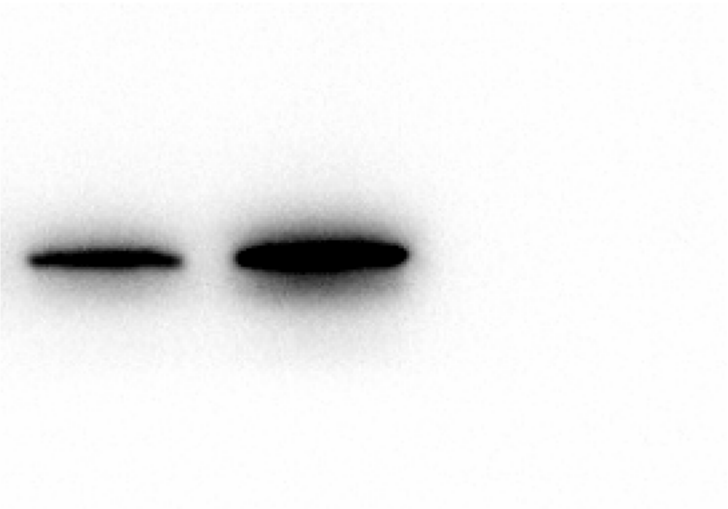

Supplemental Figure 4E - GAPDH

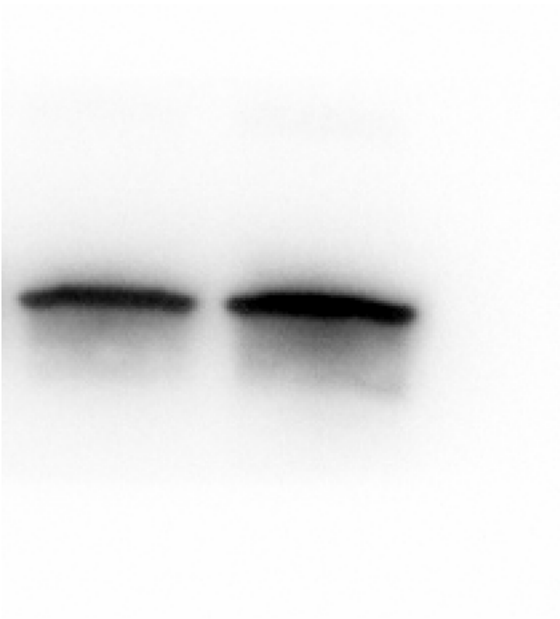

Supplemental Figure 4K – P300

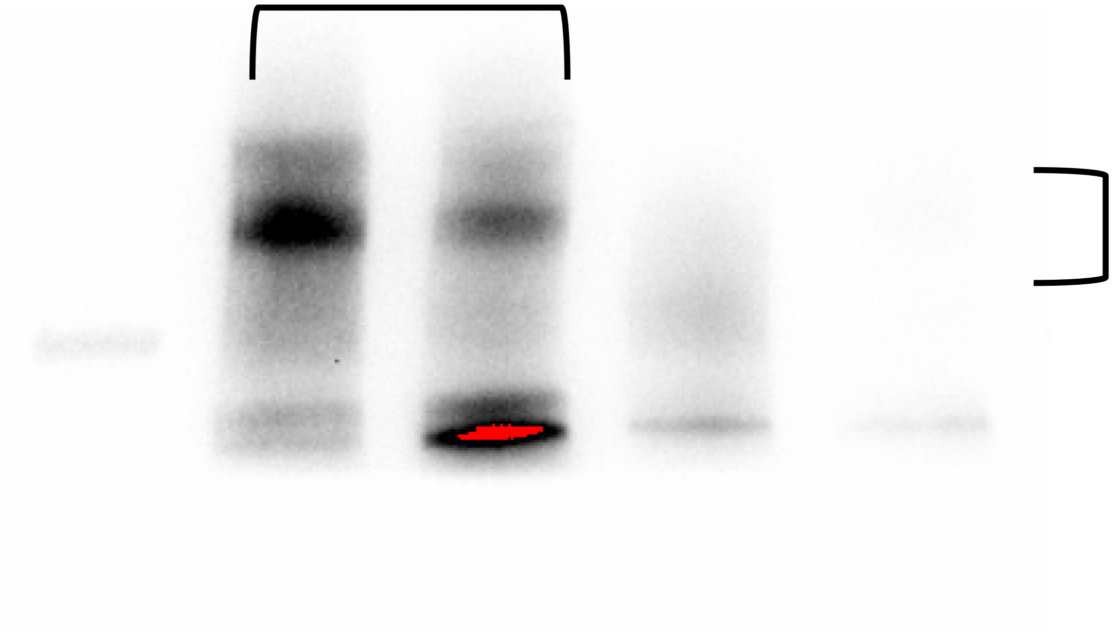

Supplemental Figure 4K – pSTAT3

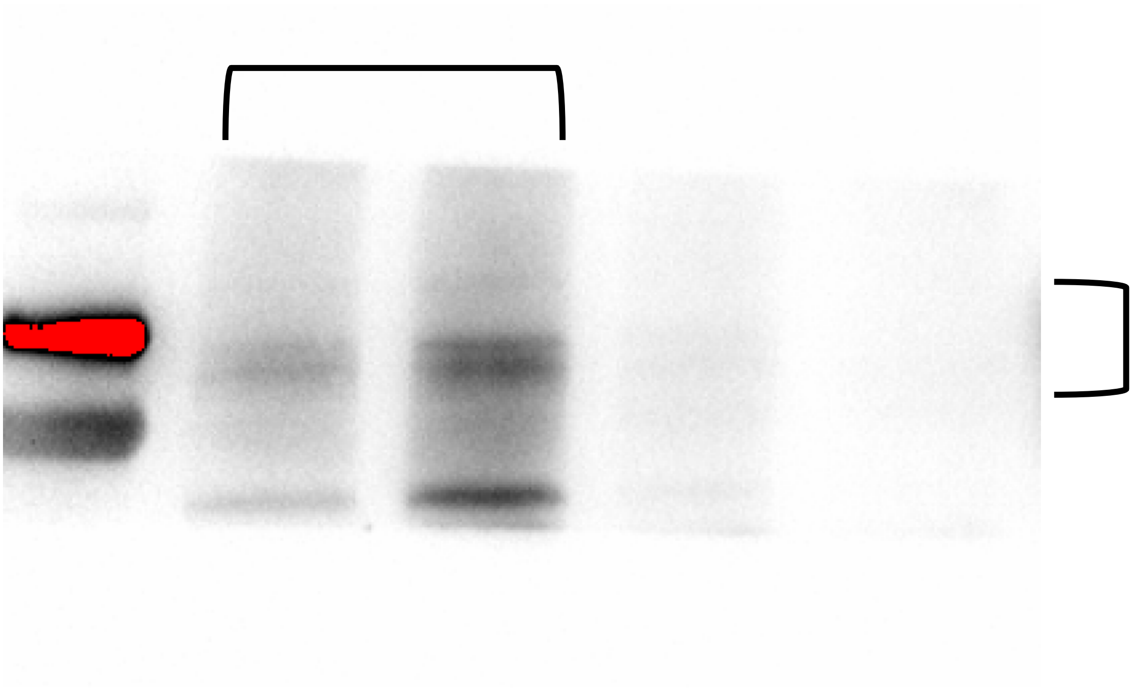

Supplemental Figure 4K – ΔNp63

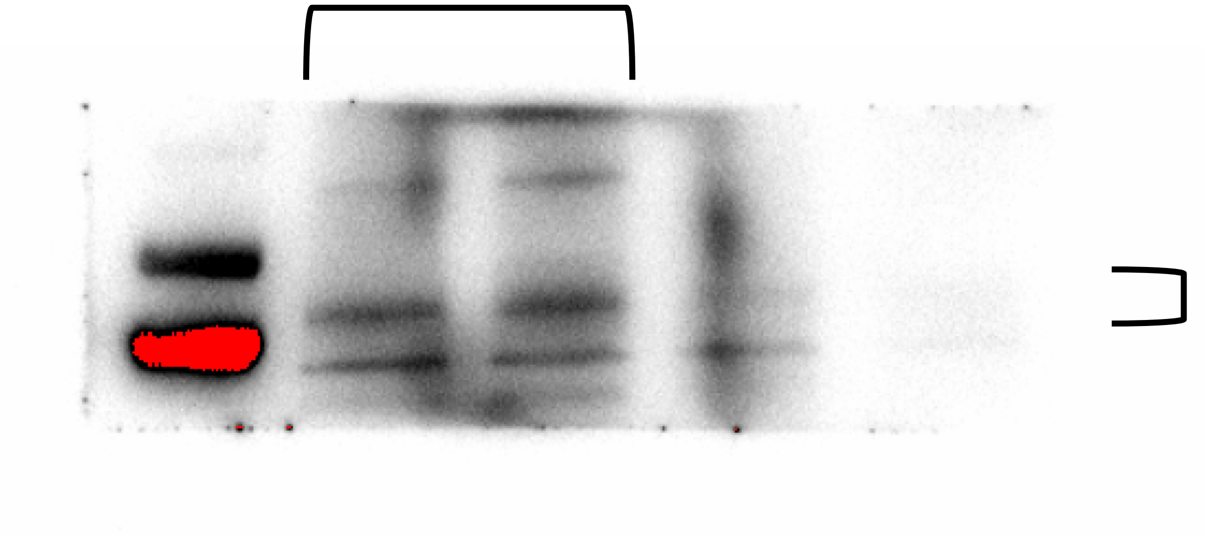

Supplemental Figure 4K – GAPDH

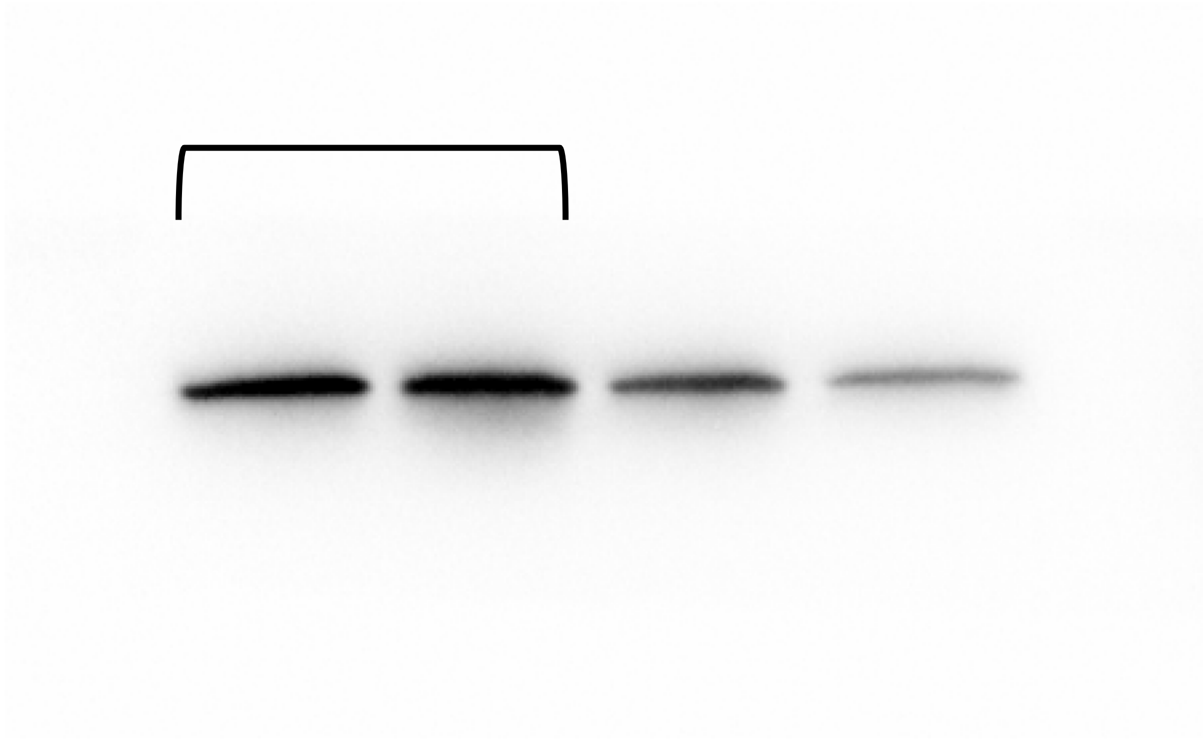

Supplemental Figure 4L – ΔNp63

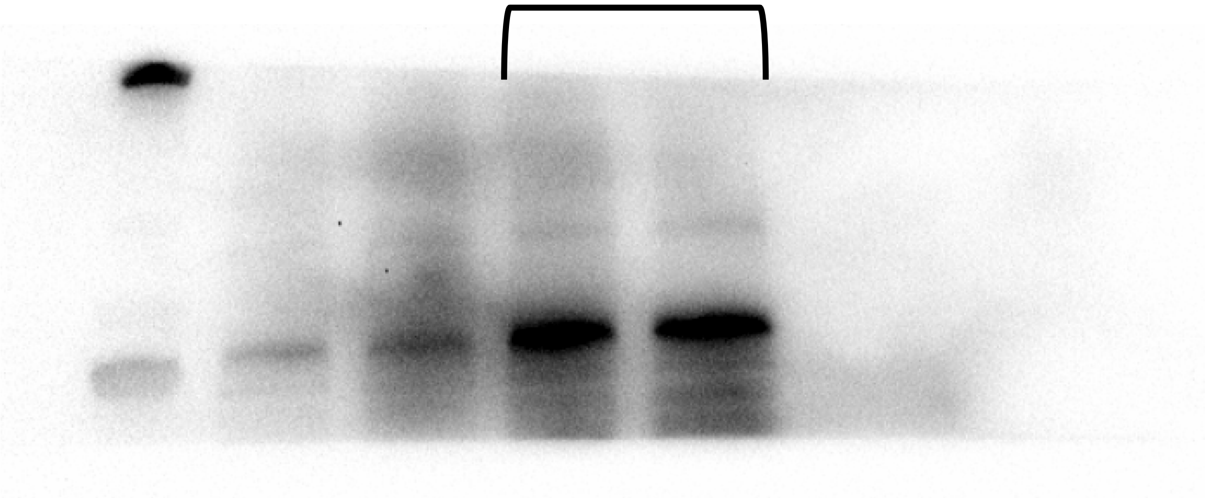

Supplemental Figure 4L – Total STAT3

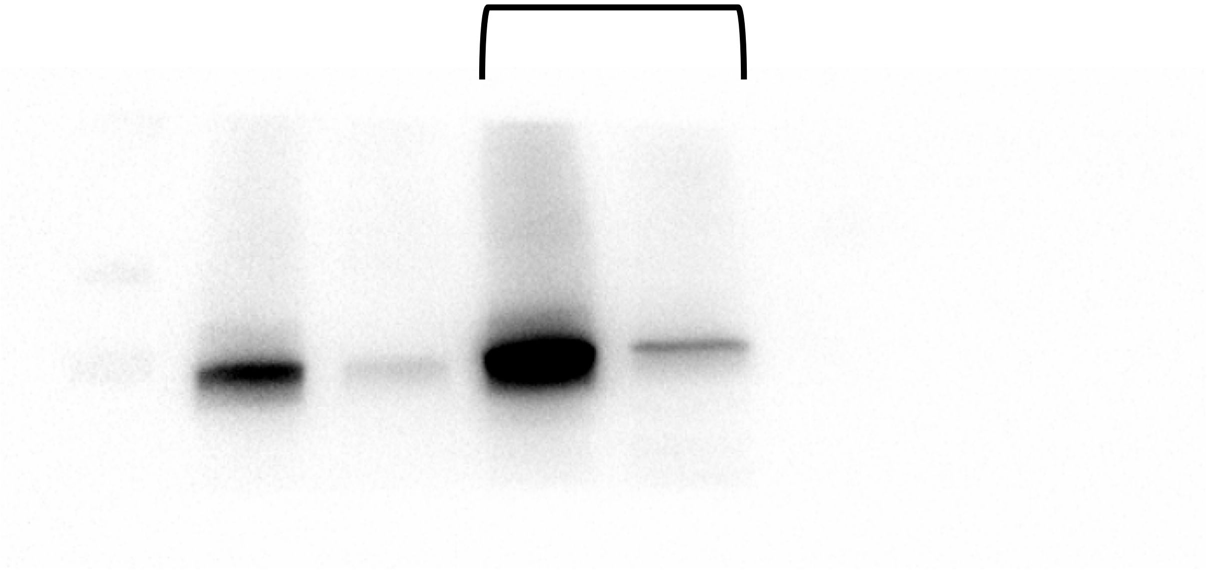

Supplemental Figure 4L – GAPDH

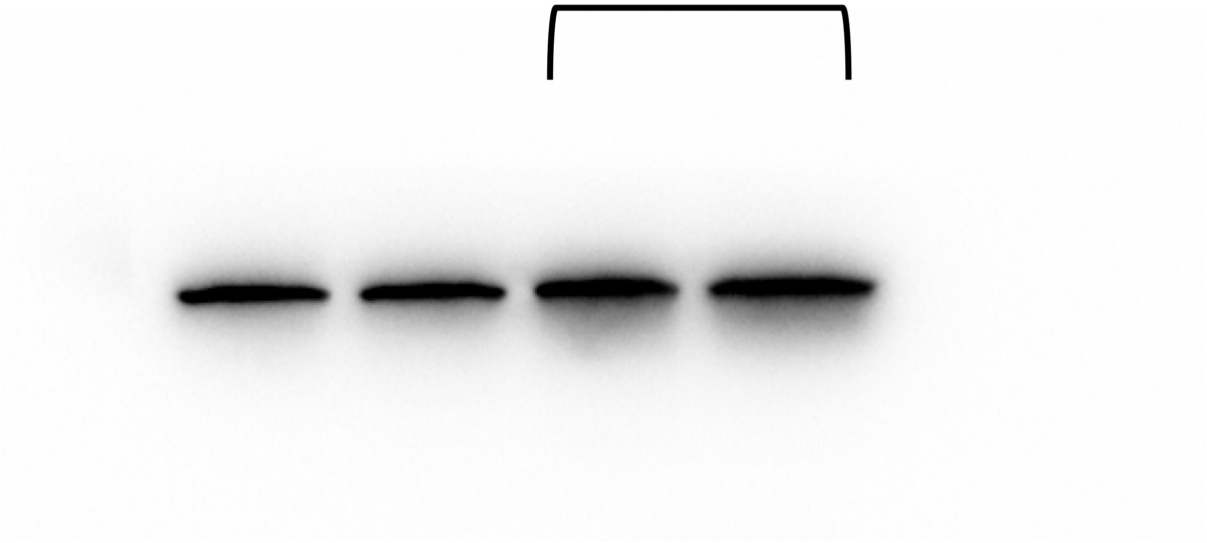

Supplemental Figure 5 – pSTAT3

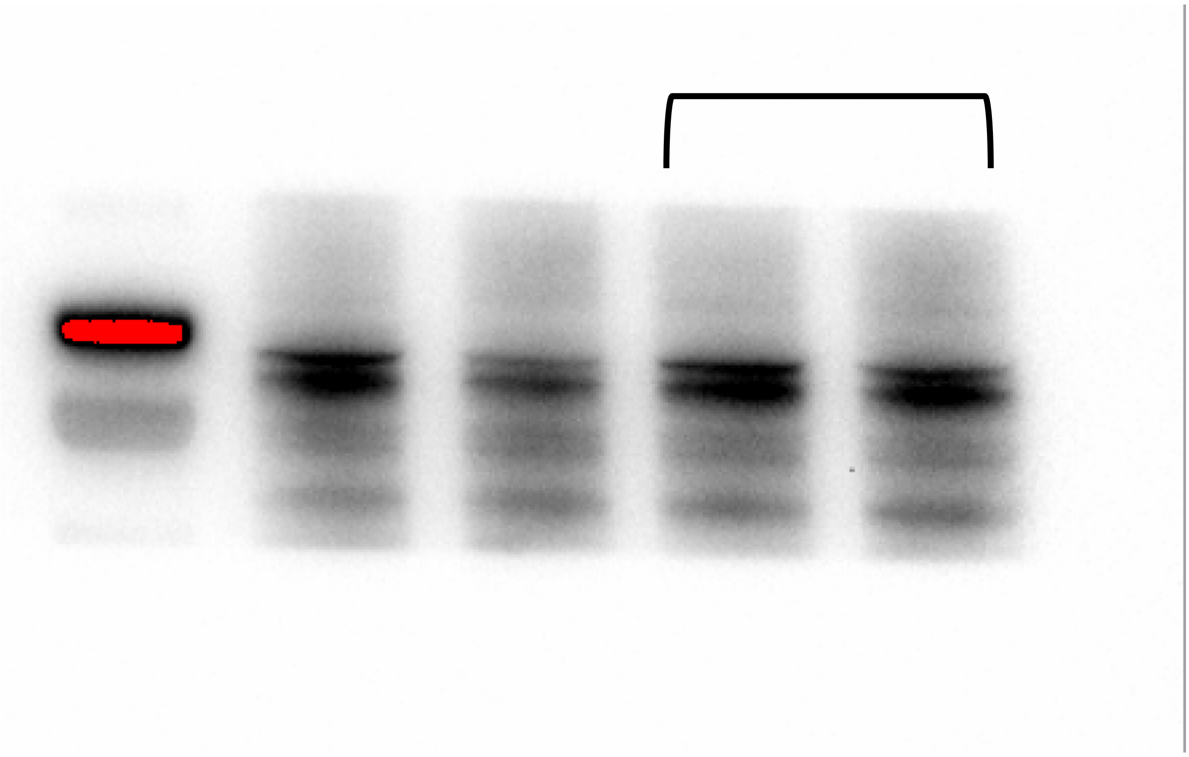

Supplemental Figure 5 – Total STAT3

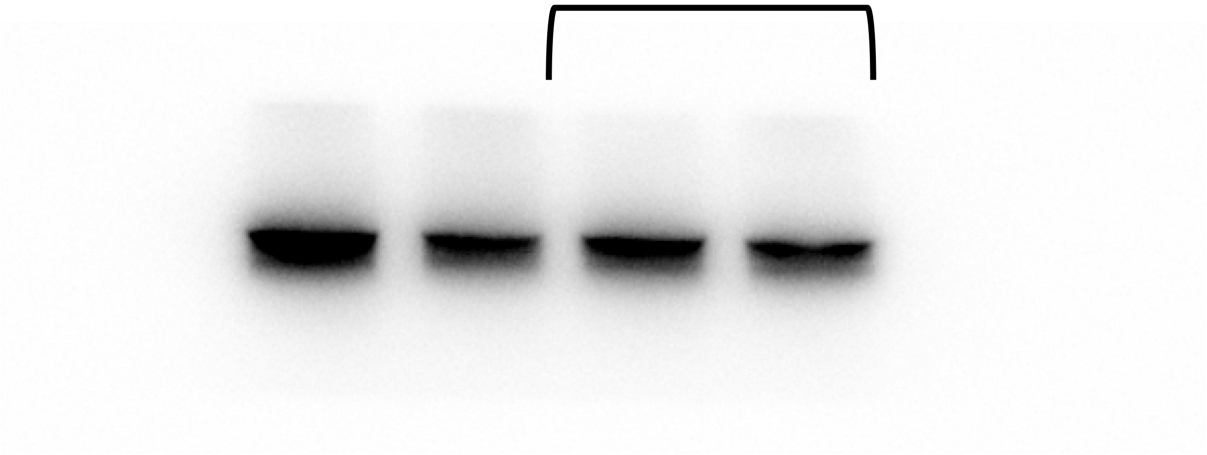

Supplemental Figure 5 – YAP

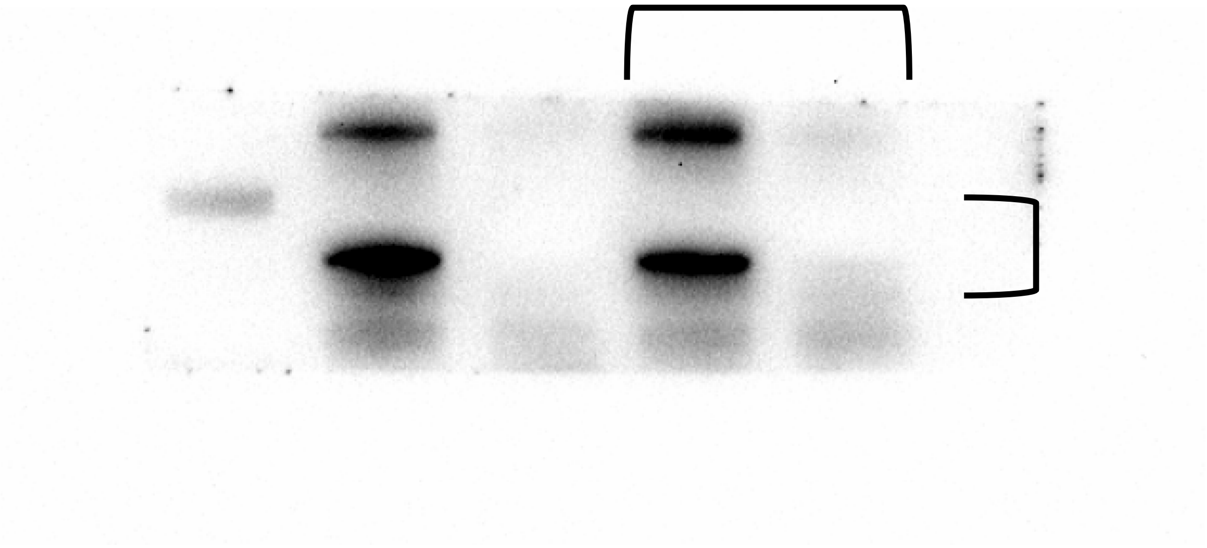

Supplemental Figure 5 – GAPDH

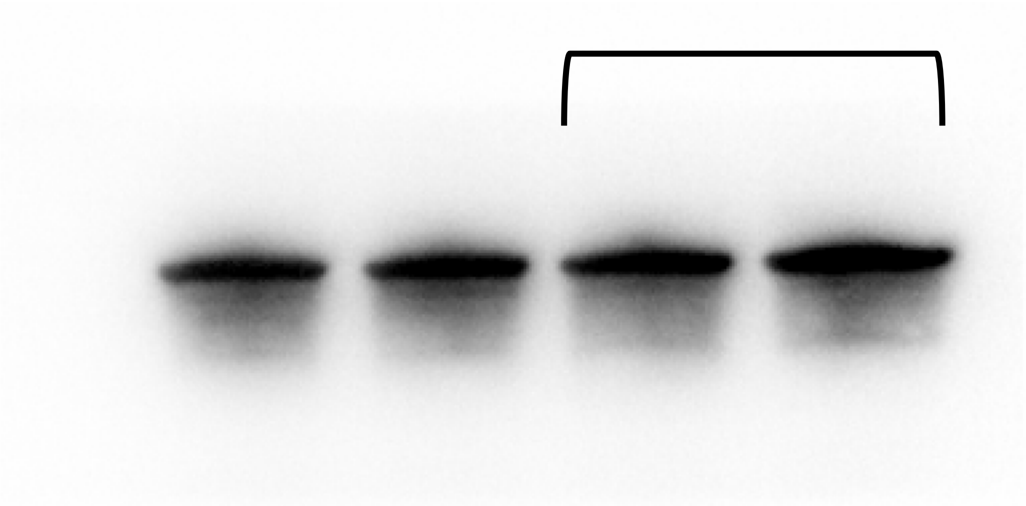

Supplement: Unedited blot and gel images [file jciinsight-9-177898-s165.pdf]
